# Concise Total Syntheses of (*−*)-Crinipellins A and B Enabled by a Controlled Cargill Rearrangement

**DOI:** 10.1021/jacs.4c07900

**Published:** 2024-07-25

**Authors:** Bo Xu, Ziyao Zhang, Dean J. Tantillo, Mingji Dai

**Affiliations:** † Department of Chemistry, Emory University, Atlanta, Georgia 30322, United States; ‡ Department of Chemistry, University of California—Davis, Davis, California 95616, United States; § Department of Pharmacology and Chemical Biology, Emory University, Atlanta, Georgia 30322, United States

## Abstract

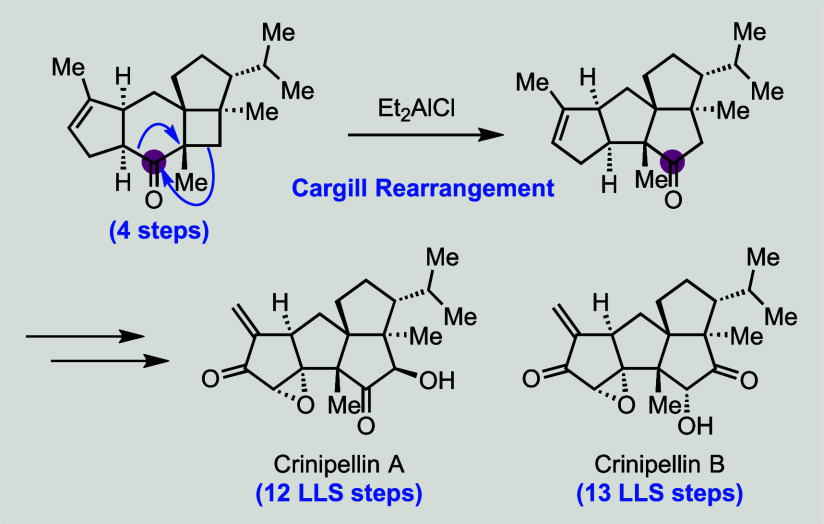

Herein, we report concise total syntheses of diterpene natural products (−)-crinipellins A and B with a tetraquinane skeleton, three adjacent all-carbon quaternary centers, and multiple oxygenated and labile functional groups. Our synthesis features a convergent Kozikowski β-alkylation to unite two readily available building blocks with all the required carbon atoms, an intramolecular photochemical [2 + 2] cycloaddition to install three challenging and adjacent all-carbon quaternary centers and a 5–6–4–5 tetracyclic skeleton, and a controlled Cargill rearrangement to rearrange the 5–6–4–5 tetracyclic skeleton to the desired tetraquinane skeleton. These strategically enabling transformations allowed us to complete total syntheses of (−)-crinipellins A and B in 12 and 13 steps, respectively. The results of quantum chemical computations revealed that the Bronsted acid-catalyzed Cargill rearrangements likely involve stepwise paths to products and the AlR_3_-catalyzed Cargill rearrangements likely involve a concerted path with asynchronous alkyl shifting events to form the desired product.

Crinipellins A (**1**), B (**2**), and related natural congeners (cf. **3**-**7**) belong to the polyquinane diterpene natural products (Scheme 1A).^1^ Crinipellins A and B were isolated by Steglich and co-workers from the fungus *Crinipellis stipitaria* (Agaricales).^2^ Since then, many other crinipellins were discovered.^3^ Structurally, the crinipellins feature a tetracyclic carbon skeleton with both a linear *cis,anti,cis*-triquinane (ABC rings) and an angular triquinane (BCD rings). Three adjacent all-carbon quaternary centers (C7, C10, and C11), eight stereogenic centers (for **1** and **2**), and multiple oxygenated functional groups are embedded in their already highly congested tetracyclic ring system. In addition, the α-methylene ketone and the α,β-epoxide located in the A ring and the α-hydroxy ketone in the C ring make crinipellins A and B labile and sensitive to various conditions. The biosynthetic pathway toward the crinipellins starts from geranylgeranyl pyrophosphate (GGPP, **8**, Scheme 1B) via a series of cationic cyclizations (**8** → **13**, cyclase phase) to build their tetracyclic ring system followed by subsequent oxidase phase to decorate the core skeleton.^4^ Biologically, crinipellins A and B have demonstrated a broad spectrum of activities including antibacterial, anticancer, and fibrinolytic activities.^5^

**Scheme 1:**
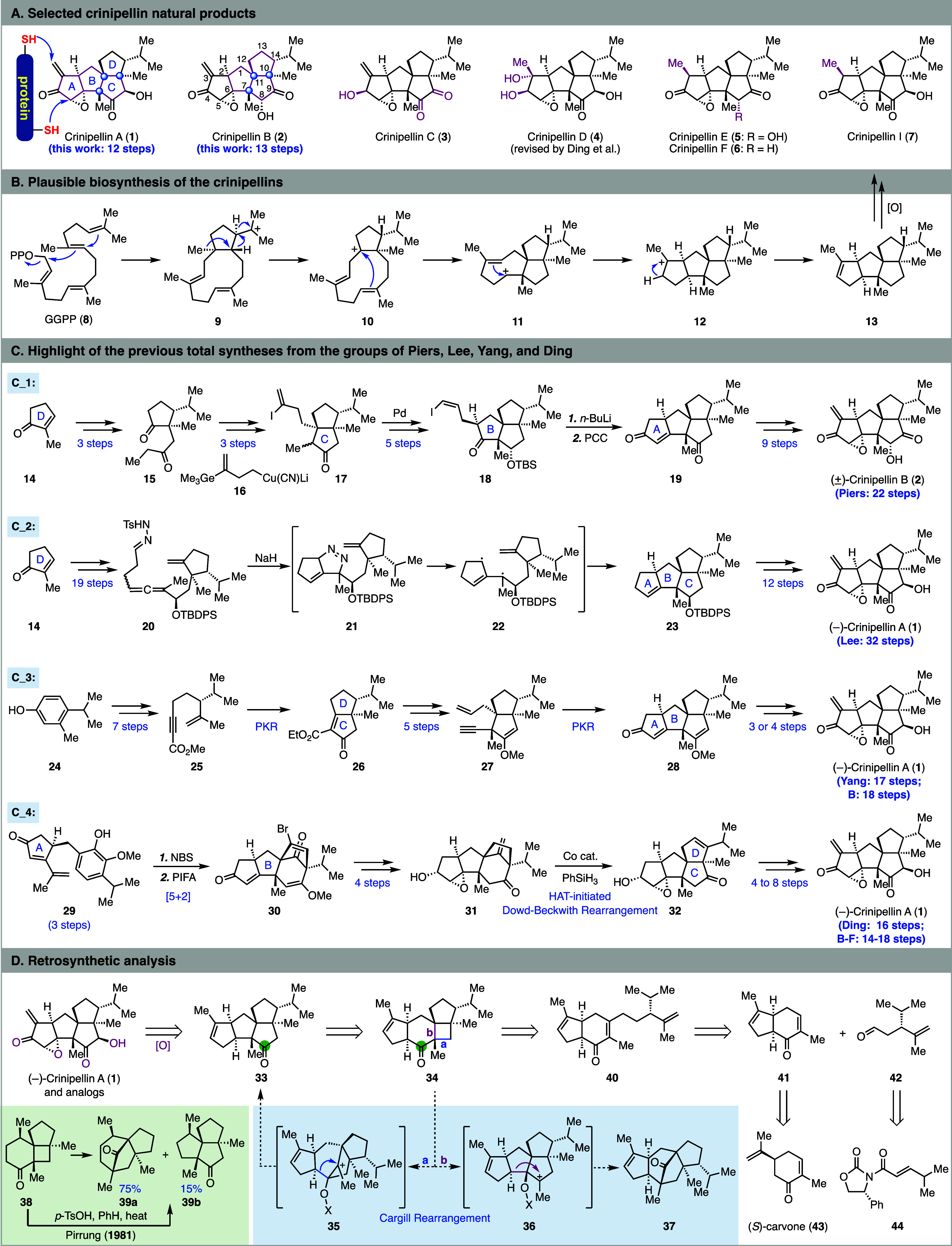
Structure, Plausible Biosynthesis, Prior Total Syntheses and Retrosynthetic Analysis of the Crinipellins

The crinipellins have attracted plenty of synthetic attention due to their delicate and complex structures and promising biological activity (Scheme 1C).^6^ So far, four elegant total syntheses have been reported. In 1993, Piers and Renaud reported the first total synthesis of (±)-crinipellin B in 22 steps.^7^ Their synthesis started from 2-methylcyclopentenone **14** (D ring) and elegantly utilized a series of carbonyl chemistries to build the ABC ring system. In 2014, Lee and co-workers reported their total synthesis of (−)-crinipellin A in 32 steps from **14**.^8^ The key step is a remarkable tandem sequence of [3 + 2] cycloaddition, nitrogen extrusion, and radical cyclization (**20** → **23**) to build the BC ring system. In 2018, Yang and co-workers disclosed their total syntheses of (−)-crinipellins A (17 steps) and B (18 steps).^9^ Their synthesis used aromatic compound **24** as a starting material and features two Pauson–Khand reactions to build the CD (**25** → **26**) and AB (**27** → **28**) ring systems consecutively. In 2022, Ding and co-workers reported a divergent approach to access seven crinipellin congeners (14–18 steps) including crinipellins A (16 steps) and B (16 steps).^10^ Their synthesis features an oxidative dearomatization-induced [5 + 2] cycloaddition to access **30**, which was later rearranged to **32** with the crinipellin carbon skeleton via a hydrogen atom transfer initiated structural rearrangement (**31** → **32**).

The α-methylene ketone and α,β-epoxide moieties of crinipellins A and B render both of them potential protein covalent modifiers.^11^ With two electrophilic sites on the A ring, they may even serve as a bivalent lock to react on two different nucleophilic sites, such as cysteines of the same yet-to-be-discovered protein target. The resurgence of covalent inhibition^12^ and our continued interest in this area^13^ promoted us to embark on the total syntheses of crinipellins A and B to support follow-up biological evaluations including target identification.

Retrosynthetically, **33** was proposed as an advanced intermediate, which could be further oxidized to the crinipellins (Scheme 1D). We envisioned that **33** with the tetraquinane core could be derived from **34** with a 5–6–4–5 tetracyclic skeleton. To realize this transformation, a cut-and-insert skeletal editing^14^ process is required to cut out the carbonyl group in the cyclohexanone and insert it into the cyclobutane ring. Specifically, we proposed a Cargill rearrangement^15^ to convert **34** to **33**. Mechanistically, our hope was that during the acid-promoted Cargill rearrangement, bond a could migrate first to form **35** with a bridged ring system, which would further rearrange to **33**. On the other hand, bond b could migrate to give **36** with a tetracyclic and fused ring system, which would then rearrange to **37** with a bridged ring system. In most of the reported Cargill rearrangements, the four-membered ring is either a cyclobutene and/or in a propellane ring system, and the stereoelectronic effect and reaction conditions are important for controlling the selectivity.^15^ In our case with a cyclobutane fused with both a six-membered ring and a five-membered ring, there is no obvious tendency of which bond (a or b) would migrate first. In a related example reported by Pirrung (**38** → **39**),^16^ under acidic (*p*-TsOH) conditions, **38** did rearrange but gave the undesired bridged product **39a** as major (75%) and the desired angular triquinane product **39b** as minor (15%). At the planning stage, how the rest of the ring system and substituents in **34** would affect the rearrangement was not clear, but if a set of complementary conditions to obtain either product could be developed and understood, it would expand the application of the Cargill rearrangement. This rearrangement strategy would allow us to use **34** as a key intermediate, which could be accessed from **40** with an intramolecular photochemical [2 + 2] cycloaddition, a reliable method to generate adjacent all-carbon quaternary centers.^17^ To assemble **40** efficiently, we proposed a formal β-alkylation of **41** with aldehyde **42** by using the method developed by Kozikowski.^18^ Compound **41** could be traced back to chiral pool molecule (*S*)-carvone (**43**)^19^ and compound **42** could be synthesized from **44** via an asymmetric conjugate addition and amide reduction.^20^

Our synthesis started from (*S*)-carvone (**43**, Scheme 2). Its six-membered ring would serve as the expanded B-ring of the crinipellins, which later needs to be contracted to the corresponding five-membered ring. Selective α-allylation of **43** gave **45** in 79% yield. Subsequent ring closing metathesis forged the five-membered A ring. The *trans* ring junction was epimerized to *cis* with a one-pot DBU treatment to yield **41** in 85% yield. Aldehyde **42** was prepared from 4-phenyl-2-oxazolidinone derivative **44**. Copper-mediated asymmetric conjugate addition gave **46** in 86% yield. The auxiliary was removed via DIBAL-H reduction to afford **42** in 84% yield. With **41** and **42** in hand, we investigated Kozikowski’s formal β-alkylation protocol to synthesize **40** with all the required carbon atoms. Enone **41** was first treated with PPh_3_ and TBSOTf to form phosphonium intermediate **47** with a TBS enol ether. LDA was then added to form the corresponding ylide for the subsequent Wittig olefination with **42** to form **48** as a 6.2/1 mixture of *E*/*Z* isomers. The extended TBS enol ether was then hydrolyzed with further addition of HF·pyridine to the same reaction mixture. Overall, the one-pot Kozikowski protocol delivered **40** in 56% yield. Enone **40** was then subjected to the [2 + 2] cycloaddition via irradiation with a 370 nm lamp in cyclohexane at 80 °C. The [2 + 2] cycloaddition efficiently built three adjacent all-carbon quaternary centers and gave **34** as a single diastereomer in 91% yield. The existing 5,6-*cis* ring junction controlled the facial selectivity by allowing the terminal olefin to approach the enone from the less hindered convex face.

**Scheme 2:**
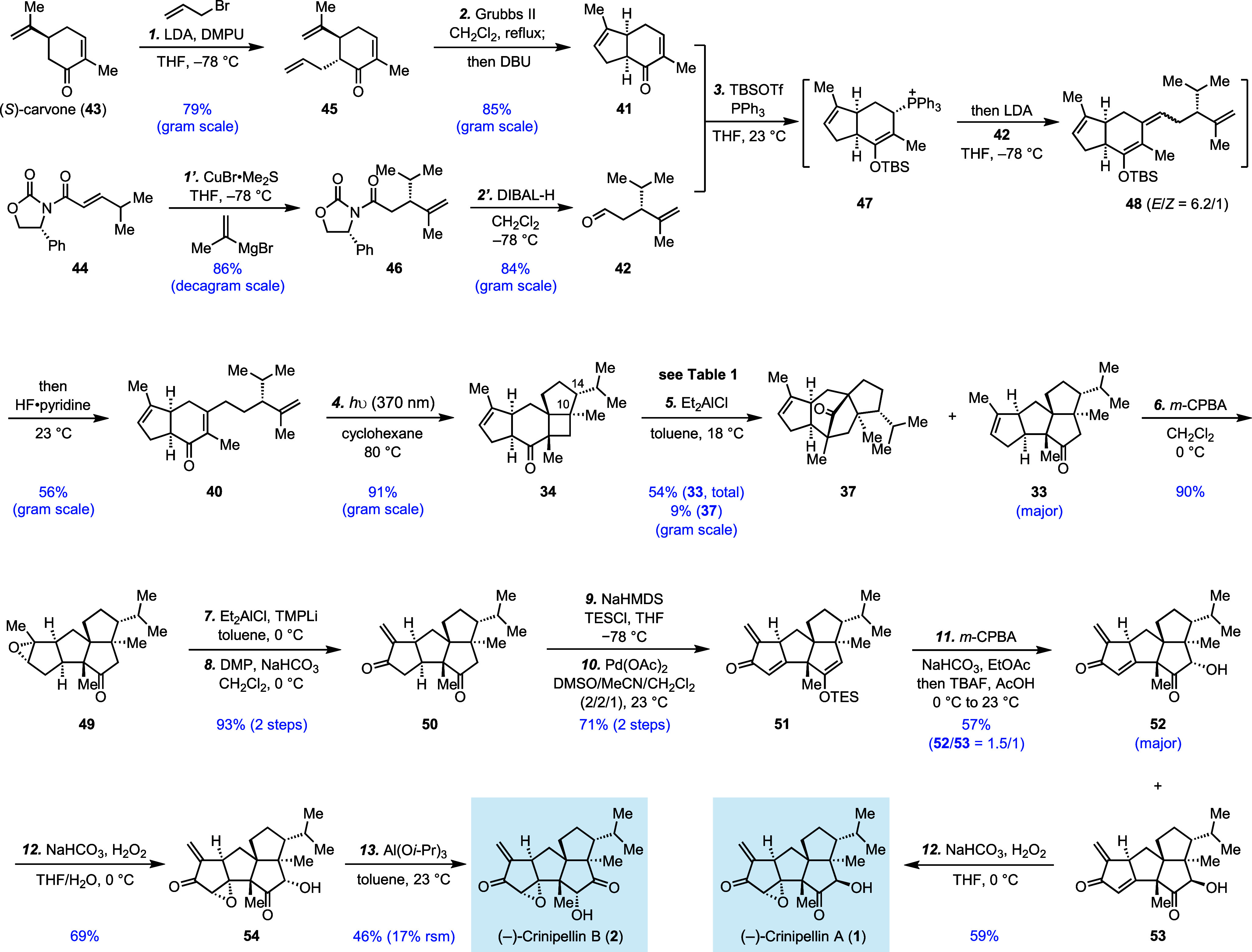
Total Syntheses of (*−*)-Crinipellins A and B

With **34** in hand, we started to investigate the key Cargill rearrangement (Table 1 and the Supporting Information). We first evaluated the *p*-TsOH conditions used by Pirrung (entry 1). While bridged product **37** was produced as the major product (45%), we were encouraged to see the formation of desired product **33** in 18% yield. Interestingly, adding LiCl inhibited both rearrangements, and **34** was recovered in 85% yield (entry 2). The use of Tf_2_NH gave **37** as the dominant product (51%) with 9% **33** (entry 3). We then switched to various Lewis acids. While Mg(ClO_4_)_2_ was not effective to promote the rearrangement, ZnCl_2_, ZnBr_2_, InCl_3_, BF_3_·Et_2_O, and AlCl_3_, all produced **37** as the major or only rearranged product (entries 4–9). When we switched from AlCl_3_ to the less Lewis acidic Me_2_AlCl and EtAlCl_2_, the yield and ratio of desired product **33** increased significantly (entries 10–13). The use of Et_2_AlCl further increased the yield of **33**, which also started to become the major product (entries 14–17). Adding LiCl could slightly increase the selectivity, but reduced the overall yield slightly at the same time (entries 15 and 17). Notably, the reaction can be conducted on a gram scale to deliver the desired product **34** in modest yield. When the reaction was conducted on a gram scale, **34a**, **34b**, and **33a** were the other isolable and identifiable byproducts. **33a** could be oxidized back to **33** with DMP to boost the overall yield of **33** from 43% to 54% (entry 18).

**Table 1:**
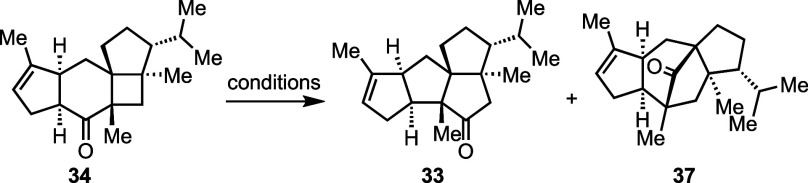

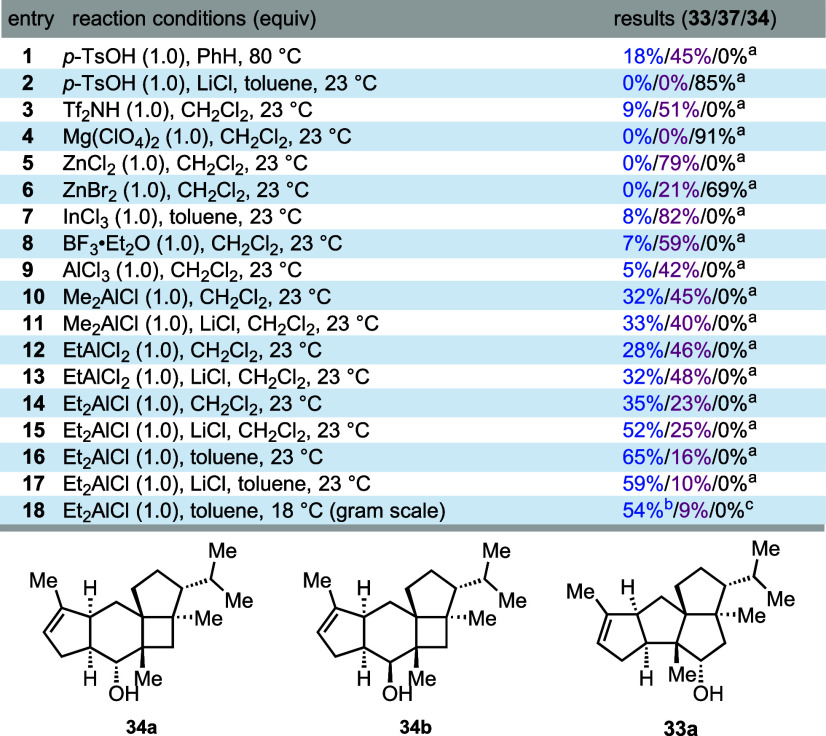
Cargill Rearrangement Optimization

a Yield was determined by NMR analysis;

b 43% isolated yield plus 11% from DMP oxidation of **33a**.

c ∼15% of **34a** and **34b**.

To complete the total synthesis (Scheme 2), Cargill rearrangement product **33** was first converted to epoxide **49** in 90% yield via a convex face *m*-CPBA epoxidation. Subsequent epoxide ring opening followed by DMP oxidation gave α-methylene ketone **50** in 93% yield over two steps. Both carbonyl groups of **50** were then converted to the corresponding TES enol ethers. Only the one in the A ring underwent Saegusa–Ito oxidation with Pd(OAc)_2_, and the other one remained intact because it was guarded by two all-carbon quaternary centers.^21^ Product **51** was obtained in 71% yield over two steps. Rubottom oxidation was next used to introduce the α-hydroxy ketone moiety in the C ring, and a 1.5/1 mixture of **52** and **53** was produced in 57% yield. Selective nucleophilic epoxidation of the more stained enone in the A ring of **53** completed a 12-step total synthesis of (−)-crinipellin A from (*S*)-carvone. For (−)-crinipellin B, after nucleophilic epoxidation of **52**, an additional step was used to isomerize the α-hydroxy ketone in the C ring and produce (−)-crinipellin B in 13 steps.

To provide further insights into the mechanisms of the Cargill rearrangements, DFT calculations (SMD(toluene)-mPW1PW91/6-31+G(d,p)) were employed.^22^ Three systems were examined in detail (Figure 1): **34**-H^+^ (akin to entry 1 in Table 1), **34**-Al(Me)Cl_2_ (akin to entry 12), and **34**-Al(Cl)Me_2_ (akin to entry 10 and entry 14). All reactions were predicted to be exergonic and effectively irreversible, and all involved epoxonium ions as intermediates en route to **37**^**+**^-X. For **34**^**+**^-H, 2-step paths were found for formation of both **33**^**+**^-H and **37**^**+**^-H, making both of these reactions stepwise dyotropic rearrangements.^23^ Overall predicted free energy barriers differed by about 3 kcal/mol, with the formation of **37** favored, as observed experimentally. For **34**^**+**^-Al(Me)Cl_2_, formation of **37**^**+**^-Al(Me)Cl_2_ was predicted to involve an epoxonium intermediate, but formation of **33**^**+**^-Al(Me)Cl_2_ was predicted not to involve an intermediate, making this reaction a concerted dyotropic reaction (albeit involving asynchronous alkyl shifting events).^22c,23^ This difference from the Bronsted acid case may result from the increased donor ability of the oxygen, making it more likely to push the alkyl group to migrate to form **33**^**+**^-X. The predicted overall free energy barriers for formation of both products were similar (although favoring formation of **37**^**+**^-Al(Me)Cl_2_), and both products were observed in comparable amounts experimentally (although CH_2_Cl_2_ rather than toluene was used). For **34**^**+**^-Al(Cl)Me_2_, the formation of **33**^**+**^-Al(Cl)Me_2_ was predicted to be favored by 1 kcal/mol over the formation of **37**^**+**^-Al(Cl)Me_2_. The predicted selectivity trend is in the same direction as that experimentally observed, but experimentally, **37** is slightly favored with Me_2_AlCl (entry 10) and **33** is slightly favored with Et_2_AlCl (entry 14). Et_2_AlCl works better than Me_2_AlCl presumably because of the further increased donor ability of the oxygen with Et_2_AlCl to push the alkyl group migration to form **33**. While all of these results are in line with experiment, it is important to note that the observed selectivities correspond to small *ΔΔG*^*‡*^s, which fall within the expected error bars for DFT methods, such as the one used. That being said, our conclusions about intermediates are not expected to be sensitive to the level of theory used.

**Figure 1:**
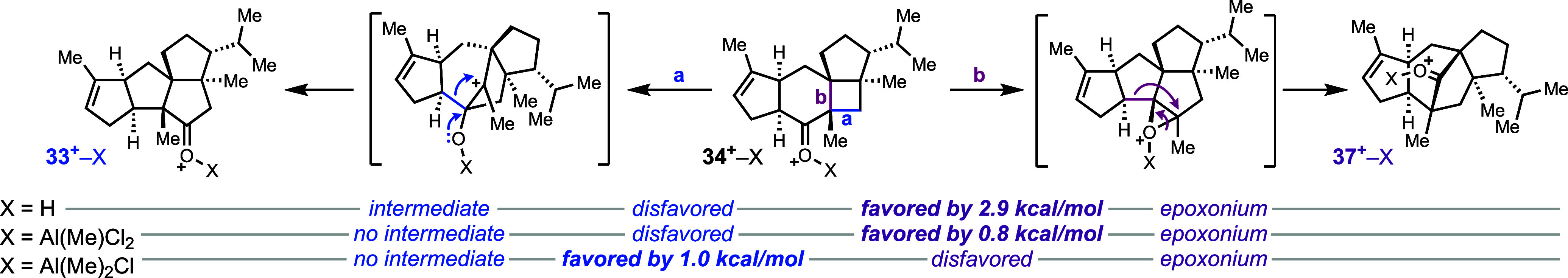
Computational results on mechanisms of the Cargill rearrangements.

In summary, starting from cheap and abundant chiral pool molecule (*S*)-carvone, we completed total syntheses of (−)-crinipellins A and B in 12 and 13 steps, respectively. The key steps include a Kozikowski formal β-alkylation to bring together two readily available building blocks **41** and **42**, an intramolecular photochemical [2 + 2] cycloaddition to install three challenging and adjacent all-carbon quaternary centers and a 5–6–4–5 tetracyclic skeleton, and a Cargill rearrangement to convert the 5–6–4–5 tetracyclic skeleton to the desired tetraquinane skeleton. The Cargill rearrangement is strategically important and allowed us to use the six-membered (*S*)-carvone as the B ring precursor, from which the A ring was installed via ring closing metathesis and the C ring precursor together with the D ring were constructed via the [2 + 2]-cycloaddition. Notably, a set of conditions were developed to get either the bridged or fused product via the Cargill rearrangement. Computational studies indicated that both stepwise and concerted mechanisms are possible for these rearrangements, with unexpected epoxonium intervening in the formation of **37**.

## Data Availability

In addition to the Supporting Information, computed structures are available through the ioChem-BD repository at 10.19061/iochem-bd-6-349.

## Supporting Information Available

Experimental procedures and NMR spectra for all new compounds (PDF)

## Abbreviations

PKR: Pauson–Khand Reaction
NBS: *N*-bromosuccinimide
PIFA: phenyliodine bis(trifluoroacetate)
LDA: lithium diisopropylamide
DMPU: *N*,*N′*-dimethylpropyleneurea
DBU: 1,8-diazabicyclo(5,4,0)undec-7-ene
*m*-CPBA: *meta*-chloroperoxybenzoic acid
TMPLi: lithium 2,2,6,6-tetramethylpiperidide
DMP: Dess-Martin periodinane
DFT: density functional theory
