# Supplementary material for: Concise Total Syntheses of (−)-Crinipellins A and B Enabled by a Controlled Cargill Rearrangement
Source: J Am Chem Soc. 2024 Jul 25;146(31):21250–6. doi: 10.1021/jacs.4c07900 (PMC11311239; doi:10.1021/jacs.4c07900)
Supplement: Supplementary file 1 — ja4c07900_si_001.pdf [file ja4c07900_si_001.pdf]

Supporting Information

Concise Total Syntheses of (−)-Crinipellins A and B Enabled by a Controlled Cargill Rearrangement

Bo Xu,[1] Ziyao Zhang,[1] Dean J. Tantillo,*[,2] and Mingji Dai*[,1,3]

[1]Department of Chemistry, Emory University, Atlanta, Georgia 30322, United States;

[2]Department of Chemistry, University of California–Davis, Davis, California 95616, United States;

[3]Department of Pharmacology and Chemical Biology, School of Medicine, Emory University, Atlanta, Georgia 30322, United States.

Table of Content

A. General Methods. 3

B. Experiment Procedure for the Total Synthesis of Cripnipellin A and B 4

C. NMR Comparison of Our Synthetic Samples and Previous Reports 20

D. References 28

E. ^{1}H and ^{13}C NMR spectra 29

A. General Methods. All commercially available compounds were purchased from Sigma-Aldrich, Alfa-Aesar, Oakwood chemicals and Ambeed unless otherwise noted. Materials obtained from commercial suppliers were used without further purification. NMR spectra were recorded on Bruker spectrometers (^{1}H at 400 MHz, 600 MHz, 800 MHz and ^{13}C at 100 MHz, 125 MHz, 200 MHz). Chemical shifts (δ) were given in ppm with reference to solvent signals [^{1}H NMR: $CHCl_3$ (7.26); ^{13}C NMR: $CDCl_3$ (77.00)]. ^{1}H NMR data are reported as follows: chemical shift (δ ppm), multiplicity (s = singlet, d = doublet, t = triplet, q = quartet, m = multiplet, br = broad), coupling constant (Hz), and integration. Column chromatography was performed on silica gel. All reactions sensitive to air or moisture were conducted under argon atmosphere in dry solvents under anhydrous conditions, unless otherwise noted. Dry THF and DCM (CH_2Cl_2) were processed via PureProcessTechnology GS-SPS-5-CM system. Dry Et_2O and toluene were purchased from Sigma-Aldrich. All other solvents and reagents were used as obtained from commercial sources without further purification. Room temperature (r.t.) is around 23 °C.

B. Experiment Procedure for the Total Synthesis of Cripnipellin A and B

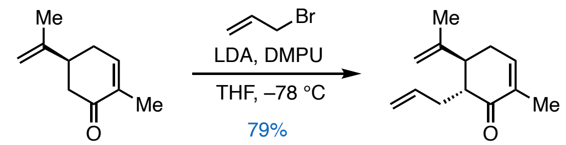

(*S*)-carvone (**43**) **45**

(*S*)-carvone (**43**) (1.0 equiv, 6 g, 40 mmol) was added to a 100 mL flame-dried round bottom-flask under argon atmosphere. Anhydrous THF (200 mL, 0.2 M) was added and the solution was cooled to –78 °C. Lithium diisopropylamide solution (1.2 equiv, 2.0 M in THF/heptane/ethylbenzene, 24 mL, 48 mmol) was added slowly. The solution was stirred for additional 1 h, and anhydrous DMPU (4.0 equiv, 19.35 mL, 160 mmol) was added. Then allyl bromide (1.5 equiv, 5.23 mL, 60 mmol) was added. The mixture was allowed to slowly warmed up to –20°C (dry ice/acetone bath, about 8 h). The reaction was quenched with saturated ammonium chloride and extracted with hexanes. The combined organic layers were washed with brine, dried over Na_2SO_4, and concentrated under reduced pressure. The crude product was subjected to flash chromatography (hexanes to hexanes : EtOAc = 30 : 1) to yield enone **45** (6.02 g, 79%) as a pale yellow oil.

$[\alpha]_D^{23}$ = –43.2 (c = 0.4, $CHCl_3$).

IR (neat): 3074, 2976, 2919, 2851, 1667, 1432,1377, 1366, 1231, 1189, 1167, 1136, 1072.

^{1}H NMR (400 MHz, $CDCl_3$): δ 6.65 (ddt, J = 5.5, 3.0, 1.5 Hz, 1H), 5.84 – 5.73 (m, 1H), 5.11 – 4.92 (m, 2H), 4.84 (p, J = 1.6 Hz, 1H), 4.79 (dt, J = 1.8, 0.8 Hz, 1H), 2.68 (ddd, J = 11.4, 9.5, 5.2 Hz, 1H), 2.53 (m, 1H), 2.47 – 2.35 (m, 2H), 2.35 – 2.18 (m, 2H), 1.76 (dt, J = 2.2, 1.5 Hz, 3H), 1.71 (dd, J = 1.6, 0.8 Hz, 3H).

^{13}C NMR (100 MHz, $CDCl_3$): δ 200.6, 145.3, 143.2, 136.0, 135.1, 116.7, 113.5, 48.8, 46.7, 31.7, 30.6, 18.9, 16.0.

HRMS m/z (APCI): calc. for $C_{13}H_{19}O^+$ $[M+H]^+$: 191.1430, found: 191.1436.

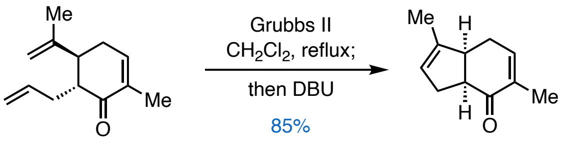

45 **41**

Grubbs second-genration catalyst (0.02 equiv, 340 mg, 0.4 mmol) was added to a 200 mL round bottom flask connected to a condenser. The flask was evacuated and backfilled with argon three times. Anhydrous DCM (100 mL) was added and the solution was heated to reflux. A solution of enone **45** (1 equiv, 3.8 g, 20 mmol) in DCM (20 mL) was added very slowly (over 4 h) to the reaction mixture. The reaction was refluxed for 12 h. DBU (2.0 equiv, 6 mL, 40 mmol) was added, and the reaction mixture was further stirred

for 12 h, and then cooled to room temperature. The solvent was removed under reduced pressure. The residue was cooled to 0 °C before 1.0 M HCl (42 mL) was added dropwise. The mixture was stirred for about 10 min, and then extracted with pentane. The combined organic layers were washed with brine, dried over Na_2SO_4, and concentrated under reduced pressure. The crude product was subjected to flash chromatography (hexanes to hexanes : EtOAc = 20 : 1) to yield **41** (2.75 g, 85%) as a colorless oil.

$[\alpha]_D^{21}$ = 190.9 (c = 1.0, $CHCl_3$).

IR (neat): 3041, 2951, 2921, 2848, 1659, 1449, 1432, 1378, 1364, 1272, 1203, 1185, 1103, 1075, 1059.

^{1}H NMR (400 MHz, $CDCl_3$): δ 6.64 – 6.52 (m, 1H), 5.43 – 5.32 (m, 1H), 3.05 – 2.95 (m, 1H), 2.91 (q, J = 6.8 Hz, 1H), 2.61 – 2.48 (m, 3H), 2.35 – 2.24 (m, 1H), 1.77 (q, J = 1.8 Hz, 3H), 1.68 – 1.66 (m, 3H).

^{13}C NMR (101 MHz, $CDCl_3$): δ 201.8, 142.3, 142.2, 136.1, 124.8, 48.7, 45.2, 35.4, 25.6, 16.4, 14.8.

HRMS m/z (APCI): calc. for $C_{11}H_{15}O^+$ $[M+H]^+$: 163.1117, found: 163.1123.

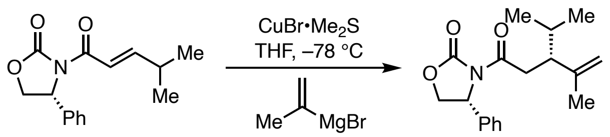

CuBr•DMS (1.3 equiv, 40.7 g, 0.2 mol) was added to a 2 L flame-dried three necked round-bottom flask. The flask was evacuated and backfilled with argon three times. Anhydrous THF (1.6 L, ca. 0.1 M) was added to the flask, and the suspension was cooled to –78 °C. Isopropenylmagnesium bromide (1.3 equiv, 0.5 M in THF, 400 mL, 0.2 mol) was added dropwise over 8 h via a syringe pump. The off-white suspension gradually turned into dark red, and the mixture was stirred for another 2 h at the same temperature. Amide **44**[1] (1.0 equiv, 40 g, 0.15 mol) in 230 mL THF was added dropwise over 20 h at –78 °C. The reaction was stirred for another 1 h. After full conversion of amide **44**, the reaction was quenched with saturated ammonium chloride and extracted with EtOAc. The combined organic layers were washed with brine, dried over Na_2SO_4, and concentrated under reduced pressure. The crude product was recrystallized in pure pentane to afford amide **46** (40.7 g, 86%) as a white solid.

m.p. 69 °C – 70 °C.

$[\alpha]_D^{23}$ = –104.8 (c = 1.0, $CHCl_3$).

IR (neat): 3074, 3034, 2965, 2933, 2898, 2873, 1777, 1700, 1605, 1493, 1455, 1382, 1355, 1335, 1310, 1265, 1209, 1194, 1178, 1136, 1097, 1068, 1052.

^{1}H NMR (400 MHz, $CDCl_3$): δ 7.42 – 7.16 (m, 5H), 5.38 (dd, J = 8.6, 3.6 Hz, 1H), 4.72 – 4.55 (m, 3H), 4.24 (dd, J = 8.9, 3.6 Hz, 1H), 3.16 – 3.00 (m, 2H), 2.35 – 2.29 (m, 1H), 1.72 – 1.54 (m, 4H), 0.92 (d, J = 6.7 Hz, 3H), 0.84 (d, J = 6.7 Hz, 3H).

^{13}C NMR (100 MHz, CDCl$_3$): δ 172.2, 153.6, 146.3, 139.0, 129.0, 128.5, 125.8, 112.3, 69.8, 57.7, 50.2, 36.9, 30.2, 21.0, 20.02, 20.00.

HRMS m/z (APCI): calc. for $C_{18}H_{24}NO_3^+$ [M+H]$^+$: 302.1751 found:302.1756

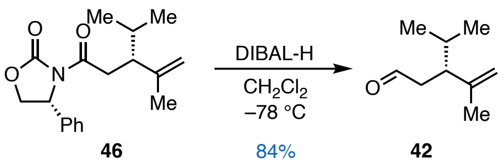

Amide **46** (1.0 equiv, 4.5 g, 15 mmol) was added to a 100 mL flame-dried round bottom-flask under argon atmosphere. Anhydrous DCM (50 mL, 0.3 M) was added and the solution was cooled to –78 °C. DIBAL-H (2.0 equiv, 1.0 M in toluene, 30 mL, 30 mmol) was added very slowly (over 30 min) into the flask by "dribbling" down the inside wall of the flask. The solution was further stirred at the same temperature for 1 h. After full conversion of amide **46**, the reaction was quenched with saturated Rochelle salt solution and extracted with diethyl ether. The combined organic layers were washed with brine and dried over Na_2SO_4. The solvent was removed by fraction distillation. Pentane (50 mL) was added and fraction distillation was done again to remove the remaining ether. The pentane solution of crude product was directly subjected to column chromatography (Pentane : Ether = 5 : 1) to yield aldehyde **42** (1.8 g, 84%) as a colorless oil.

$[\alpha]_D^{23}$ = –52.9 (c = 0.1, CHCl$_3$).

IR (neat): 3074, 2960, 2873, 2817, 2719, 1724, 1645, 1469, 1450, 1414, 1487, 1377, 1369, 1277, 1229, 1217, 1166, 1110, 1058.

^{1}H NMR (400 MHz, CDCl$_3$): δ 9.61 (dd, J = 3.4, 1.7 Hz, 1H), 4.81 (p, J = 1.6 Hz, 1H), 4.72 (dt, J = 1.8, 0.8 Hz, 1H), 2.57 – 2.44 (m, 1H), 2.42 – 2.27 (m, 2H), 1.74 – 1.59 (m, 4H), 0.87 (dd, J = 6.7, 5.2 Hz, 6H).

^{13}C NMR (100 MHz, CDCl$_3$): δ 203.0, 145.7, 113.0, 48.8, 44.4, 29.7, 20.9, 19.9, 19.6.

HRMS m/z (APCI): calc. for $C_9H_{17}O^+$ [M+H]$^+$: 141.1274, found: 141.1276.

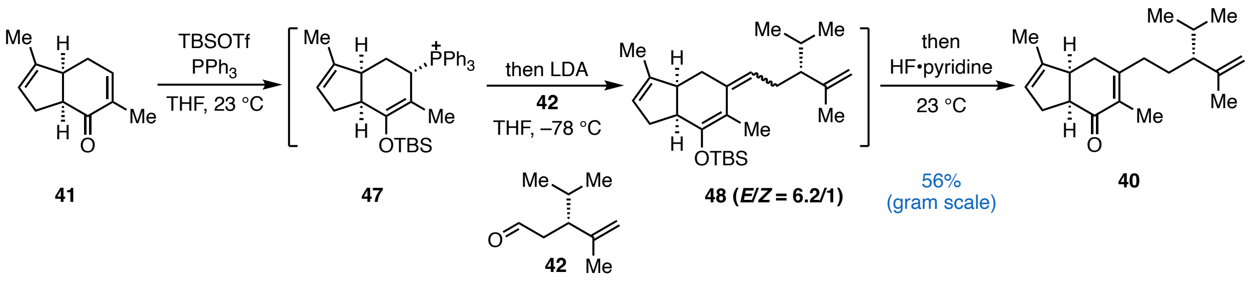

A flame-dried 100 mL round-bottom flask charged with PPh$_3$ (1.5 equiv, 3.39 g, 12.9 mmol) was evacuated and backfilled with argon three times. Anhydrous THF (40 mL, 0.5 M) and TBSOTf (1.5 equiv, 3.42 g, 12.9 mmol) was added sequentially. Enone **41** (1.0 equiv, 1.4 g, 8.6 mmol) was added dropwise, and the

solution was stirred at room temperature for 1 h. The colorless solution gradually turned into a yellow solution. After TLC showed full consumption of enone **41**, the mixture was cooled to –78 °C and LDA (1.5 equiv, 2.0 M in THF/heptane/ethylbenzene, 6.45 mL, 12.9 mmol) was added dropwise. The yellow solution quickly turned dark red. After stirring for 15 min, aldehyde **42** (1.5 equiv, 1.8 g, 12.9 mmol) was added dropwise. The reaction mixture was quickly raised to room temperature and stirred for another 2 h. HF•pyridine (3.0 equiv, 70% HF, 0.7 mL, 25.8 mmol) was added to the mixture. The mixture was stirred for additional 30 min, quenched with water, and extracted with hexanes. The combined organic layers were washed with brine and condensed under reduced pressure. The crude product was subjected to flash chromatography (hexanes : EtOAc = 50 : 1) to yield enone **40** (1.4 g, 56%) as a colorless oil.

$[\alpha]_D^{22}$ = 61.5 (c = 1.0, $CHCl_3$).

IR (thin film): 3074, 2969, 2954, 2926, 2869, 1738, 1655, 1447, 1376, 1365, 1294, 1229, 1217, 1205, 1166, 1086, 1060.

^{1}H NMR (400 MHz, $CDCl_3$): δ 5.39 – 5.37 (m, 1H), 4.85 – 4.83 (m, 1H), 4.69 (dt, J = 2.5, 0.7 Hz, 1H), 2.97 – 2.83 (m, 2H), 2.65 – 2.46 (m, 3H), 2.29 – 2.22 (m, 1H), 2.19 – 2.12 (m, 1H), 1.98 (td, J = 12.3, 5.0 Hz, 1H), 1.74 (t, J = 1.8 Hz, 3H), 1.69 – 1.67 (m, 3H), 1.67 – 1.64 (m, 1H), 1.63 (dd, J = 1.4, 0.8 Hz, 3H), 1.59 (s, 1H), 1.57 – 1.48 (m, 1H), 1.35 – 1.24 (m, 1H), 0.92 (d, J = 6.5 Hz, 3H), 0.83 (d, J = 6.6 Hz, 3H).

^{13}C NMR (100 MHz, $CDCl_3$): δ 201.8, 155.8, 146.1, 142.3, 131.0, 124.8, 112.9, 55.3, 47.6, 44.3, 35.6, 34.4, 30.6, 30.1, 27.7, 21.5, 20.7, 18.8, 14.9, 10.9.

HRMS m/z (APCI): calc. for $C_{20}H_{31}O^+$ $[M+H]^+$: 287.2369, found: 287.2377.

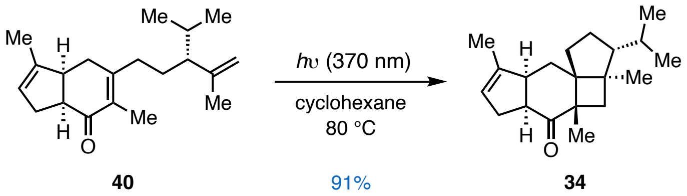

Enone **40** (1.0 equiv, 1.4 g, 4.7 mmol) was added to a 1 L round-bottom flask. Cyclohexane (470 mL, 0.01 M) was added. The solution was sparged with argon for 30 min. A condenser was then attached to the flask, and the system was evacuated and backfilled with argon ten times. The solution was heated to reflux and then irradiated with 370 nm lamp for 8 h. Upon full conversion of enone **40**, the solution was cooled to room temperature, and concentrated under reduced pressure. The crude product was directly subjected to flash chromatography to yield ketone **35** (1.3 g, 91%) as a colorless oil.

$[\alpha]_D^{21}$ = 83.5 (c = 1.0, $CHCl_3$).

IR (thin film): 3074, 2952, 2930, 2867, 1690, 1469, 1440, 1377, 1364, 1316, 1303, 1280, 1260, 1228, 1200, 1170, 1114, 1111, 1098.

^{1}H NMR (400 MHz, CDCl$_3$): δ 5.30 – 5.23 (m, 1H), 3.47 – 3.35 (m, 1H), 3.35 – 3.24 (m, 1H), 2.64 – 2.54 (m, 2H), 2.27 (d, *J* = 12.3 Hz, 1H), 2.00 (dd, *J* = 14.4, 4.8 Hz, 1H), 1.97 – 1.89 (m, 1H), 1.84 – 1.74 (m, 2H), 1.72 – 1.70 (m, 3H), 1.65 (d, *J* = 12.3 Hz, 1H), 1.62 – 1.55 (m, 1H), 1.41 – 1.31 (m, 1H), 1.27 (s, 3H), 1.25 – 1.20 (m, 1H), 1.19 – 1.12 (m, 1H), 1.10 (s, 3H), 0.89 (d, *J* = 6.5 Hz, 3H), 0.85 (d, *J* = 6.6 Hz, 3H).
^{13}C NMR (100 MHz, CDCl$_3$): δ 219.6, 142.6, 123.2, 59.5, 55.8, 48.3, 47.4, 47.3, 45.6, 43.7, 35.2, 34.6, 34.6, 29.0, 28.6, 22.4, 21.7, 21.0, 17.2, 14.9.
HRMS m/z (APCI): calc. for $C_{20}H_{31}O^{+}$ $[M+H]^{+}$: 287.2369, found: 287.2377.

Optimization of Cargill rearrangement

General procedure. Ketone **34** (1.0 equiv, 28.6 mg, 0.1 mmol) was added to a 2-10 mL Biotage microwave tube charged with a magnetic stir bar. Toluene (ca. 1 mL) was added and rotavaped to get rid of water, and this was repeated for three times. Solid reagents including LiCl and solid acids were added first, then anhydrous solvent (4 mL, 0.25 M) was added. Liquid acids or acid solutions were added after solvent addition at 0 °C. The mixture was stired for 3 days before it was directly filtered with a pad of silica. The filtrate was concentrated under reduced pressure. Trichloroethene (13.1 mg, 0.1 mmol) was added as an internal standard. The yield was determined via ^{1}H NMR.

Table S1. Condition screening of Cargill rearrangement

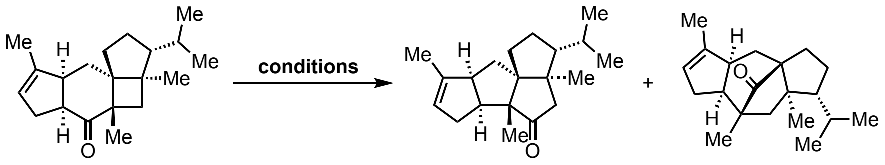

Entry	Conditions	Result
**0**	Et_2AlCl, LiCl, Toluene, Glovebox	14% (**34**) + 45% (**33**)[1] + 20% (**37**) + 20% (**34a**)[2]
**1**	Et_2AlCl, LiCl, Toluene	0% (**34**) + 59% (**33**) + 10% (**37**) +0% (**34a**)
**2**	Et_2AlCl, Toluene	0% (**34**) + 65% (**33**) + 16% (**37**) +0% (**34a**)
**3**	Et_2AlCl, LiCl, Toluene, 7 days	0% (**34**)+ 58% (**33**) + 11% (**37**) +0% (**34a**)
**4**	Et_2AlCl, LiCl, PhH	24% (**34**) + 44% (**33**) + 8% (**37**) +0% (**34a**)
**5**	Et_2AlCl, DCM	0% (**34**) + 35% (**33**) + 23% (**37**) +0% (**34a**)
**6**	Et_2AlCl, LiCl, DCM	0% (**34**) + 52% (**33**) + 25% (**37**) +0% (**34a**)
**7**	$EtAlCl_2$, DCM	0% (**34**) + 28% (**33**) + 46% (**37**) +0% (**34a**)
**8**	$EtAlCl_2$, LiCl, DCM	0% (**34**) + 32% (**33**) + 48% (**37**) +0% (**34a**)
**9**	Me_2AlCl, DCM	0% (**34**) + 32% (**33**) + 45% (**37**) +0% (**34a**)
**10**	Me_2AlCl, LiCl, DCM	0% (**34**) + 33% (**33**) + 40% (**37**) +0% (**34a**)
**11**	Ph_2AlCl[3]	11% (**34**) + 16% (**33**) + 0% (**37**) +0% (**34a**)
**12**	Ph_2InCl[4]	77% (**34**) + 0% (**33**) + 0% (**37**) +0% (**34a**)
**13**	$AlCl_3$, DCM	0% (**34**) + 5% (**33**) + 42% (**37**) +0% (**34a**)
**14**	$InCl_3$, Toluene	0% (**34**) + 8% (**33**) + 82% (**37**) +0% (**34a**)
**15**	PTSA, Toluene, 80 °C	0% (**34**) + 29% (**33**) + 69% (**37**) +0% (**34a**)
**16**	PTSA, LiCl, Toluene	85% (**34**) + 0% (**33**) + 0% (**37**) +0% (**34a**)
**17**	PTSA, PhH, reflux	0% (**34**) + 18% (**33**) + 45% (**37**) +0% (**34a**)
**18**	PTSA, PhH	74% (**34**) + 0% (**33**) + 0% (**37**) +0% (**34a**)
**19**	BF_3•OEt_2, DCM	0% (**34**) + 7% (**33**) + 59% (**37**) +0% (**34a**)
**20**	Tf_2NH, DCM	0% (**34**) + 9% (**33**) + 51% (**37**) +0% (**34a**)
**21**	$ZnCl_2$, DCM	0% (**34**) + 0% (**33**) + 79% (**37**) +0% (**34a**)
**22**	$Mg(ClO_4)_2$, DCM	91% (**34**) + 0% (**33**) + 0% (**37**) +0% (**34a**)
**23**	$ZnBr_2$, DCM	69% (**34**) + 0% (**33**) + 21% (**37**) +0% (**34a**)
**24**	TMSOTf	0% (**34**) + 10% (**33**) + 52% (**37**) +0% (**34a**)

[1] 45% isolated yield, [2] 22% isolated yield, [3] made with $AlCl_3$ and PhLi in toluene, [4] made with $InCl_3$ and PhLi in toluene.

Scheme S1. Crude NMR for Cargill optimization

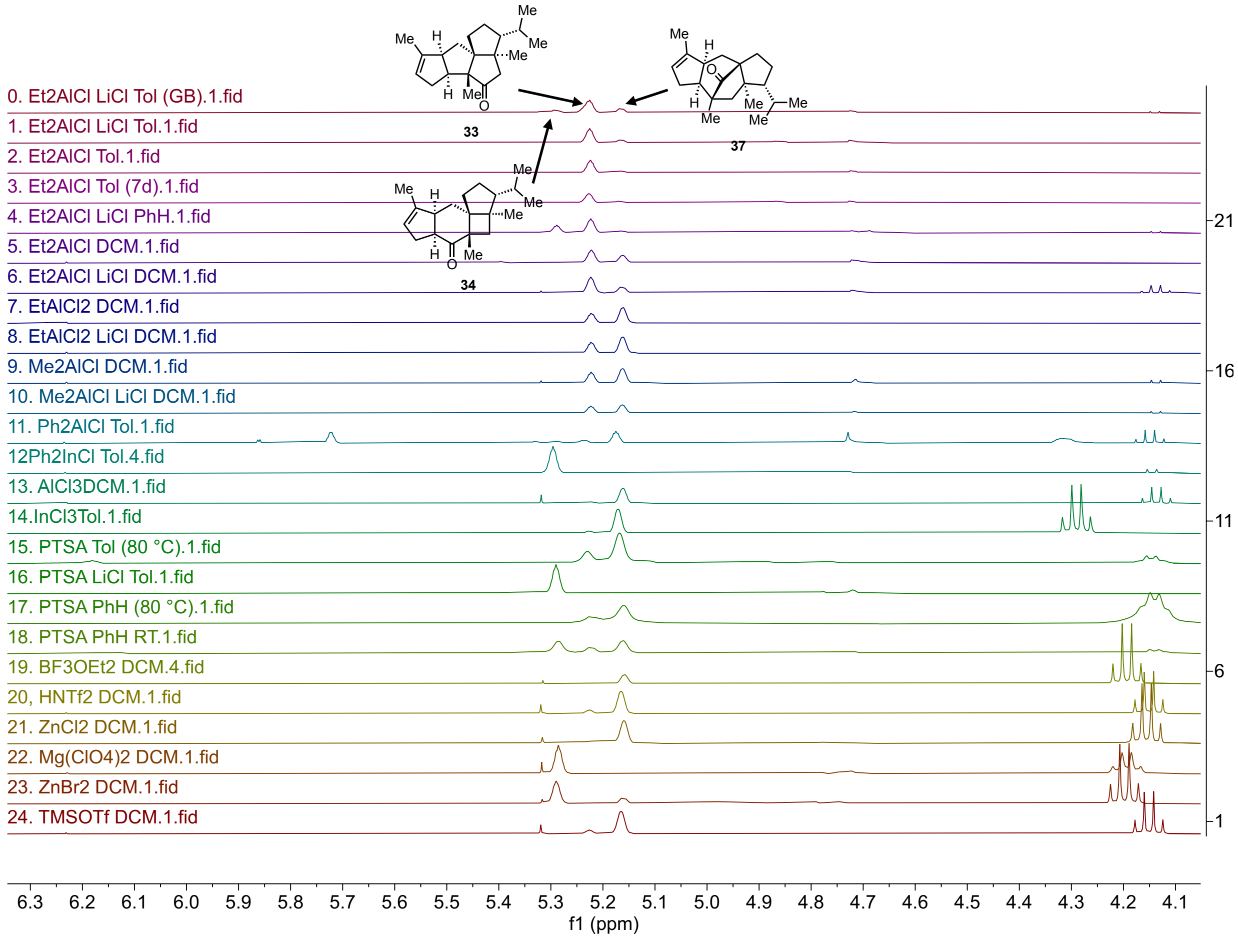

Gram scale preparation

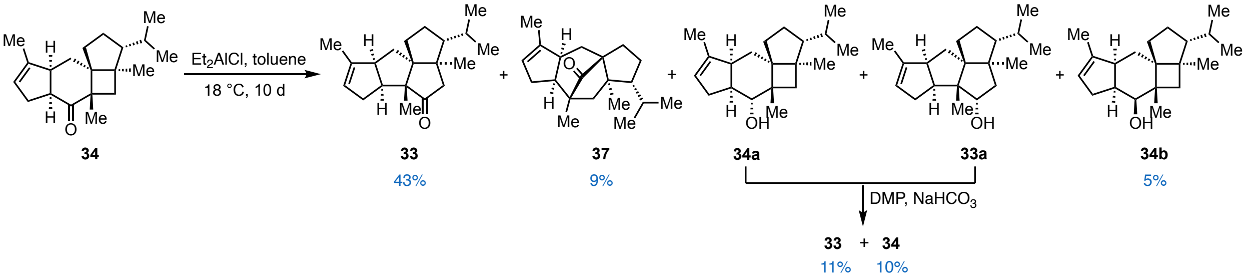

A 250 mL round-bottom flask charged with ketone **34** (1.0 equiv, 1.0 g, 3.5 mmol) was added toluene (140 mL, 0.025 M) under nitrogen atmoshphere. The solution was cooled to 0 °C, and Et_2AlCl (1.0 equiv, 1 M in hexane, 3.5 mL, 3.5 mmol) was added dropwise. The reaction was then warmed up to 18 °C and stirred for 10 days. Upon full conversion of ketone **34**, the mixture was quickly filtered through a pad of silica and concentrated under reduced pressure. The crude product was directly subjected to flash chromatography (hexane : EtOAc = 50 : 1 to hexane : EtOAc = 20 : 1) to yield **33** (426 mg, 43%) as a colorless oil together with **37** (92 mg, 9%), **34b** (50 mg, 5%), and a mixture of **34a** and **33a**.

33:

[α]$_D^{21}$ = -92.4 (c = 0.5, $CHCl_3$).

IR (thin film): 3074, 2955, 2869, 1730, 1452, 1378, 1264, 1176, 1074.

^{1}H NMR (400 MHz, $CDCl_3$): δ 5.27 – 5.09 (m, 1H), 2.90 – 2.70 (m, 2H), 2.60 (d, J = 18.8 Hz, 1H), 2.38 – 2.26 (m, 2H), 2.19 (d, J = 18.8 Hz, 1H), 1.85 (dd, J = 13.7, 7.9 Hz, 1H), 1.81 – 1.72 (m, 2H), 1.66 – 1.60 (m, 4H), 1.58 – 1.33 (m, 4H), 1.01 (s, 6H), 0.92 (d, J = 6.5 Hz, 3H), 0.86 (d, J = 6.5 Hz, 3H).

^{13}C NMR (100 MHz, $CDCl_3$): δ 225.1, 142.8, 123.7, 66.6, 61.7, 56.3, 52.9, 51.2, 49.7, 47.7, 40.5, 33.3, 31.8, 30.9, 30.3, 22.9, 22.3, 18.1, 17.7, 15.2.

HRMS m/z (APCI): calc. for $C_{20}H_{31}O^+$ $[M+H]^+$: 287.2369, found: 287.2377.

37:

[α]$_D^{21.2}$ = 73.0 (c = 0.5, $CHCl_3$).

IR (thin film): 3074, 2956, 2925, 2867, 2848, 1738, 1451, 1440, 1377, 1365, 1344, 1323, 1293, 1279, 1258, 1233, 1207, 1181, 1167,, 1136, 1118, 1100, 1076, 1054.

^{1}H NMR (600 MHz, $CDCl_3$): δ 5.13 (q, J = 2.1 Hz, 1H), 2.72 – 2.63 (m, 1H), 2.59 (m, 1H), 2.18 – 2.05 (m, 3H), 2.00 – 1.90 (m, 2H), 1.80 (d, J = 13.2 Hz, 1H), 1.72 (m, 1H), 1.65 – 1.59 (m, 3H), 1.54 – 1.48 (m, 1H), 1.41 (dd, J = 14.1, 10.9 Hz, 1H), 1.19 (ddt, J = 13.2, 11.6, 5.9 Hz, 1H), 1.08 (s, 3H), 1.02 (ddd, J = 13.5, 11.9, 3.5 Hz, 1H), 0.99 (s, 3H), 0.93 (d, J = 6.7 Hz, 3H), 0.83 – 0.77 (m, 4H).

^{13}C NMR (150 MHz, $CDCl_3$): δ 222.9, 143.9, 122.5, 62.4, 61.0, 57.4, 49.8, 48.6, 44.3, 42.6, 41.8, 32.4, 30.6, 28.2, 26.1, 22.7, 22.5, 19.4, 15.3, 14.9.

HRMS m/z (APCI): calc. for $C_{20}H_{31}O$ $[M+H]^+$: 287.2369, found: 287.2378.

34a:

[α]$_D^{23}$ = 49.3 (c = 0.5, $CHCl_3$).

IR (thin film): 3366, 3031, 2951, 2926, 2867, 1738, 1663, 1618, 1444, 1374, 1365, 1261, 1228, 1216, 1205, 1092, 1063.

^{1}H NMR (400 MHz, $CDCl_3$): δ 5.25 – 5.18 (m, 1H), 3.18 (d, J = 10.9 Hz, 1H), 3.13 – 2.99 (m, 1H), 2.70 – 2.58 (m, 1H), 2.51 – 2.43 (m, 1H), 2.36 – 2.29 (m, 1H), 2.02 (d, J = 12.2 Hz, 1H), 1.98 – 1.91 (m, 1H), 1.78 – 1.70 (m, 2H), 1.62 (qq, J = 2.8, 1.4 Hz, 5H), 1.33 (dd, J = 12.3, 2.4 Hz, 2H), 1.29 – 1.25 (m, 1H), 1.20 (s, 3H), 1.09 (s, 4H), 0.95 – 0.89 (m, 1H), 0.89 – 0.86 (m, 3H), 0.85 – 0.80 (m, 3H).

^{13}C NMR (100 MHz, $CDCl_3$): δ 143.0, 121.8, 79.0, 61.1, 52.7, 45.3, 44.5, 40.5, 39.3, 38.3, 37.5, 36.2, 33.5, 28.7, 28.5, 22.9, 22.4, 21.6, 17.4, 14.4.

HRMS m/z (APCI): calc. for $C_{20}H_{33}O$ $[M+H]^+$: 289.2526, found: 289.2528.

33a:

[α]$_D$$^{22.8}$ = -33.2 (c = 1.6, $CHCl_3$).

IR (thin film): 3358, 3029, 2959, 2950, 2856, 1738, 1654, 1446, 1374, 1365, 1305, 1275, 1228, 1216, 1205, 1175, 1160, 1102, 1074, 1058.

^{1}H NMR (600 MHz, $CDCl_3$): δ 5.14 (q, J = 1.9 Hz, 1H), 3.78 (dd, J = 10.5, 7.2 Hz, 1H), 2.95 – 2.87 (m, 1H), 2.83 (ddd, J = 9.9, 7.9, 3.2 Hz, 1H), 2.29 – 2.26 (m, 2H), 2.22 (dd, J = 13.6, 9.0 Hz, 1H), 2.01 (dd, J = 12.8, 7.3 Hz, 1H), 1.64 – 1.58 (m, 5H), 1.57 – 1.54 (m, 1H), 1.54 – 1.51 (m, 1H), 1.46 – 1.42 (m, 1H), 1.33 – 1.29 (m, 1H), 1.25 – 1.20 (m, 2H), 1.01 – 0.99 (m, 1H), 0.95 (s, 3H), 0.94 (d, J = 6.4 Hz, 3H), 0.89 (s, 3H), 0.86 (d, J = 6.6 Hz, 3H).

^{13}C NMR (100 MHz, $CDCl_3$): δ 143.3, 123.4, 78.8, 68.4, 61.1, 55.6, 54.0, 47.9, 46.6, 45.4, 40.8, 38.0, 33.4, 29.8, 28.2, 23.0, 22.9, 20.7, 15.4, 14.6.

HRMS m/z (APCI): calc. for $C_{20}H_{33}O$ $[M+H]^+$: 289.2526, found: 289.2524

34b:

[α]$_D$$^{22.4}$ = -16.6 (c = 0.5, $CHCl_3$).

IR (thin film): 3427, 2949, 2927, 2866, 1660, 1444, 1373, 1259, 1171, 1081.

^{1}H NMR (400 MHz, $CDCl_3$): δ 5.29 (dt, J = 3.5, 1.8 Hz, 1H), 3.59 (s, 1H), 3.17 – 2.96 (m, 1H), 2.88 (m, 1H), 2.64 – 2.46 (m, 1H), 2.42 – 2.35 (m, H), 2.02 – 1.91 (m, 2H), 1.83 (d, J = 12.2 Hz, 1H), 1.79 – 1.69 (m, 1H), 1.65 (dt, J = 3.0, 1.5 Hz, 3H), 1.63 – 1.54 (m, 2H), 1.49 – 1.43 (m, 1H), 1.42 – 1.39 (m, 1H), 1.39 – 1.32 (m, 2H), 1.15 (d, J = 6.8 Hz, 4H), 1.06 (s, 3H), 0.87 (d, J = 6.3 Hz, 3H), 0.81 (d, J = 6.4 Hz, 3H).

^{13}C NMR (100 MHz, $CDCl_3$): δ 145.1, 123.2, 79.3, 60.4, 50.8, 45.2, 44.5, 43.8, 37.5, 37.2, 37.1, 34.5, 32.7, 28.5, 28.2, 22.5, 22.3, 21.2, 18.4, 14.6.

HRMS m/z (APCI): calc. for $C_{20}H_{33}O$ $[M+H]^+$: 289.2526, found: 289.2528

33a and **34a** was yielded as a mixture of yellow oil. The mixture in DCM (10 mL, ca. 0.1 M) was treated with DMP (1.2 equiv, 508 mg, 1.2 mmol) and $NaHCO_3$ (2.0 equiv, 168 mg, 2 mmol) at 0 °C. The reaction was warmed to room temperature and stirred for another 2 hours. The reaction was quenched with $Na_2S_2O_3$ and extracted with EtOAc. The combined organic layers were washed with brine and concentrated under reduced pressure. The crude product was subjected to flash chromatography (hexane : EtOAc = 50 : 1 to hexane : EtOAc = 20 : 1) to yield ketone **33** (112 mg, 11%) and ketone **34** (95 mg, 10%) as colorless oils.

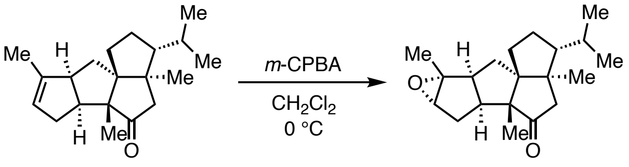

33 90% **49**

m-CPBA (1.5 equiv, 77% *m*-CPBA, 680 mg, 3.037 mmol) was added portionwise to **33** (1.0 equiv, 580 mg, 2.025 mmol) and $NaHCO_3$ (1.5 equiv, 255 mg, 3.037 mmol) in DCM (20 mL, 0.1 M) solution at 0 °C). The reaction was stirred for 1 h at the same temperature before it was quenched with $Na_2S_2O_3$. The mixture was extracted with EtOAc. The combined organic layers were washed with $NaHCO_3$ and brine, dried over Na_2SO_4, and concentrated under reduced pressure. The crude product was subjected to flash chromatography (hexanes : EtOAc = 5 :1) to yield epoxide **49** (550 mg, 90%) as a white solid. (**Note: *m*-CPBA is a strong oxidant and explosive and should be used with extreme caution.**)

m.p. 106 °C–108 °C.

$[\alpha]_D^{21.3}$ = –171.6 (*c* = 0.5, $CHCl_3$).

IR (neat): 3020, 2969, 2957, 2941, 2899, 2867, 1726, 1686, 1481, 1452, 1375, 1364, 1295, 1285, 1254, 1230, 1220, 1194, 1144, 1101, 1092, 1070, 1051.

^{1}H NMR (400 MHz, $CDCl_3$): δ 3.20 (s, 1H), 2.53 (d, *J* = 17.5 Hz, 1H), 2.30 – 2.08 (m, 4H), 2.00 (dd, *J* = 13.9, 8.1 Hz, 1H), 1.86 (ddd, *J* = 13.9, 9.4, 5.0 Hz, 1H), 1.81 – 1.70 (m, 1H), 1.70 – 1.59 (m, 1H), 1.59 – 1.47 (m, 2H), 1.44 – 1.30 (m, 5H), 1.14 – 0.99 (m, 7H), 0.92 (d, *J* = 6.6 Hz, 3H), 0.83 (d, *J* = 6.6 Hz, 3H).

^{13}C NMR (100 MHz, $CDCl_3$): δ 224.4, 67.0, 66.8, 61.6, 60.0, 55.4, 51.2, 50.3, 49.1, 46.6, 38.1, 33.4, 31.2, 29.2, 28.9, 22.7, 22.3, 17.2, 16.7, 15.8.

HRMS m/z (APCI): calc. for $C_{20}H_{31}O_2^+$ $[M+H]^+$: 303.2319, found: 303.2312.

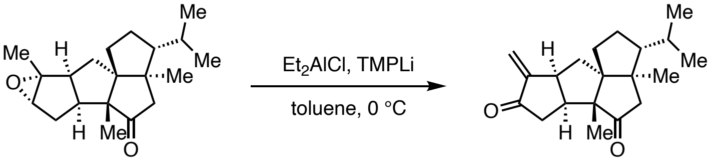

49 99% **49a**

A flame dried 100 mL round-bottom flask was charged with 2,2,6,6-tetramethylpiperidide (4.0 equiv, 1.66 mL, 9.73 mmol) and toluene (50 mL, 0.19 M). This solution was cooled to 0 °C and a solution of ^{n}BuLi (4.0 equiv, 2.5 M in hexane, 3.9 mL, 9.73 mmol) was added dropwise. The stirring was continued for 30 min at the same temperature. The reaction mixture changed from a colorless solution to a bright yellow suspension. A solution of Et_2AlCl (4.0 equiv, 1.0 M in hexane, 9.73 mL, 9.73 mmol) was added, upon which the yellow color disappeared. The resulting white suspension was stirred for 40 min at 0 °C, and a THF solution of epoxide **49** (1.0 equiv, 736 mg, 2.43 mmol) was added dropwise. After full conversion monitored

by TLC, the reaction mixture was quenched with saturated Rochelle salt and diluted with EtOAc. This biphasic mixture was stirred vigorously for 30 min, until both layers turned clear. The aqueous layer was extracted with EtOAc. The combined organic layers were washed with brine, dried over Na_2SO_4, and concentrated under reduced pressure. The crude product was subjected to flash chromatography (hexanes : EtOAc = 2:1) to yield allylic alcohol **49a** (730 mg, 99%) as a white solid.

m.p. 72 °C–73 °C.

$[\alpha]_D^{21.6}$ = –59.4 (c = 0.5, $CHCl_3$).

IR(neat): 3420, 2962, 2928, 2870, 1728, 1712, 1658, 1473, 1448, 1411, 1376, 1365, 1296, 1257, 1226, 11195, 1175, 1153, 1143, 1123, 1102, 1079,.

^{1}H NMR (400 MHz, $CDCl_3$): δ 5.14 (t, J = 1.5 Hz, 1H), 4.99 (d, J = 1.5 Hz, 1H), 4.48 (q, J = 4.1 Hz, 1H), 2.90 – 2.66 (m, 2H), 2.58 (d, J = 17.9 Hz, 1H), 2.28 (dd, J = 14.4, 8.4 Hz, 1H), 2.18 (d, J = 18.0 Hz, 1H), 1.90 – 1.70 (m, 4H), 1.66 – 1.43 (m, 4H), 1.43 – 1.29 (m, 2H), 1.17 (dt, J = 10.5, 8.7 Hz, 1H), 1.06 (d, J = 11.4 Hz, 6H), 0.93 (d, J = 6.5 Hz, 3H), 0.84 (d, J = 6.5 Hz, 3H).

^{13}C NMR (100 MHz, $CDCl_3$): δ 224.6, 159.1, 109.6, 74.9, 66.3, 60.9, 55.8, 52.8, 50.9, 48.8, 45.9, 43.1, 36.4, 33.0, 31.0, 29.3, 22.8, 22.3, 17.4, 16.9.

HRMS m/z (APCI): calc. for $C_{20}H_{31}O_2^+$ $[M+H]^+$: 303.2319, found:303.2324.

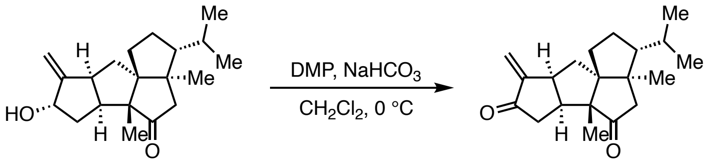

49a 94% **50**

Allylic alcohol **49a** (1.0 equiv, 620 mg, 2.05 mmol) in DCM (15 mL, 0.14 M) was treated with DMP (1.5 equiv, 1.3 g, 3.07 mmol) and $NaHCO_3$ (3.0 equiv, 516 mg, 6.15 mmol) at 0 °C. The reaction was warmed to room temperature and stirred for another 1 h. The reaction was quenched with $Na_2S_2O_3$ and extracted with EtOAc. The combined organic layers were washed with brine and concentrated under reduced pressure. The crude product was subjected to flash chromatography (hexanes : EtOAc =5: 1) to yield enone **50** (576 mg, 94%) and as a white solid.

m.p. 96 °C–99 °C.

$[\alpha]_D^{21}$ = –35 (c = 0.3, $CHCl_3$).

IR (neat): 2990, 2972, 2938, 2906, 2868, 1723, 1689, 1645, 1472, 1459, 1453, 1405, 1386, 1375, 1362, 1326, 1291, 1281, 1270, 1259, 1250, 1229, 1189, 1163, 1143, 1110, 1093, 1069.

^{1}H NMR (600 MHz, $CDCl_3$): δ 5.92 (dd, J = 1.9, 0.9 Hz, 1H), 5.24 (dd, J = 1.7, 0.9 Hz, 1H), 3.12 – 2.94 (m, 1H), 2.73 – 2.56 (m, 2H), 2.46 – 2.35 (m, 2H), 2.33 – 2.28 (m, 1H), 2.20 (d, J = 18.2 Hz, 1H), 1.88 –

1.72 (m, 2H), 1.70 – 1.49 (m, 3H), 1.47 – 1.32 (m, 1H), 1.25 – 1.15 (m, 1H), 1.08 (d, J = 3.4 Hz, 6H), 0.93 (d, J = 6.5 Hz, 3H), 0.85 (d, J = 6.5 Hz, 3H).

^{13}C NMR (150 MHz, CDCl$_3$): δ 223.9, 205.6, 148.4, 117.1, 66.6, 61.2, 55.9, 51.1, 48.9, 48.6, 45.1, 45.1, 42.7, 39.4, 33.4, 31.1, 29.4, 22.7, 22.3, 17.9, 17.0.

HRMS m/z (APCI): calc. for $C_{20}H_{29}O_2^+$ $[M+H]^+$: 301.2162, found: 301.2154.

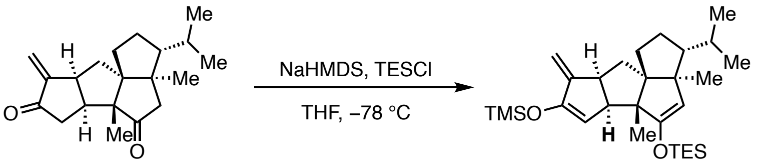

50 83% **50a**

A flame dried 25 mL round-bottom flask charged with enone **50** (1.0 equiv, 300 mg, 1.0 mmol) was added anhydrous THF (15 mL, 0.067 M). The solution was cooled to –78 °C and NaHMDS (3.0 equiv, 1.0 M in THF, 3 mL, 3.0 mmol) was added dropwise. The reaction mixture was stirred for 1 h at the same temperature. TESCl (3.0 equiv, 0.504 mL, 3 mmol) was added dropwise, and the mixture was allowed to warm to room temperature. The reaction was diluted with hexanes and quenched with saturated aqueous $NaHCO_3$ solution. The aqueous layer was extracted with hexanes. The combined organic layers were washed with brine, dried over Na_2SO_4 and concentrated at reduced pressure. The crude product was subjected to flash chromatography (hexanes) to yield silyl enol ether **50a** (441 mg, 83 %) as a pale-yellow liquid.

$[\alpha]_D^{20}$ = 25.1 (c = 1.0, CHCl$_3$).

IR (thin film): 2953, 2911, 2875, 1631, 1604, 1458, 1412, 1371, 1335, 1292, 1247, 1195, 1161, 1125, 1072.

^{1}H NMR (600 MHz, CDCl$_3$): δ 5.06 (td, J = 1.9, 0.9 Hz, 1H), 4.96 (dt, J = 2.0, 1.0 Hz, 1H), 4.70 (m, 1H), 4.45 (s, 1H), 3.17 (dd, J = 7.2, 2.9 Hz, 1H), 3.07 – 2.94 (m, 1H), 2.33 (dd, J = 13.5, 8.5 Hz, 1H), 1.80 – 1.64 (m, 2H), 1.56 – 1.42 (m, 2H), 1.39 – 1.20 (m, 3H), 1.06 – 0.99 (m, 21H), 0.97 (s, 3H), 0.93 (d, J = 6.4 Hz, 3H), 0.86 (d, J = 6.6 Hz, 3H), 0.79 – 0.67 (m, 13H).

^{13}C NMR (150 MHz, CDCl$_3$): δ 157.0, 154.5, 151.9, 114.3, 109.8, 100.0, 66.3, 59.8, 57.7, 54.3, 52.9, 44.9, 44.4, 34.5, 30.5, 29.1, 22.7, 22.7, 19.9, 17.0, 6.8, 6.7, 4.89, 4.85.

HRMS m/z (APCI): calc. for $C_{32}H_{55}O_2Si_2^+$ $[M-H]^+$: 527.3735, found:527.3748.

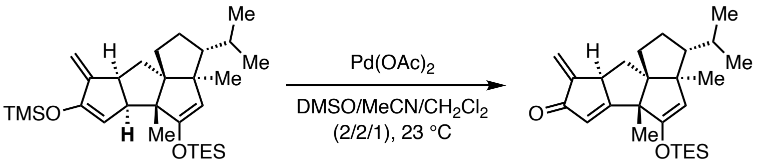

50a 86% **51**

Palladium acetate (1.1 equiv, 206 mg, 0.917 mmol) was added to a vial charged with silyl enol ether **50a** (1.0 equiv, 441 mg, 0.834 mmol) and DMSO/MeCN/CH_2Cl_2 (4 mL/4 mL/2 mL), and the resulting mixture was stirred for 1 h under air. The reaction mixture was filtered through a pad of Celite. The filtrate was diluted with water and extracted with hexanes. The combined organic layers were washed with brine, dried over Na_2SO_4, and concentrated under reduced pressure. The crude product was subjected to flash chromatography (hexanes : EtOAc =10 : 1) to afford **51** (297 mg, 86%) as a pale yellow liquid.

$[\alpha]_D^{21}$ = 142.9 (c = 0.5, $CHCl_3$).

IR (neat): 2969, 2954, 29123, 2874, 1730, 1703, 1653, 1614, 1457, 1413, 1371, 1305, 1291, 1272, 1248, 1233, 1217, 1206, 170, 1121, 1085, 1072.

^{1}H NMR (600 MHz, $CDCl_3$): 6.10 – 6.00 (m, 1H), 5.96 (dd, J = 2.0, 1.0 Hz, 1H), 5.27 (d, J = 1.6 Hz, 1H), 4.75 (s, 1H), 3.36 (ddd, J = 13.2, 7.5, 2.0 Hz, 1H), 2.55 (dd, J = 12.9, 7.3 Hz, 1H), 2.00 – 1.89 (m, 1H), 1.73 (dt, J = 11.8, 5.9 Hz, 1H), 1.61 – 1.55 (m, 1H), 1.34 (dd, J = 13.5, 6.9 Hz, 1H), 1.28 (s, 3H), 1.24 (td, J = 11.7, 6.9 Hz, 1H), 1.18 (ddd, J = 12.8, 9.3, 5.0 Hz, 1H), 1.04 (d, J = 4.4 Hz, 4H), 0.96 – 0.90 (m, 12H), 0.89 (d, J = 6.6 Hz, 3H), 0.67 – 0.57 (m, 6H).

^{13}C NMR (150 MHz, $CDCl_3$): δ 197.8, 191.3, 150.9, 147.4, 124.3, 115.0, 113.9, 65.8, 62.9, 58.3, 55.3, 45.8, 40.9, 36.3, 29.0, 28.7, 22.9, 22.5, 18.6, 16.9, 6.6, 4.7.

HRMS m/z (APCI): calc. for $C_{26}H_{41}O_2Si^+$ $[M+H]^+$: 413.2870, found: 413.2881.

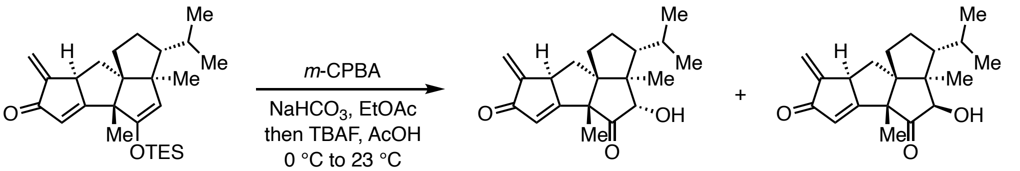

51 57% (**52**/**53** = 1.5/1) **52** (major) **53**

m-CPBA (1.5 equiv, 77% *m*-CPBA, 38 mg, 0.17 mmol) was added to a suspension of $NaHCO_3$ (10 equiv, 95 mg, 1.13 mmol) in EtOAc (4.5 mL, 0.025 M). The mixture was pre-stirred for 30 min and then cooled to 0 °C. A solution of silyl enol ether **51** (1.0 equiv, 47 mg, 0.11 mmol) in EtOAc (0.5 mL) was added dropwise. The mixture was warmed to room temperature and stirred for 5 h. Upon full consumption of silyl enol ether **51**, AcOH (3.0 equiv, 20 mg, 0.339 mmol) and TBAF (2.1 equiv, 1 M in THF, 0.24 mL, 0.24 mmol) was added. The mixture was stirred for additional 20 min before it was quenched with water and extracted with EtOAc. The combined organic layers were washed with brine, dried over Na_2SO_4 and concentrated under reduced pressure. The crude product was subjected to flash chromatography (hexanes : EtOAc = 6 : 1 to hexanes : EtOAc = 4 :1 to hexanes : EtOAc = 3 : 1) to yield enone **52** and enone **53** as colorless oils. (**Note: *m*-CPBA is a strong oxidant and explosive and should be used with extreme caution.**)

52: 12.1 mg, 34%, colorless oil.

$[\alpha]_D^{21.3}$ = –47.8 (c = 0.1, CH_2Cl_2).

IR (thin film): 3439, 2959, 2871, 1747, 1701, 1651, 1605, 1373, 1325, 1277, 1204, 1127, 1204, 1097, 1050.

^{1}H NMR (400 MHz, $CDCl_3$): 6.18 (d, J = 2.2 Hz, 1H), 5.97 (dd, J = 2.0, 0.7 Hz, 1H), 5.29 (dt, J = 1.5, 0.8 Hz, 1H), 4.24 (s, 1H), 3.57 – 3.51 (m, 1H), 2.71 – 2.55 (m, 2H), 1.96 – 1.79 (m, 2H), 1.75 – 1.53 (m, 3H), 1.53 – 1.41 (m, 2H), 1.40 (s, 3H), 1.05 – 1.00 (m, 6H), 0.92 (d, J = 6.6 Hz, 3H).

^{13}C NMR (150 MHz, $CDCl_3$): δ 215.7, 196.7, 184.9, 147.2, 126.7, 115.6, 82.0, 66.8, 57.3, 51.9, 46.6, 39.3, 36.1, 29.7, 29.1, 29.1, 23.2, 21.9, 19.6, 11.1.

HRMS m/z (APCI): calc. for $C_{20}H_{27}O_3^+$ $[M+H]^+$: 315.1955, found: 315.1962.

53: 8 mg, 23%, colorless oil.

$[\alpha]_D^{20.9}$ = 96.5 (c = 0.1, CH_2Cl_2).

IR (thin film): 3367, 2956, 2926, 2871, 1477, 1698, 1649, 1605, 1455, 1373, 1322, 1266, 1244, 1209, 1132, 1069.

^{1}H NMR (600 MHz, $CDCl_3$): 6.15 (d, J = 2.2 Hz, 1H), 6.04 (d, J = 1.9 Hz, 1H), 5.39 (s, 1H), 4.37 (s, 1H), 3.47 – 3.40 (m, 1H), 2.76 (s, 1H), 2.63 (dd, J = 13.5, 7.8 Hz, 1H), 2.16 – 2.11 (m, 1H), 1.91 (ddd, J = 14.0, 8.9, 5.6 Hz, 1H), 1.73 – 1.58 (m, 5H), 1.40 (s, 3H), 1.37 (s, 3H), 0.91 (d, J = 6.9 Hz, 3H), 0.88 (d, J = 6.7 Hz, 3H).

^{13}C NMR (150 MHz, $CDCl_3$): δ 213.3, 195.9, 183.3, 146.5, 126.8, 116.3, 85.0, 67.0, 56.8, 53.8, 52.0, 46.6, 37.3, 32.8, 28.3, 24.8, 23.2, 19.7, 17.3, 16.4.

HRMS m/z (APCI): calc. for $C_{20}H_{27}O_3^+$ $[M+H]^+$: 315.1955, found: 315.1959.

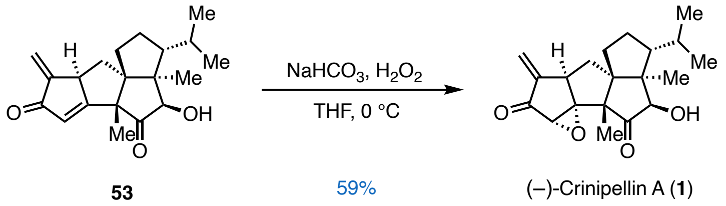

To a cold solution of the enone **53** (1.0 equiv, 6 mg, 0.019 mmol) in THF/H_2O (0.8 mL/0.8 mL) at 0 °C was added $NaHCO_3$ (16.0 equiv, 26 mg, 0.304 mmol). After 5 min, H_2O_2 (26.0 equiv, 32 wt% solution, 53 μL, 0.496 mmol) was quickly added. The reaction was monitored with TLC every 2 min. When most of the starting material disappears, the resulting mixture was diluted with cold EtOAc and H_2O, and washed with brine. The combined organic layers were dried over Na_2SO_4, filtered, and concentrated under reduced pressure. The residue was purified by column chromatography on silica gel (hexanes : EtOAc = 3:1) to

give **crinipellin A (1)** (3.7 mg, 59%) as a colorless oil. (**Note: H_2O_2 is a strong oxidant and explosive and should be used with extreme caution.**)

[α]$_D^{21.8}$ = –114.6 (c = 0.1, $CHCl_3$).

IR (thin film): 3466, 2956, 2926, 2871, 1728, 1639, 1455, 1403, 1379, 1260, 1213, 1132, 1103, 1062.

^{1}H NMR (600 MHz, $CDCl_3$): δ 6.16 (d, J = 1.8 Hz, 1H), 5.50 (d, J = 1.5 Hz, 1H), 4.42 (s, 1H), 3.48 (t, J = 0.9 Hz, 1H), 3.13 – 3.06 (m, 1H), 2.82 (s, 1H), 2.53 (dd, J = 14.3, 7.6 Hz, 1H), 2.14 – 2.09 (m, 1H), 1.90 (ddd, J = 13.6, 8.7, 5.4 Hz, 1H), 1.67 – 1.59 (m, 3H), 1.41 (dd, J = 14.3, 13.0 Hz, 1H), 1.33 (s, 4H), 1.05 (s, 3H), 0.87 (d, J = 7.0 Hz, 3H), 0.84 (d, J = 6.8 Hz, 3H).

^{13}C NMR (150 MHz, $CDCl_3$): δ 214.4, 195.9, 145.4, 123.5, 84.7, 77.8, 62.2, 58.4, 53.9, 51.8, 50.0, 42.0, 37.8, 32.7, 28.2, 24.8, 23.2, 19.6, 16.2, 15.2.

HRMS m/z (APCI): calc. for $C_{20}H_{27}O_4^+$ $[M+H]^+$: 331.1904, found:331.1906.

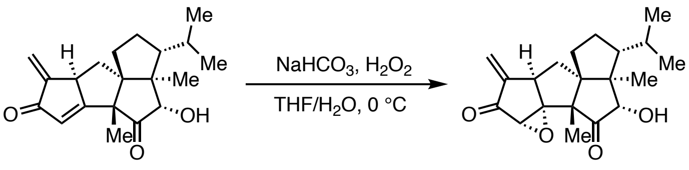

52 **54**

To a cold solution of the enone **52** (1.0 equiv, 12.1 mg, 0.0384 mmol) in THF/H_2O (1.5 mL/1.5 mL) at 0 °C was added $NaHCO_3$ (16.0 equiv, 52 mg, 0.616 mmol). After 5 min, H_2O_2 (26.0 equiv, 30 wt% solution, 113 µL, 1.0 mmol) was quickly added. The reaction was monitored with TLC every 2 min. When most of the starting material disappears, the resulting mixture was diluted with cold EtOAc and H_2O, and washed with brine. The combined organic layers were dried over Na_2SO_4, filtered, and concentrated under reduced pressure. The residue was purified by column chromatography on silica gel (hexanes : EtOAc = 3:1) to give **54** (8.7 mg, 69%) as a colorless oil. (**Note: H_2O_2 is a strong oxidant and explosive and should be used with extreme caution.**)

[α]$_D^{21.2}$ = –71.2 (c = 0.1, CH_2Cl_2).

IR (thin film): 3466, 2957, 2924, 2854, 1730, 1639, 1459, 1376, 1262, 1093.

^{1}H NMR (400 MHz, $CDCl_3$): δ 6.09 (d, J = 1.8 Hz, 1H), 5.45 – 5.35 (m, 1H), 4.10 (d, J = 2.9 Hz, 1H), 3.41 (s, 1H), 3.39 – 3.28 (m, 1H), 2.64 (d, J = 3.5 Hz, 1H), 2.59 (dd, J = 14.1, 8.1 Hz, 1H), 1.96 – 1.78 (m, 2H), 1.70 – 1.60 (m, 2H), 1.54 – 1.41 (m, 1H), 1.37 – 1.20 (m, 2H), 1.06 (d, J = 2.8 Hz, 6H), 1.01 (d, J = 6.5 Hz, 3H), 0.90 (d, J = 6.6 Hz, 3H).

^{13}C NMR (100 MHz, $CDCl_3$): δ 217.1, 197.1, 146.4, 122.8, 80.4, 78.0, 62.8, 56.8, 56.4, 52.0, 51.1, 41.6, 39.3, 35.4, 29.6, 28.6, 23.1, 22.1, 17.5, 10.6.

HRMS m/z (APCI): calc. for $C_{20}H_{27}O_4^+$ $[M+H]^+$: 331.1904, found: 331.1894.

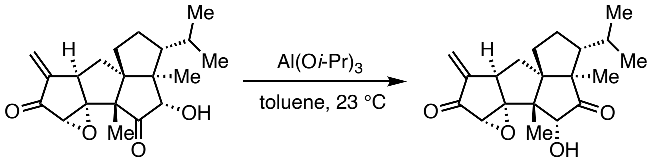

54 (–)-Crinipellin B (**2**)

To a stirred solution of epoxide **54** (1.0 equiv, 8.7 mg, 0.0263 mmol) in toluene (1.5 mL) at room temperature was added Al(O*i*-Pr)$_3$ (1.5 equiv, 8 mg, 0.0395 mmol). The resulting mixture was stirred for 1 h before it was quenched with an aqueous solution of Rochelle's salt. The layers were separated, and the aqueous layer was extracted with EtOAc. The combined organic layers were dried over Na_2SO_4 and concentrated in vacuo. The residue was purified by column chromatography on silica gel (hexanes : EtOAc = 2:1) to afford **crinipellin B (2)** (4 mg, 46%), along with epoxide **54** (1.5 mg, 17%) recovered.

$[\alpha]_D^{20.8}$ = –90.4 (*c* = 0.1, CH_2Cl_2).

IR (thin film): 3449, 2958, 2922, 2871, 2853, 1729, 11640, 1454, 1379, 1256, 1064.

^{1}H NMR (400 MHz, CDCl$_3$): δ 6.08 (d, *J* = 1.7 Hz, 1H), 5.43 – 5.30 (m, 1H), 4.75 (d, *J* = 6.4 Hz, 1H), 3.32 (t, *J* = 0.8 Hz, 1H), 2.94 (d, *J* = 6.4 Hz, 1H), 2.72 (ddd, *J* = 12.9, 6.9, 1.3 Hz, 1H), 2.30 – 2.22 (m, 2H), 2.06 – 1.98 (m, 1H), 1.78 – 1.72 (m, 1H), 1.66 – 1.58 (m, 2H), 1.55 – 1.48 (m, 1H), 1.34 (s, 3H), 1.24 (d, *J* = 1.2 Hz, 1H), 1.11 (s, 3H), 0.89 (d, *J* = 6.5 Hz, 3H), 0.81 (d, *J* = 6.5 Hz, 3H).

^{13}C NMR (100 MHz, CDCl$_3$): δ 217.5, 196.8, 145.1, 122.8, 79.8, 77.6, 63.6, 60.7, 57.5, 55.7, 43.4, 42.4, 38.9, 33.9, 30.1, 29.9, 22.7, 21.4 (2C), 10.3.

HRMS m/z (APCI): calc. for $C_{20}H_{27}O_4^+$ $[M+H]^+$: 331.1904, found: 331.1897.

C. NMR Comparison of Our Synthetic Samples and Previous Reports

Table S2. ^{1}H NMR (CDCl$_3$) Comparison of Natural[2] & Our Synthetic (–)-Crinipellin A (1)

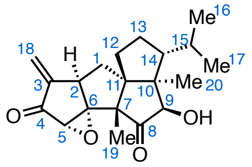

Crinipellin A (**1**)

No.	Natural, 400 MHz δ ^{1}H [ppm, mult, *J*(Hz)]	Synthetic, 600 MHz δ ^{1}H [ppm, mult, *J*(Hz)]
1a	1.41 (dd, *J* = 14.2, 13.2 Hz, 1 H)	1.41 (dd, *J* = 14.3, 13.0 Hz, 1H)
1b	2.53 (dd, *J* = 14.2, 7.5 Hz, 1 H)	2.53 (dd, *J* = 14.3, 7.6 Hz, 1H)
2	3.10 (ddddd, *J* = 13.2, 7.5, 1.4, 0.8, 0.5 Hz, 1 H)	3.13 – 3.06 (m, 1H)
3	–	–
4	–	–
5	3.48 (dd, *J* = 1.1, 0.8 Hz, 1 H)	3.48 (t, *J* = 0.9 Hz, 1H)
6	–	–
7	–	–
8	–	–
9	4.42 (d, *J* = 2.5 Hz, 1 H)	4.42 (s, 1H)
10	–	–
11	–	–
12a	1.89 (ddd, *J* = 8.7, 8.7, 5.4 Hz, 1 H)	1.90 (ddd, *J* = 13.6, 8.7, 5.4 Hz, 1H)
12b	1.61 (m, 1 H)	1.67 – 1.59 (m, 1H)
13a	1.61 (m, 1 H)	1.67 – 1.59 (m, 1H)
13b	1.61 (m, 1 H)	1.67 – 1.59 (m, 1H)
14	1.33 (doub., 1 H)	1.36 -1.30 (m, 1H)
15	2.11 (dsep, *J* = 7.0, 2.5 Hz, 1 H)	2.11 (pd, *J* = 6.9, 2.2 Hz, 1H)
16	0.85 (d, *J* = 7.0 Hz, 3 H)	0.87 (d, *J* = 7.0 Hz, 3H)
17	0.82 (d, *J* = 7.0 Hz, 3 H)	0.84 (d, *J* = 6.8 Hz, 3H)
18a	5.49 (ddd, *J* = 1.1, 0.5, 0.5 Hz, 1 H)	5.50 (d, *J* = 1.5 Hz, 1H)
18b	6.15 (dd, *J* = 1.4, 0.5 Hz, 1 H)	6.16 (d, *J* = 1.8 Hz, 1H)
19	1.04 (s, 3 H)	1.05 (s, 3H)
20	1.33 (s, 3 H)	1.33 (s, 3H)
9-OH	2.88 (d, *J* = 2.5 Hz, 1 H)	2.82 (s, 1H)

Table S3. ^{1}H NMR ($CDCl_3$) Comparison of of Lee's[3], Yang's[4], Ding's[5] & Our Synthetic (–)-Crinipellin A (1)

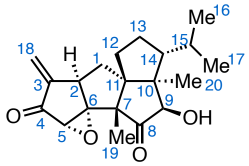

Crinipellin A (**1**)

No.	Lee's, 400 MHz δ ^{1}H [ppm, mult, J(Hz)]	Yang's, 400 MHz δ ^{1}H [ppm, mult, J(Hz)]	Ding's, 400 MHz	Ours, 600 MHz δ ^{1}H [ppm, mult, J(Hz)]
1a	1.39 (dd, J = 14.1, 13.1 Hz, 1 H)	1.39 (d, J = 14.1 Hz, 1 H)	1.41 (dd, J = 14.3, 13.0 Hz, 1 H)	1.41 (dd, J = 14.3, 13.0 Hz, 1H)
1b	2.51 (dd, J = 14.3, 7.5 Hz, 1 H)	2.53 (m, 1 H)	2.53 (dd, J = 14.3, 7.6 Hz, 1 H)	2.53 (dd, J = 14.3, 7.6 Hz, 1H)
2	3.13 – 3.03 (m, 1H)	3.10 (m, 1H)	3.12 – 3.07 (m, 1H)	3.13 – 3.06 (m, 1H)
3	–	–	–	–
4	–	–	–	–
5	3.46 (s, 1 H)	3.48 (s, 1 H)	3.48 (s, 1 H)	3.48 (t, J = 0.9 Hz, 1H)
6	–	–	–	–
7	–	–	–	–
8	–	–	–	–
9	4.40 (d, J = 2.9 Hz, 1 H)	4.42 (s, 1 H)	4.42 (d, J = 2.9 Hz, 1 H)	4.42 (s, 1H)
10	–	–	–	–
11	–	–	–	–
12a	1.93–1.82 (m, 1 H)	1.97–1.83 (m, 1H)	1.94–1.84 (m, 1 H)	1.90 (ddd, J = 13.6, 8.7, 5.4 Hz, 1H)
12b	1.68–1.56 (m, 1 H)	1.68–1.61 (m, 1H)	1.68–1.59 (m, 1 H)	1.67 – 1.59 (m, 1H)
13a	1.68–1.56 (m, 1 H)	1.68–1.61 (m, 1H)	1.68–1.59 (m, 1 H)	1.67 – 1.59 (m, 1H)
13b	1.68–1.56 (m, 1 H)	1.68–1.61 (m, 1H)	1.68–1.59 (m, 1 H)	1.67 – 1.59 (m, 1H)
14	1.34–1.30 (m, 1 H)	1.37–1.31 (m, 1H)	1.36–1.30 (m, 1 H)	1.36 -1.30 (m, 1H)
15	2.09 (dtd, J = 13.6, 6.6, 2.7 Hz, 1 H)	2.21–2.04 (m, 1H)	2.15–2.07 (m, 1 H)	2.11 (pd, J = 6.9, 2.2 Hz, 1H)
16	0.85 (d, J = 6.9 Hz, 3 H)	0.87 (d, J = 6.9 Hz, 3 H)	0.87 (d, J = 7.0 Hz, 3 H)	0.87 (d, J = 7.0 Hz, 3H)
17	0.82 (d, J = 6.8 Hz, 3 H)	0.84 (d, J = 6.8 Hz, 3 H)	0.84 (d, J = 6.8 Hz, 3 H)	0.84 (d, J = 6.8 Hz, 3H)
18a	5.48 (d, J = 1.3 Hz, 1 H)	5.50 (s, 1 H)	5.50 (d, J = 1.5 Hz, 1 H)	5.50 (d, J = 1.5 Hz, 1H)
18b	6.14 (d, J = 1.8 Hz, 1 H)	6.16 (d, J = 1.7 Hz, 1 H)	6.16 (d, J = 1.8 Hz, 1 H)	6.16 (d, J = 1.8 Hz, 1H)
19	1.03 (s, 3 H)	1.05 (s, 3 H)	1.05 (s, 3 H)	1.05 (s, 3H)
20	1.32 (s, 3 H)	1.33 (s, 3 H)	1.33 (s, 3 H)	1.33 (s, 3H)
9-OH	2.79 (d, J = 3.0 Hz, 1 H)	Not reported	2.81 (d, J = 3.0 Hz, 1 H)	2.82 (s, 1H)

Table S4. ^{13}C NMR ($CDCl_3$) Comparison of Natural[2] & Our Synthetic (–)-Crinipellin A (1)

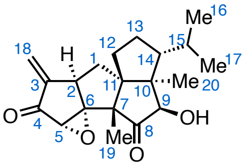

Crinipellin A (**1**)

No.	Natural, 100 MHz δ ^{13}C (ppm)	Synthetic, 150 MHz δ ^{13}C (ppm)
1	37.8	37.8
2	42.0	42.0
3	145.6	145.4
4	196.0	195.9
5	58.5	58.4
6	77.8	77.8
7	50.0	50.0
8	214.5	214.4
9	84.7	84.7
10	53.9	53.9
11	62.3	62.2
12	32.7	32.7
13	23.2	23.2
14	51.8	51.8
15	28.2	28.2
16	19.6	19.6
17	24.8	24.8
18	123.5	123.5
19	15.1	15.2
20	16.2	16.2

Table S5. ^{13}C NMR ($CDCl_3$) Comparison of of Lee's[3], Yang's[4], Ding's[5] & Our synthetic of (–)-Crinipellin A (1)

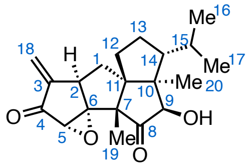

Crinipellin A (**1**)

No.	Lee's, 150 MHz<br>δ ^{13}C (ppm)	Yang's, 150 MHz<br>δ ^{13}C (ppm)	Ding's, 150 MHz<br>δ ^{13}C (ppm)	Ours, 150 MHz<br>δ ^{13}C (ppm)
1	37.8	38.0	38.0	37.8
2	42.0	42.2	42.1	42.0
3	145.5	145.7	145.5	145.4
4	196.0	196.1	196.1	195.9
5	58.4	58.6	58.6	58.4
6	77.8	78.0	77.9	77.8
7	50.0	50.2	50.1	50.0
8	214.4	214.6	214.6	214.4
9	84.7	84.9	84.8	84.7
10	53.9	54.1	54.1	53.9
11	62.3	62.5	62.4	62.2
12	32.7	32.9	32.8	32.7
13	23.2	23.4	23.3	23.2
14	51.8	52.0	51.9	51.8
15	28.2	28.4	28.3	28.2
16	19.6	19.8	19.8	19.6
17	24.8	25.0	25.0	24.8
18	123.4	123.6	123.7	123.5
19	15.2	15.4	15.4	15.2
20	16.2	16.4	16.4	16.2

Table S6. ^{1}H NMR ($CDCl_3$) Comparison of Natural[2] & Our Synthetic (–)-Crinipellin B (2)

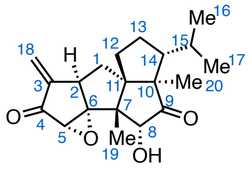

Crinipellin B (**2**)

No.	Natural, 400 MHz δ ^{1}H [ppm, mult, *J*(Hz)]	Synthetic, 600 MHz δ ^{1}H [ppm, mult, *J*(Hz)]
1a	Not reported	1.24 (d, *J* = 1.2 Hz, 1H)
1b	Not reported	2.30 – 2.22 (m, 1H)
2	Not reported	2.72 (ddd, *J* = 12.9, 6.9, 1.3 Hz, 1H)
3	–	–
4	–	–
5	Not reported	3.32 (t, *J* = 0.8 Hz, 1H)
6	–	–
7	–	–
8	–	–
9	4.73 (s, 1 H)	4.75 (d, *J* = 6.4 Hz, 1H)
10	–	–
11	–	–
12a	Not reported	2.06 – 1.98 (m, 1H),
12b	Not reported	1.78 – 1.72 (m, 1H)
13a	Not reported	1.66 – 1.58 (m, 1H)
13b	Not reported	1.66 – 1.58 (m, 1H)
14	Not reported	2.30 – 2.22 (m, 1H)
15	Not reported	1.55 – 1.48 (m, 1H)
16	Not reported	0.81 (d, J = 6.5 Hz, 3H)
17	Not reported	0.89 (d, J = 6.5 Hz, 3H)
18a	Not reported	6.08 (d, J = 1.7 Hz, 1H)
18b	Not reported	5.43 – 5.30 (m, 1H)
19	1.31 (s, 3 H)	1.34 (s, 3H)
20	1.08 (s, 3 H)	1.11 (s, 3H)
8-OH	Not reported	2.94 (d, J = 6.4 Hz, 1H)

Table S7. ^{1}H NMR ($CDCl_3$) Comparison of of Piers'[6], Yang's[4], Ding's[5] & Our synthetic (–)-Crinipellin B (2)

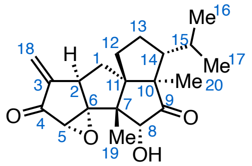

Crinipellin B (**2**)

No.	Piers', 400 MHz<br>δ ^{1}H [ppm, mult, J(Hz)]	Yang's, 400 MHz<br>δ ^{1}H [ppm, mult, J(Hz)]	Ding's, 400 MHz	Ours, 600 MHz<br>δ ^{1}H [ppm, mult, J(Hz)]
1a	1.24<br>(dd, J = 14.0, 13.0 Hz, 1 H)	1.25 (m, 1 H)	1.28–1.21 (m, 1 H)	1.24 (d, J = 1.2 Hz, 1H)
1b	2.30–2.20 (m, 1 H)	2.25 (m, 1 H)	2.29–2.22 (m, 1 H)	2.30 – 2.22 (m, 1H)
2	2.71<br>(ddm, J =13.0, 7.0 Hz, 1 H)	2.72 (m, 1 H)	2.72<br>(ddq, J = 12.9, 6.9, 1.4 Hz, 1 H)	2.72<br>(ddd, J = 12.9, 6.9, 1.3 Hz, 1H)
3	–	–	–	–
4	–	–	–	–
5	3.31 (s, 1 H)	3.32 (s, 1 H)	3.32 (s, 1 H)	3.32 (t, J = 0.8 Hz, 1H)
6	–	–	–	–
7	–	–	–	–
8	–	–	–	–
9	4.75 (d, J = 6.5 Hz, 1 H)	4.75 (s, 1 H)	4.75 (d, J = 6.3 Hz, 1 H)	4.75 (d, J = 6.4 Hz, 1H)
10	–	–	–	–
11	–	–	–	–
12a	2.06–1.97 (m,1 H)	2.02 (m, 1 H)	2.06–1.98 (m, 1 H)	2.06 – 1.98 (m, 1H),
12b	1.76<br>(ddd, J = 11.5, 10.0, 7.0 Hz, 1 H)	1.75 (m, 1 H)	1.75<br>(ddd, J = 11.5, 10.0, 6.7 Hz, 1 H)	1.78 – 1.72 (m, 1H)
13a	1.68–1.45 (m, 1 H)	1.59 (m, 1 H)	1.67–1.46 (m, 1 H)	1.66 – 1.58 (m, 1H)
13b	1.68–1.45 (m, 1 H)	1.59 (m, 1 H)	1.67–1.46 (m, 1 H)	1.66 – 1.58 (m, 1H)
14	2.30–2.20 (m, 1 H)	2.25 (m, 1 H)	2.29–2.22 (m, 1 H)	2.30 – 2.22 (m, 1H)
15	1.68–1.45 (m, 1 H)	1.59 (m, 1 H)	1.67–1.46 (m, 1 H)	1.55 – 1.48 (m, 1H)
16	0.81 (d, J = 6.5 Hz, 3H)	0.81 (d, J = 6.5 Hz, 3H)	0.81 (d, J = 6.5 Hz, 3 H)	0.81 (d, J = 6.5 Hz, 3H)
17	0.88 (d, J = 6.5 Hz, 3H)	0.89 (d, J = 6.5 Hz, 3H)	0.88 (d, J = 6.5 Hz, 3 H)	0.89 (d, J = 6.5 Hz, 3H)
18a	6.08 (d, J = 1.5 Hz, 1H)	6.08 (d, J = 1.4 Hz, 1H)	6.08 (d, J = 1.6 Hz, 1 H)	6.08 (d, J = 1.7 Hz, 1H)
18b	5.37 (brs, 1 H)	5.38 (s, 1 H)	5.38 (s, 1 H)	5.43 – 5.30 (m, 1H)
19	1.33 (s, 3 H)	1.34 (s, 3 H)	1.34 (s, 3 H)	1.34 (s, 3H)
20	1.11 (s, 3 H)	1.11 (s, 3 H)	1.11 (s, 3 H)	1.11 (s, 3H)
8-OH	2.93 (d, J = 6.5 Hz, 1 H)	Not reported	2.95 (d, J = 6.4 Hz, 1 H)	2.94 (d, J = 6.4 Hz, 1H)

Table S8. ^{13}C NMR ($CDCl_3$) Comparison of Natural[2] & Our Synthetic (–)-Crinipellin B (2)

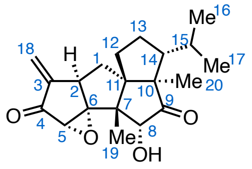

Crinipellin B (**2**)

No.	Natural, 100 MHz<br>δ ^{13}C (ppm)	Synthetic, 150 MHz<br>δ ^{13}C (ppm)
1	Not reported	37.8
2	Not reported	42.0
3	Not reported	145.4
4	Not reported	195.9
5	Not reported	58.4
6	Not reported	77.8
7	Not reported	50.0
8	79.80	214.4
9	217.44	84.7
10	Not reported	53.9
11	Not reported	62.2
12	Not reported	32.7
13	Not reported	23.2
14	Not reported	51.8
15	Not reported	28.2
16	Not reported	19.6
17	Not reported	24.8
18	Not reported	123.5
19	21.37	15.2
20	21.37	16.2

Table S9. ^{13}C NMR ($CDCl_3$) Spectroscopic Comparison of of Piers'[6], Yang's[4], Ding's[5] & Our Synthetic (–)-Crinipellin B (2)

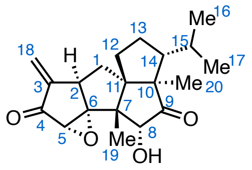

Crinipellin B (**2**)

No.	Piers', 75 MHz δ ^{13}C (ppm)	Yang's, 100 MHz δ ^{13}C (ppm)	Ding's, 100 MHz δ ^{13}C (ppm)	Ours, 150 MHz δ ^{13}C (ppm)
1	38.9	39.1	39.0	38.9
2	42.5	42.7	42.6	42.4
3	145.1	145.5	145.2	145.1
4	196.8	196.9	197.0	196.8
5	57.5	57.8	57.7	57.5
6	77.6	77.8	77.8	77.6
7	55.7	56.0	55.9	55.7
8	79.8	80.1	80.0	79.8
9	217.5	217.6	217.7	217.5
10	60.7	60.9	60.8	60.7
11	63.6	63.9	62.4	63.6
12	33.9	34.2	34.0	33.9
13	30.1	30.3	30.1	30.1
14	43.4	43.7	43.5	43.4
15	29.9	30.1	30.2	29.9
16	21.4	21.6	21.5	21.4
17	22.7	22.8	22.9	22.7
18	122.8	122.8	123.1	122.8
19	21.4	21.6	21.5	21.4
20	10.3	10.5	10.5	10.3

D. References

1. Prashad, M. Kim, H-Y. Har, D. Repic, O. Blacklock, T. J. *Tetrahedron Lett.* **1998**, *39*, 9369-9372.

2. Anke, T.; Heim, J.; Knoch, F.; Mocek, U.; Steffan, B.; Steglich, W. *Angew. Chem. Int. Ed.* **1985**, *24*, 709–711.

3. Kang, T.; Song, S. B.; Kim, W.-Y.; Kim, B. G.; Lee, H.-Y. *J. Am. Chem. Soc.* **2014**, *136*, 10274−10276.

4. Huang, Z.; Huang, J.; Qu, Y.; Zhang, W.; Gong, J.; Yang, Z. *Angew. Chem., Int. Ed*. **2018**, *57*, 8744−8748.

5. Zhao, Y.; Hu, J.; Chen, R.; Xiong, F.; Xie, H.; Ding, H. *J. Am. Chem. Soc.* **2022**, *144*, 2495−2500.

6. (a) Piers, E.; Renaud, J. *J. Org. Chem.* **1993**, *58*, 11−13. (b) Piers, E.; Renaud, J.; Rettig, S. J. *Synthesis* **1998**, 590−602.

E.^{1}H and ^{13}C NMR spectra

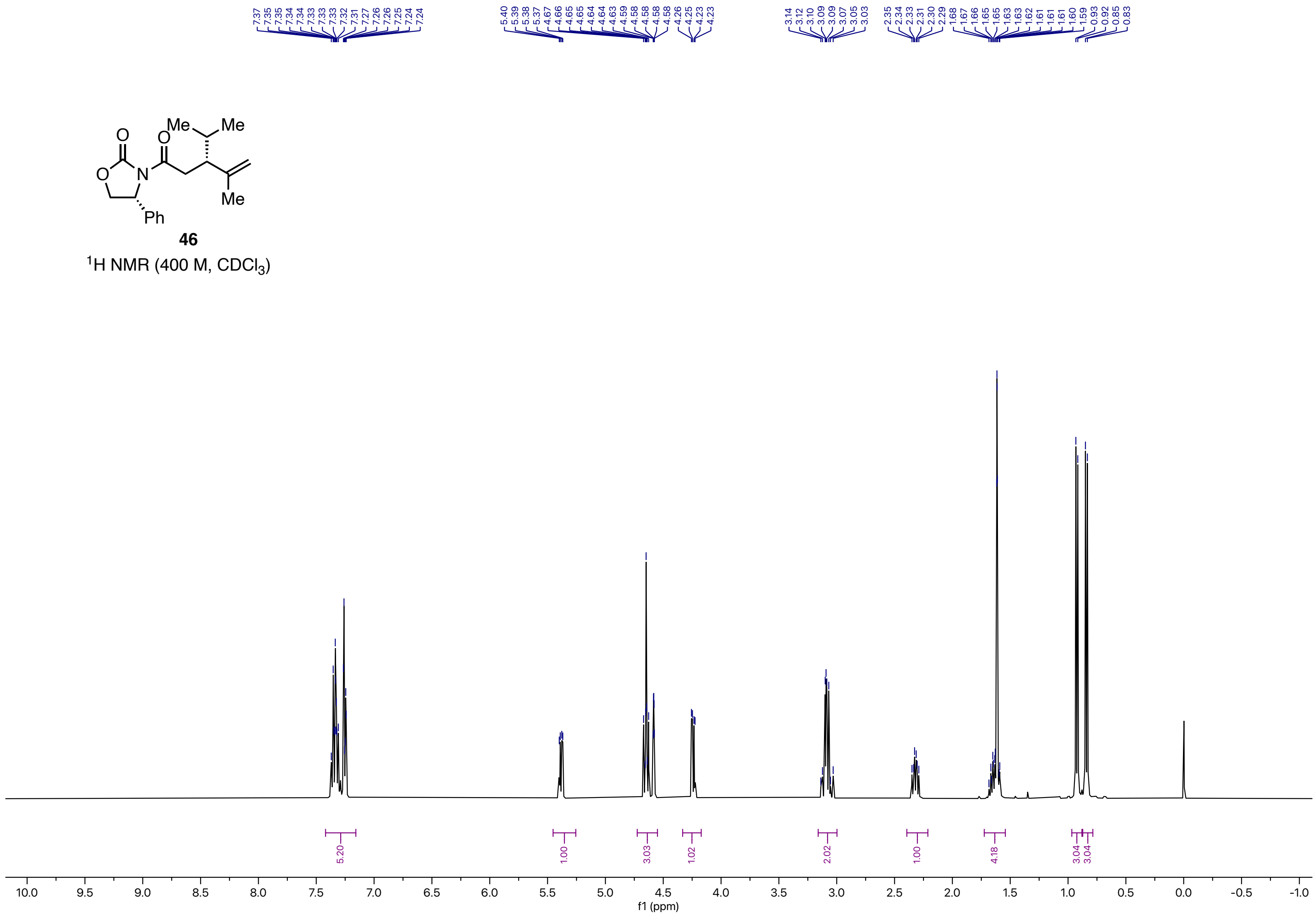

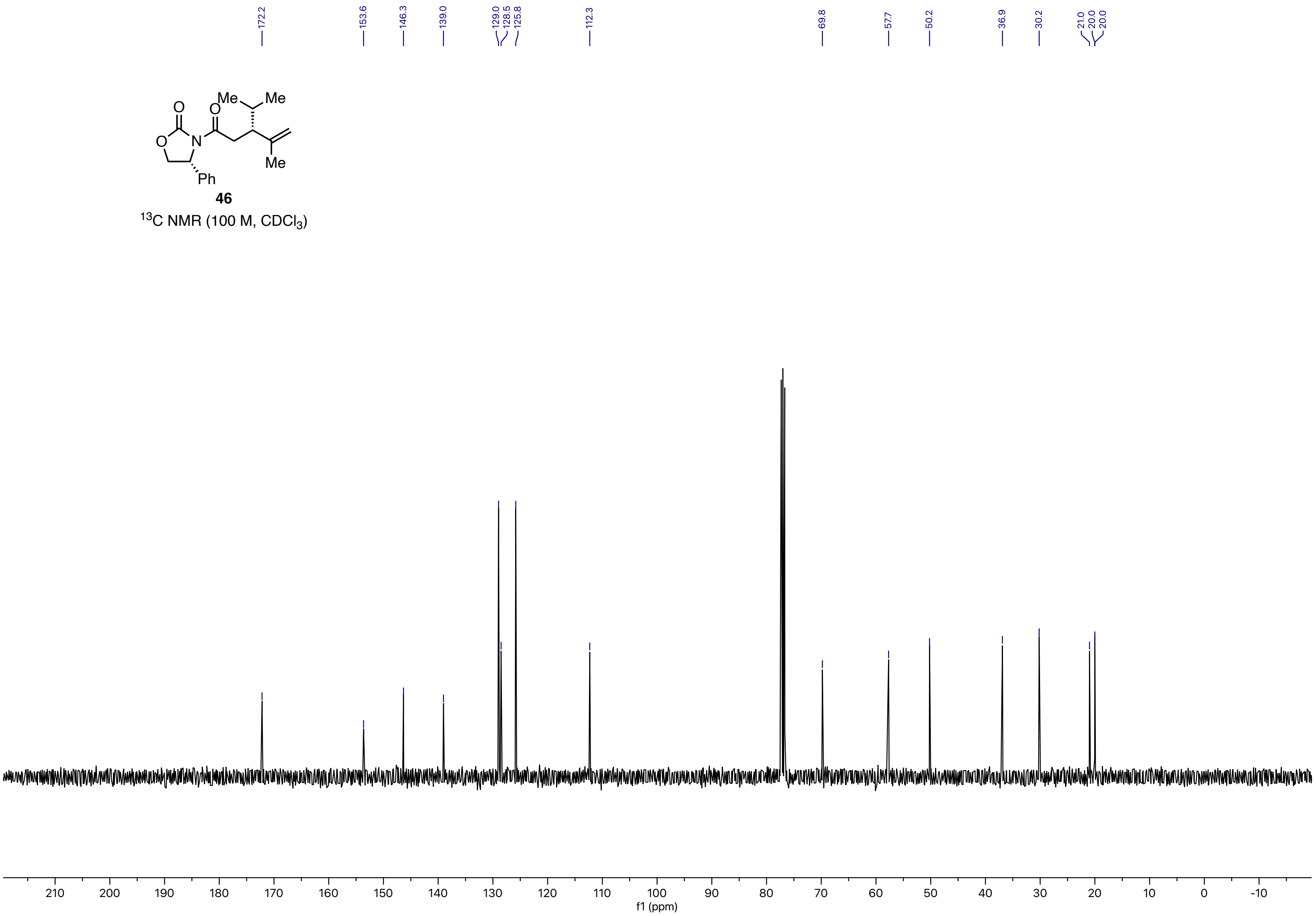

172.2
153.6
146.3
139.0
129.0
128.5
125.8
112.3
69.8
57.7
50.2
36.9
30.2
21.0
20.0
20.0
Me
Me
O
O
O
N
Me
Ph
46
^{13}C NMR (100 M, CDCl$_3$)
210
200
190
180
170
160
150
140
130
120
110
100
90
80
70
60
50
40
30
20
10
0
-10
f1 (ppm)

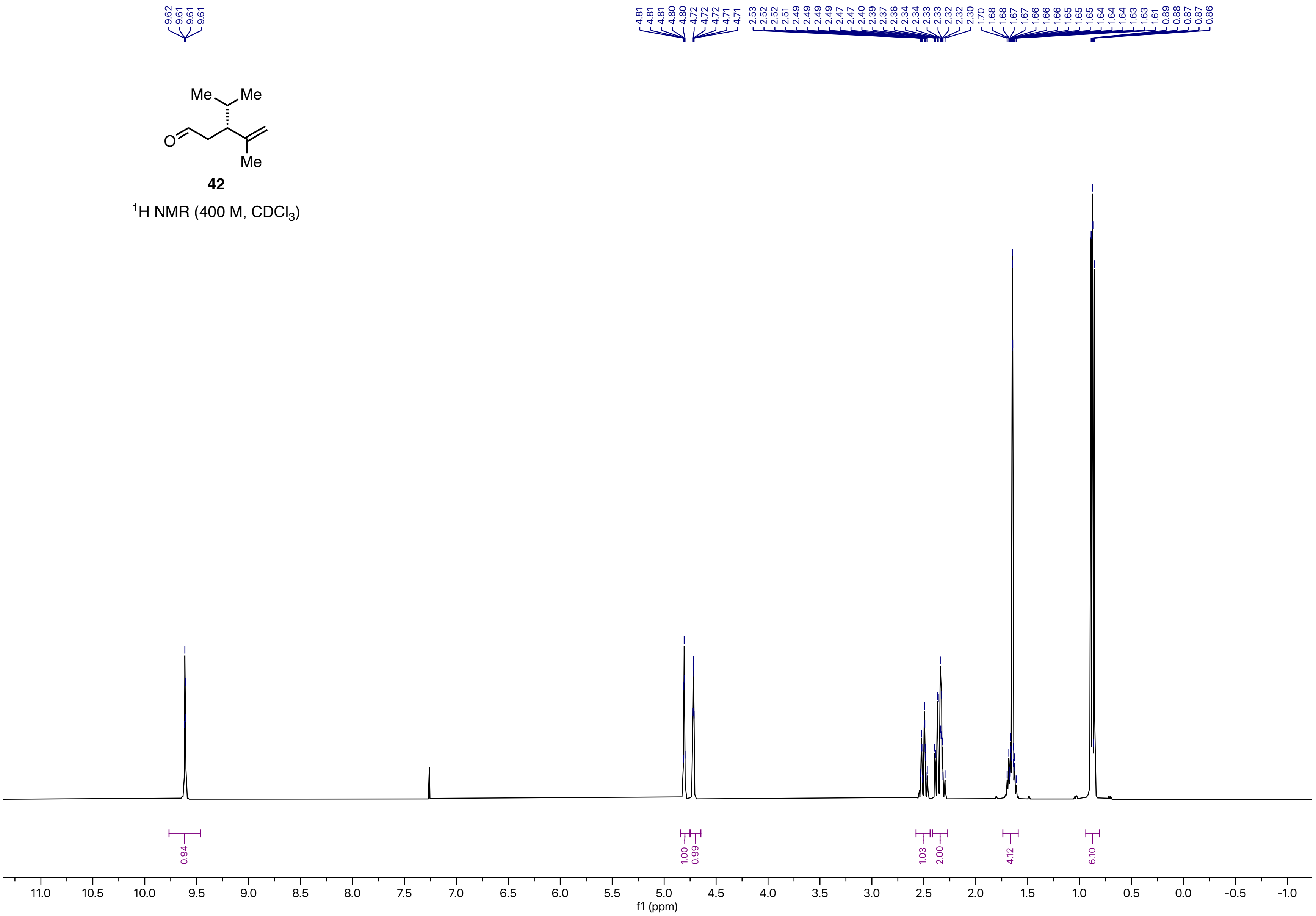

42

^{1}H NMR (400 M, $CDCl_3$)

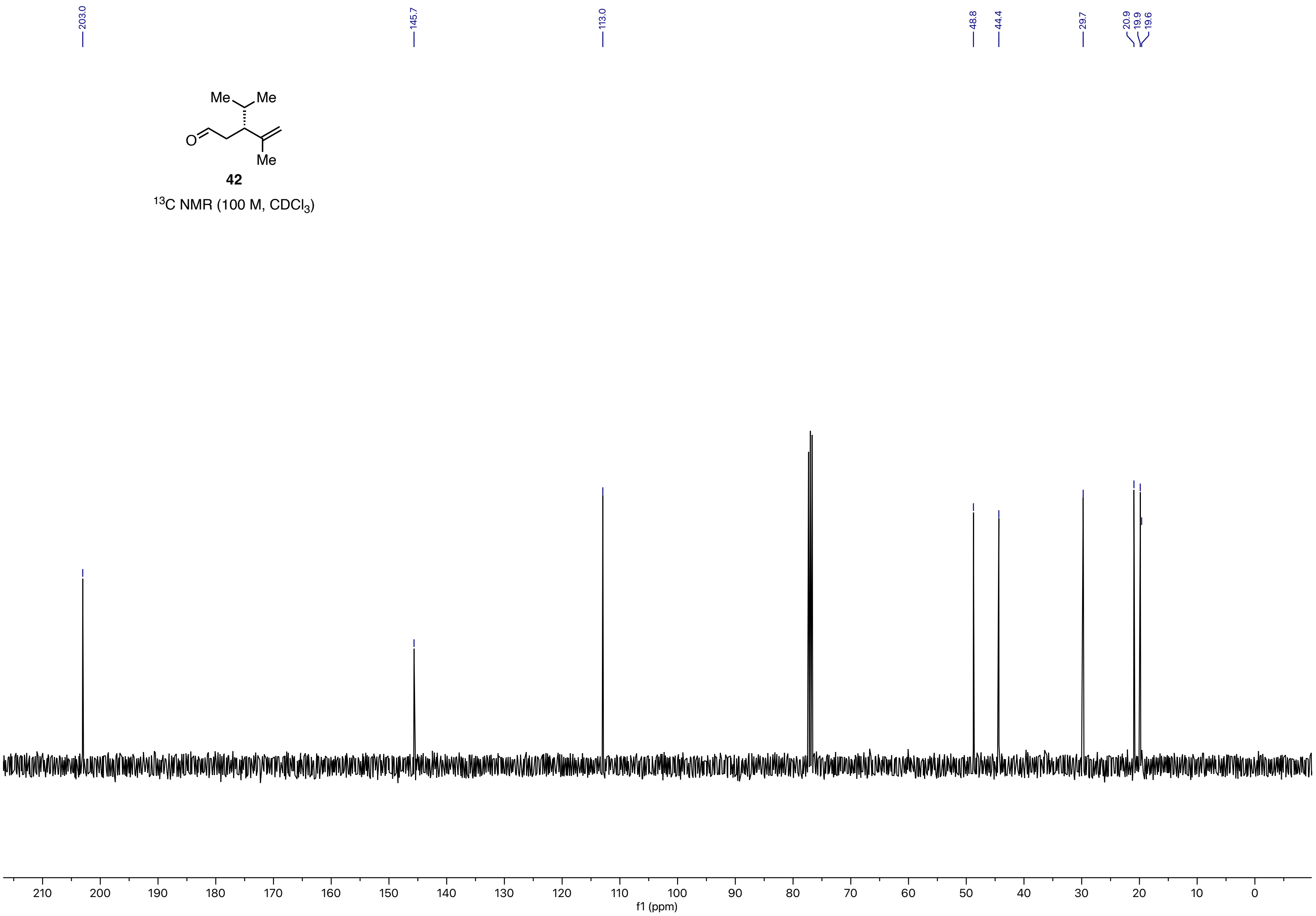

203.0
145.7
113.0
48.8
44.4
29.7
20.9
19.9
19.6
Me
Me
O
Me
42
^{13}C NMR (100 M, CDCl$_3$)
210 200 190 180 170 160 150 140 130 120 110 100 90 80 70 60 50 40 30 20 10 0
f1 (ppm)

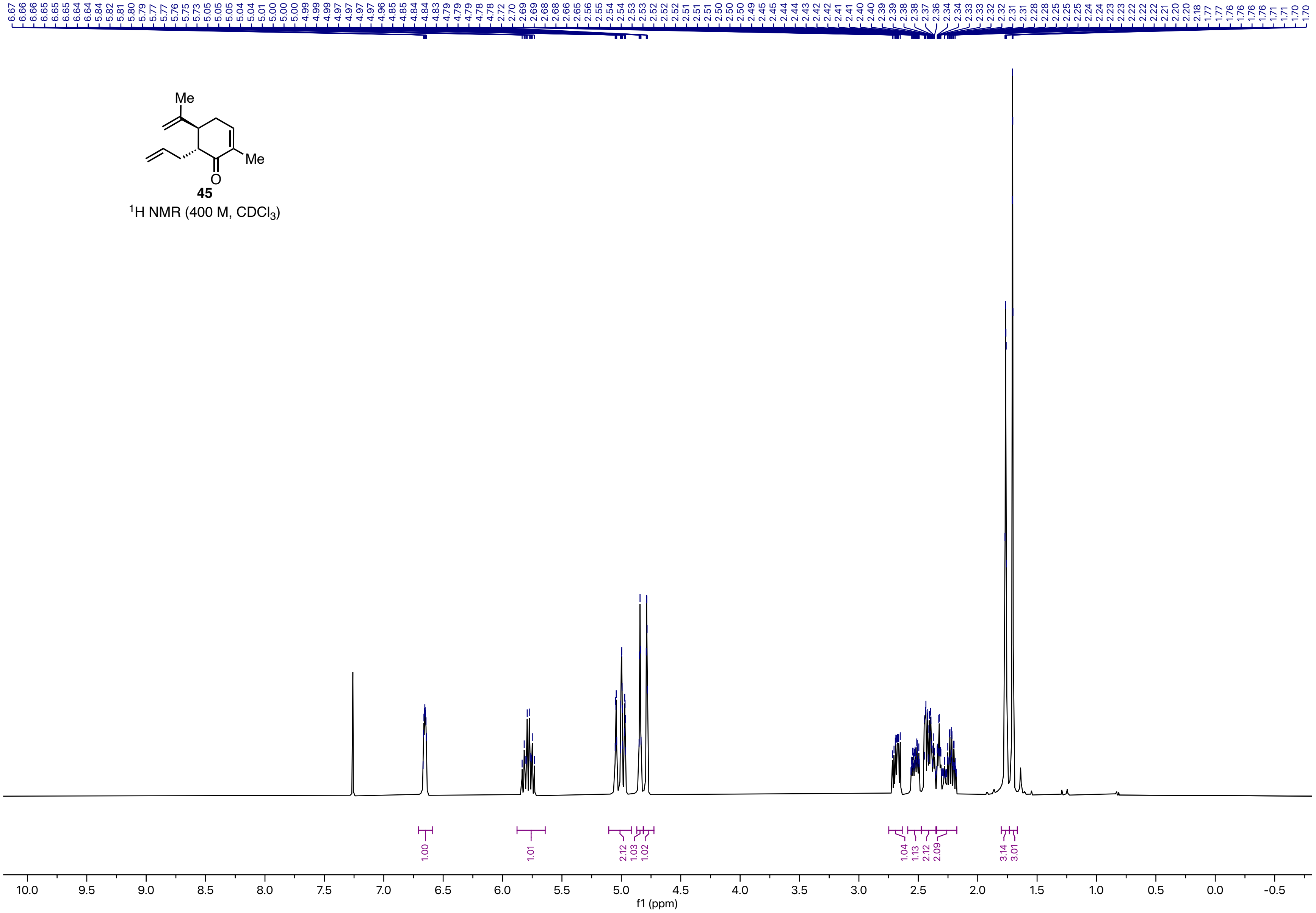
6.67 6.66 6.66 6.65 6.65 6.65 6.64 6.64 5.84 5.82 5.81 5.80 5.79 5.77 5.76 5.75 5.73 5.05 5.05 5.05 5.04 5.04 5.01 5.00 5.00 4.99 4.99 4.97 4.97 4.97 4.96 4.85 4.85 4.84 4.84 4.83 4.79 4.79 4.79 4.78 2.72 2.70 2.69 2.68 2.68 2.66 2.65 2.56 2.55 2.54 2.54 2.53 2.53 2.52 2.52 2.51 2.51 2.50 2.50 2.50 2.49 2.45 2.45 2.44 2.43 2.42 2.42 2.41 2.40 2.39 2.38 2.38 2.37 2.36 2.34 2.34 2.33 2.33 2.32 2.32 2.32 2.31 2.28 2.25 2.25 2.24 2.24 2.23 2.23 2.22 2.22 2.22 2.21 2.20 2.20 2.18 1.77 1.77 1.76 1.76 1.76 1.71 1.71 1.70 1.70
Me
Me
O
45
^{1}H NMR (400 M, $CDCl_3$)
1.00
1.01
2.12
1.03
1.02
1.04
1.13
2.12
2.09
3.14
3.01
10.0 9.5 9.0 8.5 8.0 7.5 7.0 6.5 6.0 5.5 5.0 4.5 4.0 3.5 3.0 2.5 2.0 1.5 1.0 0.5 0.0 -0.5
f1 (ppm)

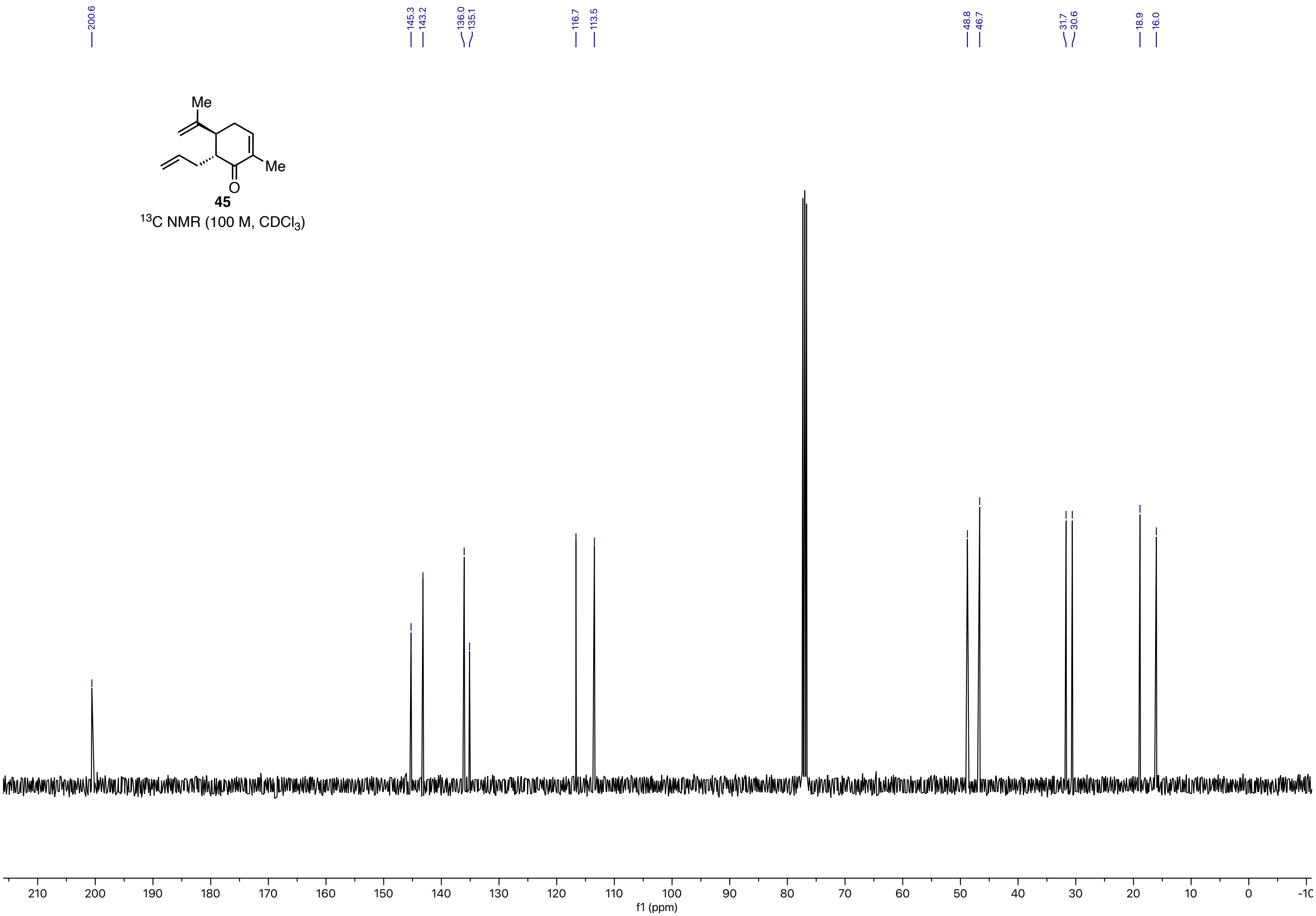
200.6
145.3
143.2
136.0
135.1
116.7
113.5
48.8
46.7
31.7
30.6
18.9
16.0
Me
Me
O
45
^{13}C NMR (100 M, $CDCl_3$)
210 200 190 180 170 160 150 140 130 120 110 100 90 80 70 60 50 40 30 20 10 0 -10
f1 (ppm)

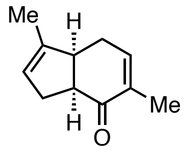

41

^{1}H NMR (400 M, $CDCl_3$)

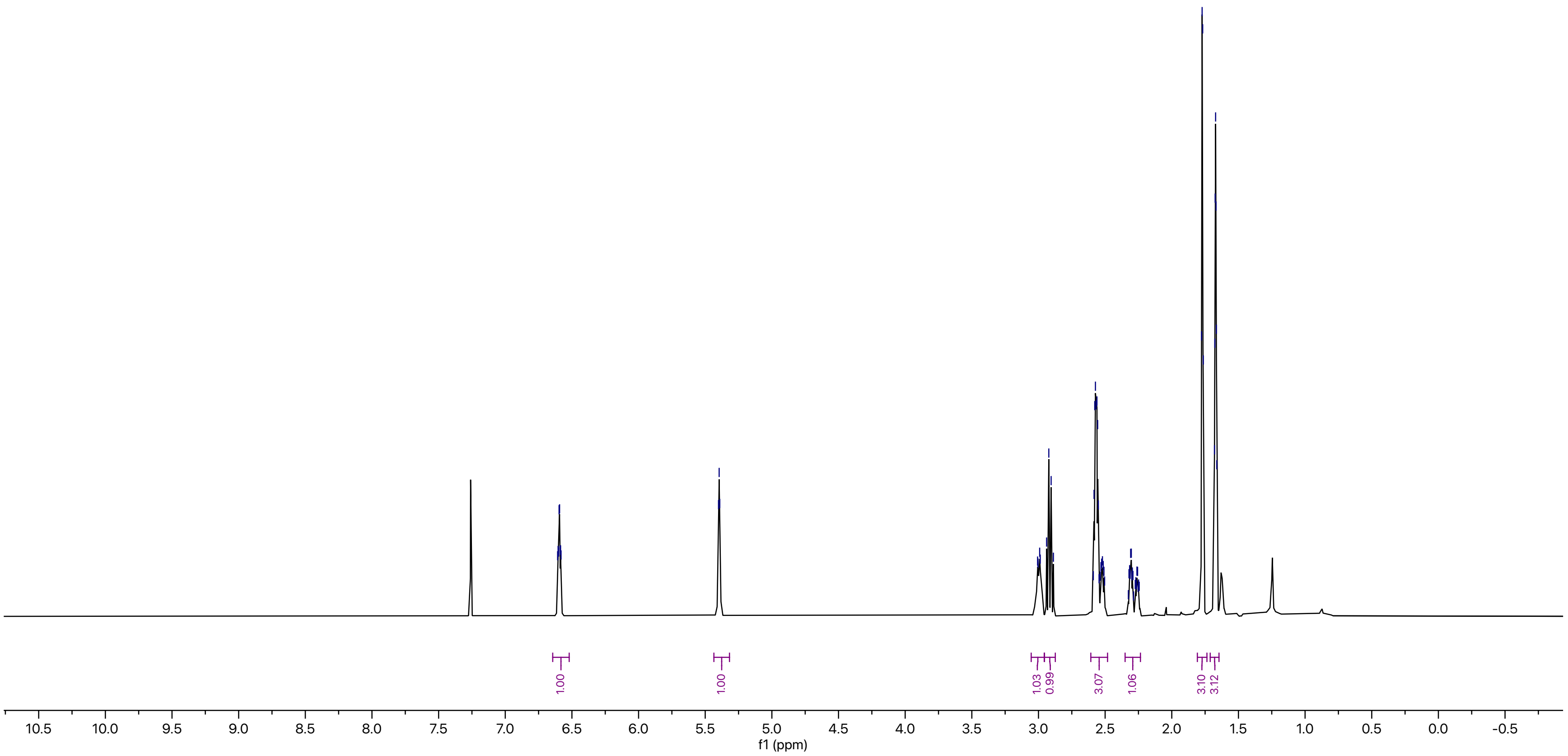

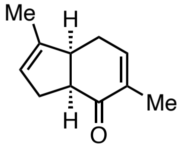

41

^{13}C NMR (100 M, $CDCl_3$)

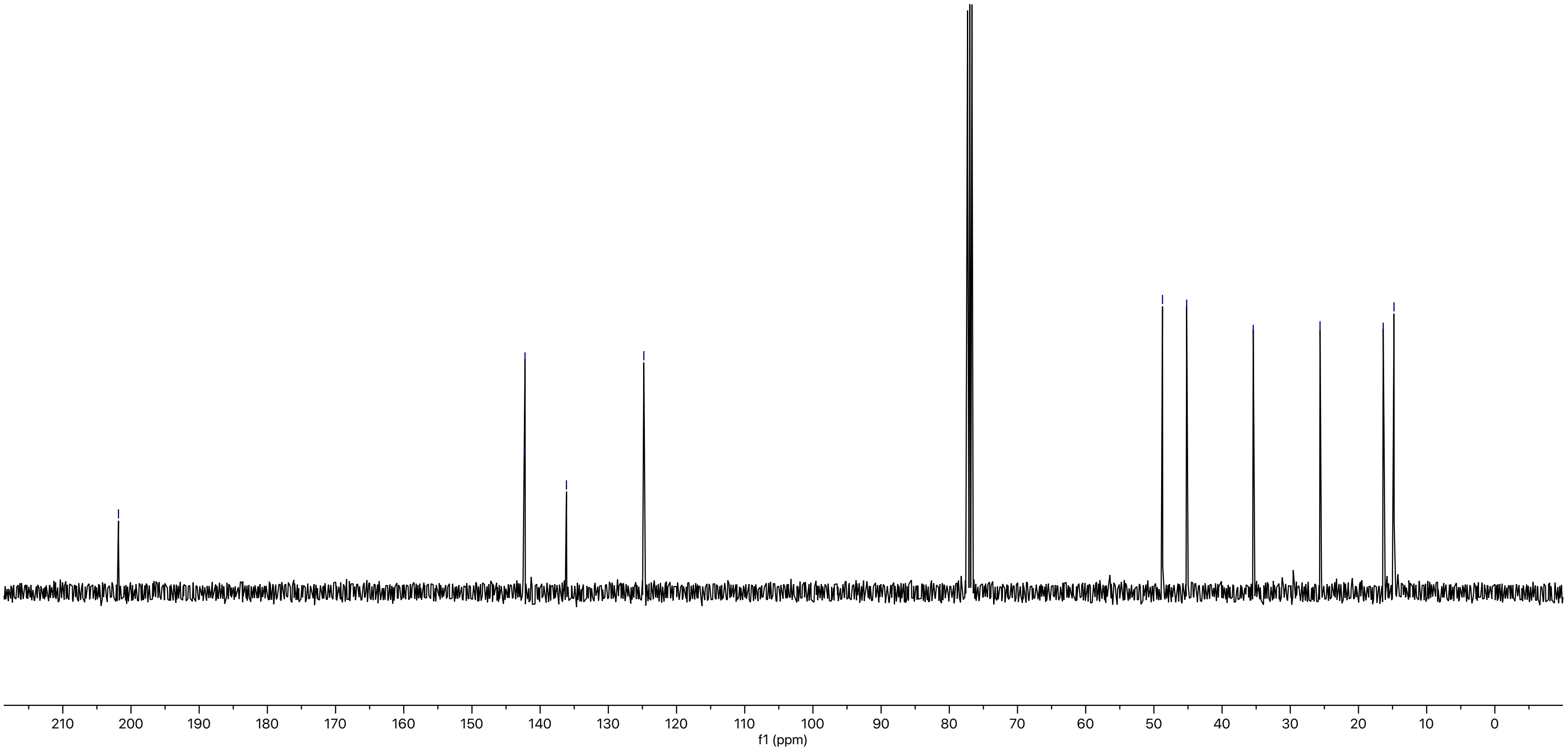

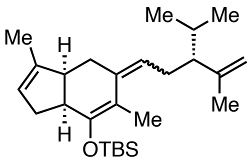

48 (E/Z = 6.2 / 1)

^{1}H NMR (400 M, $CDCl_3$)

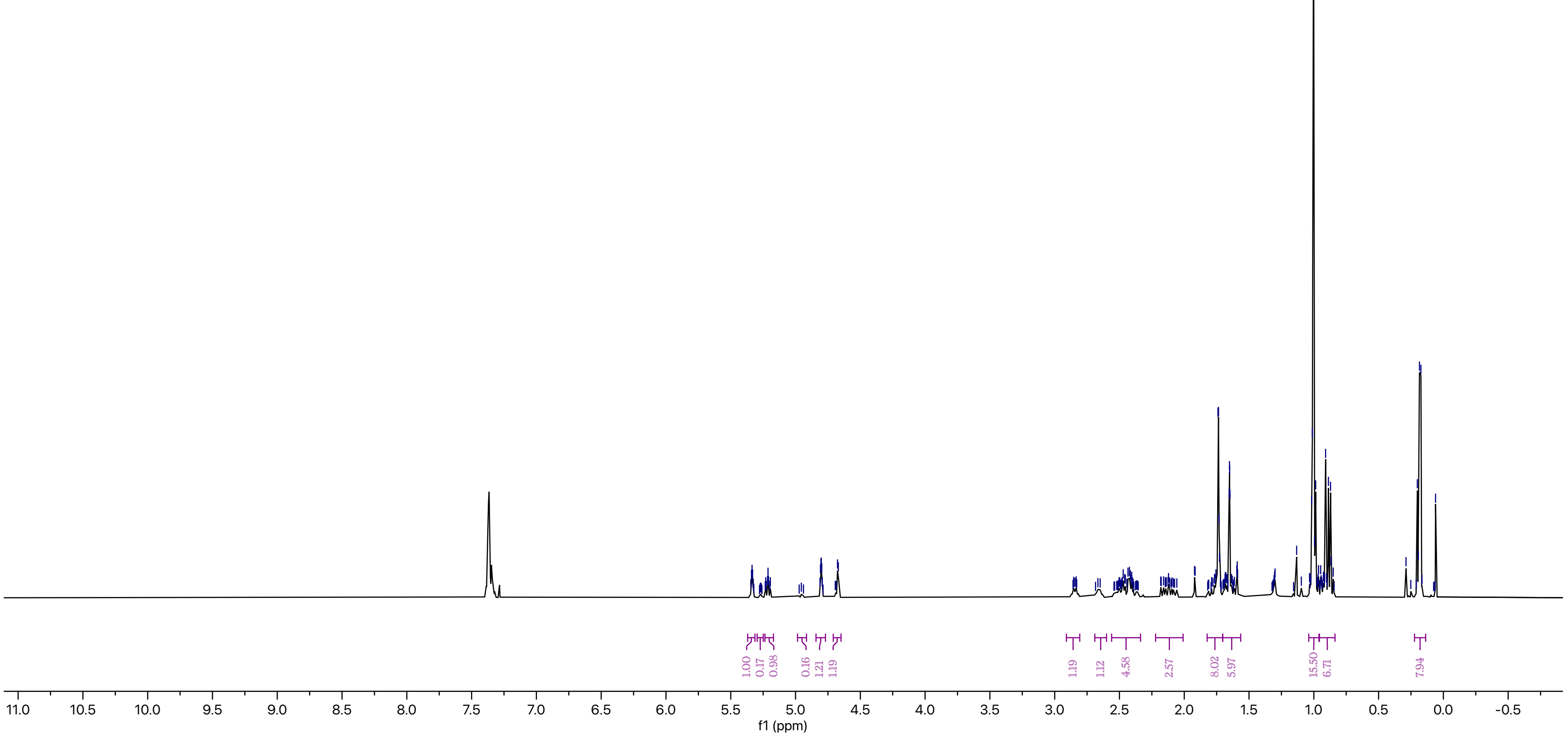

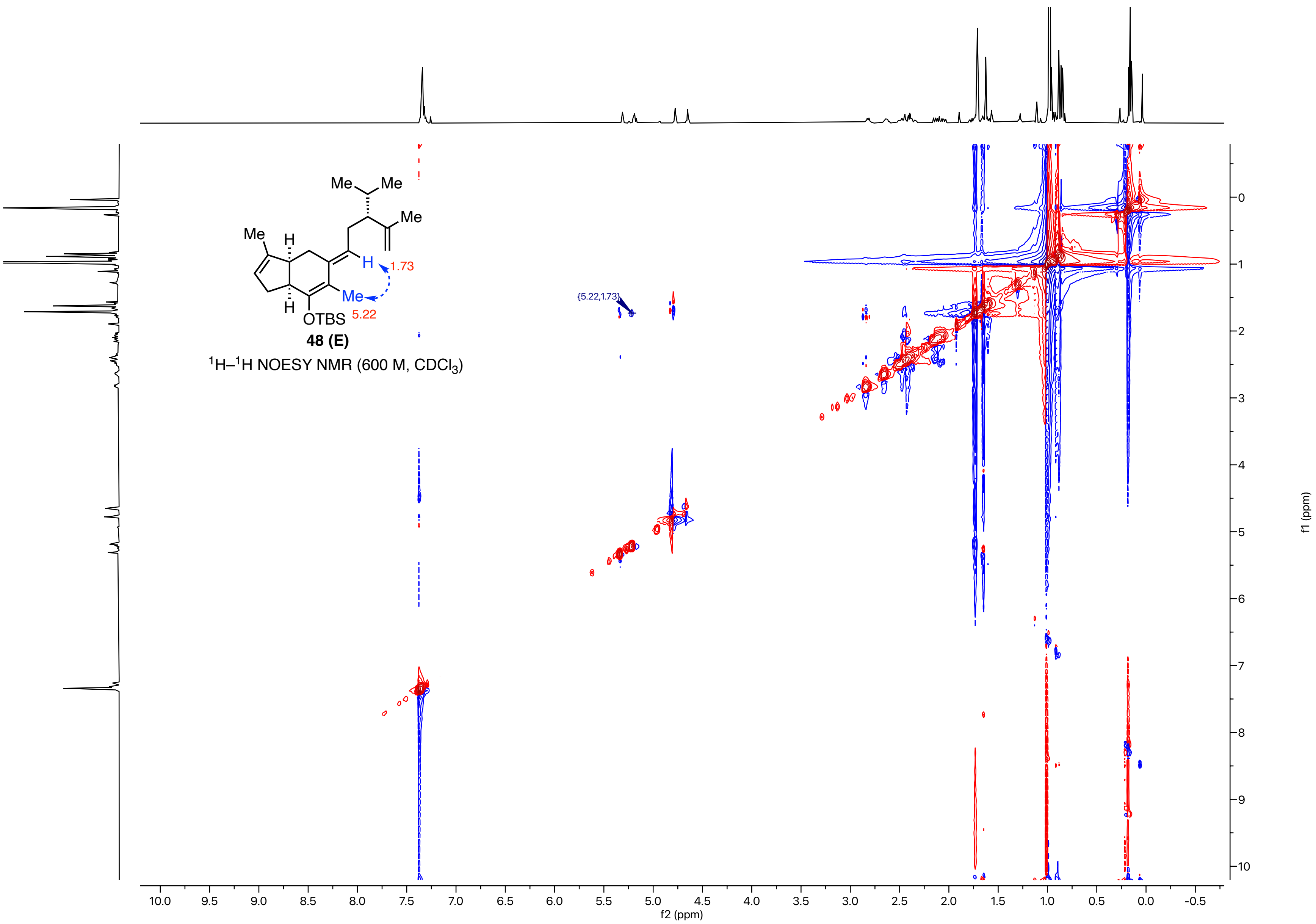
Me
Me
Me
Me
H
H
H
1.73
Me
5.22
OTBS
48 (E)
^{1}H–^{1}H NOESY NMR (600 M, CDCl$_3$)
{5.22,1.73}
f2 (ppm)
f1 (ppm)

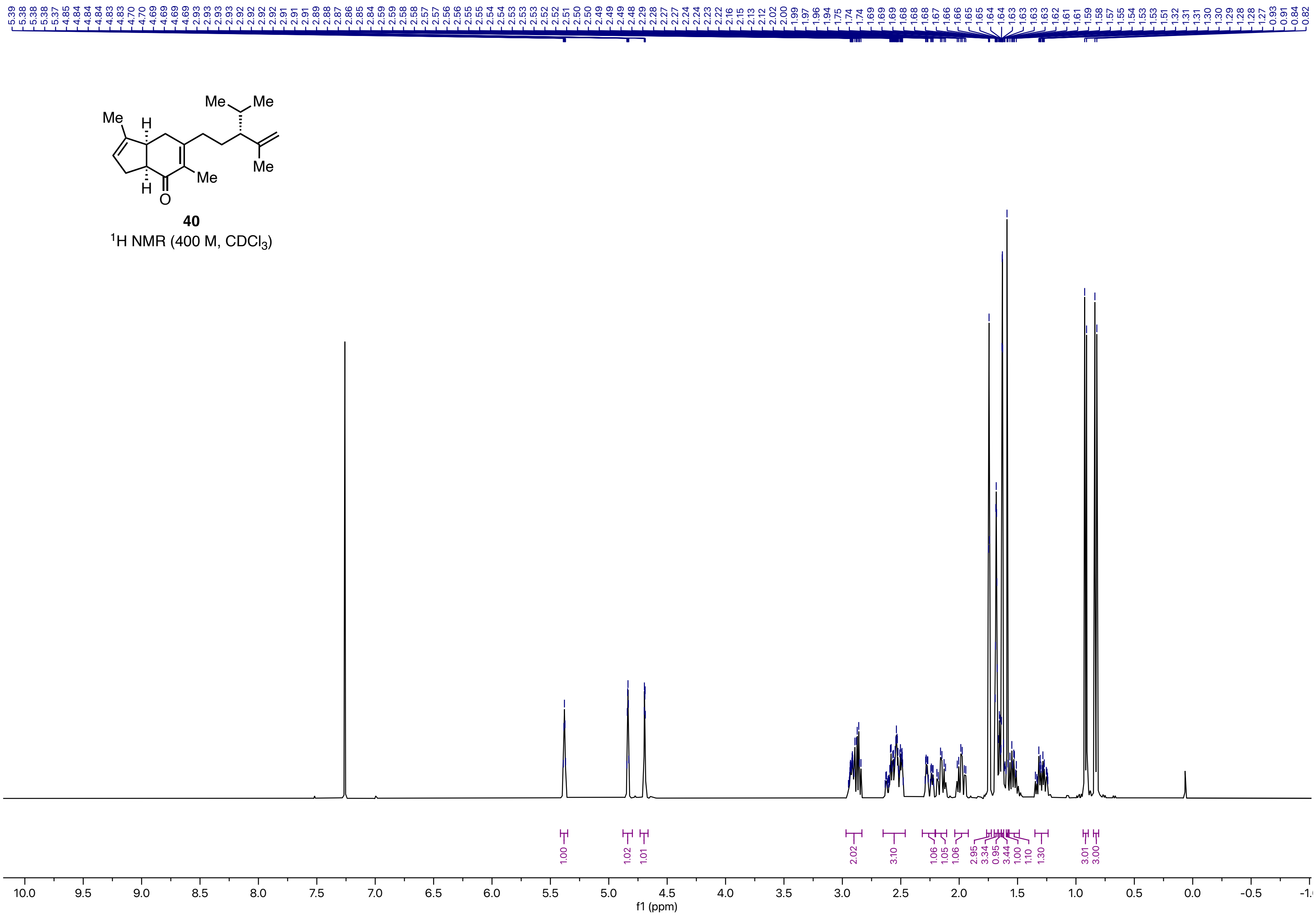

40
^{1}H NMR (400 M, CDCl$_3$)
f1 (ppm)

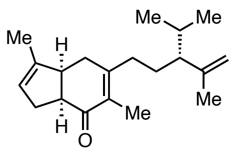

40

^{13}C NMR (100 M, $CDCl_3$)

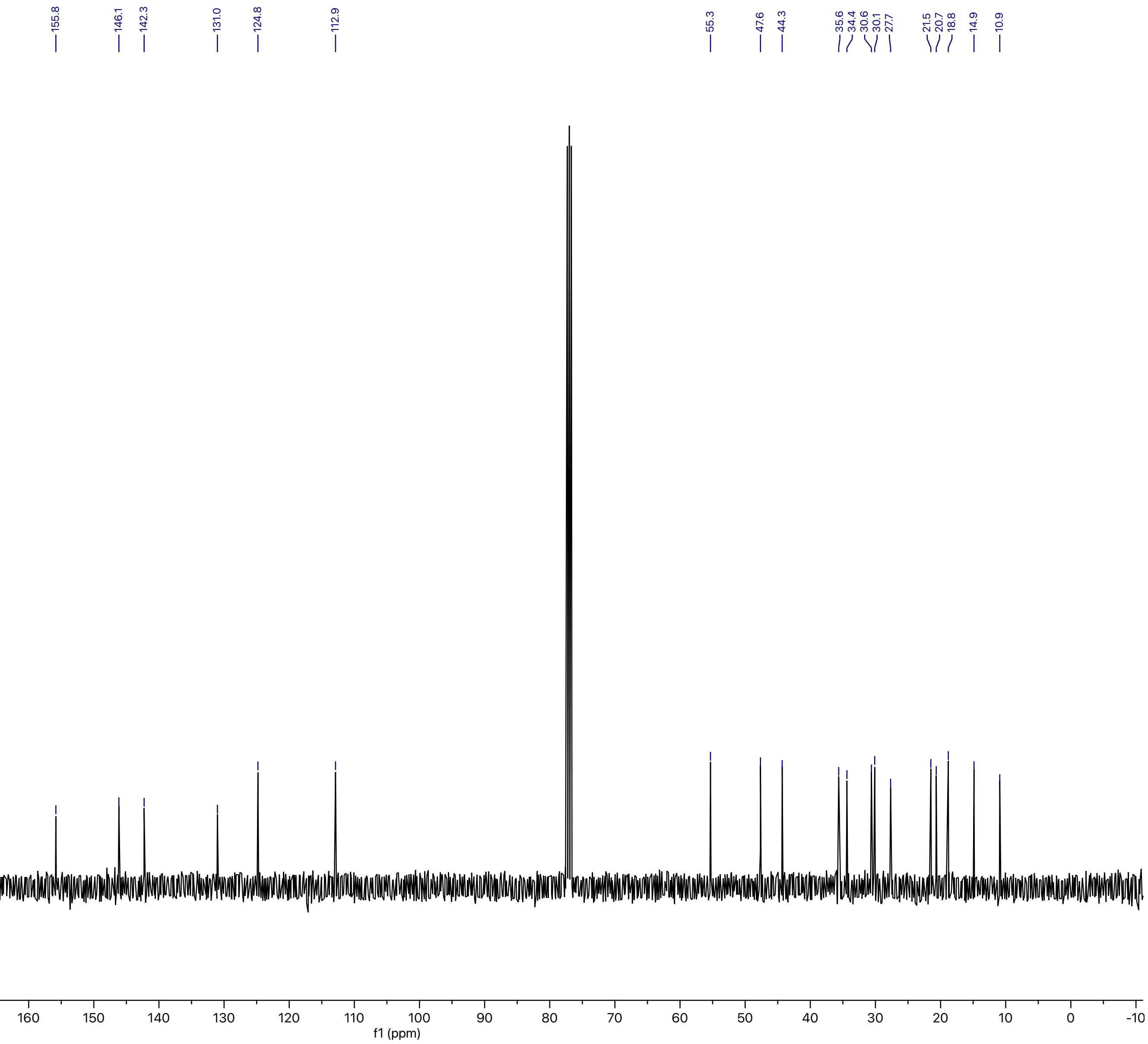

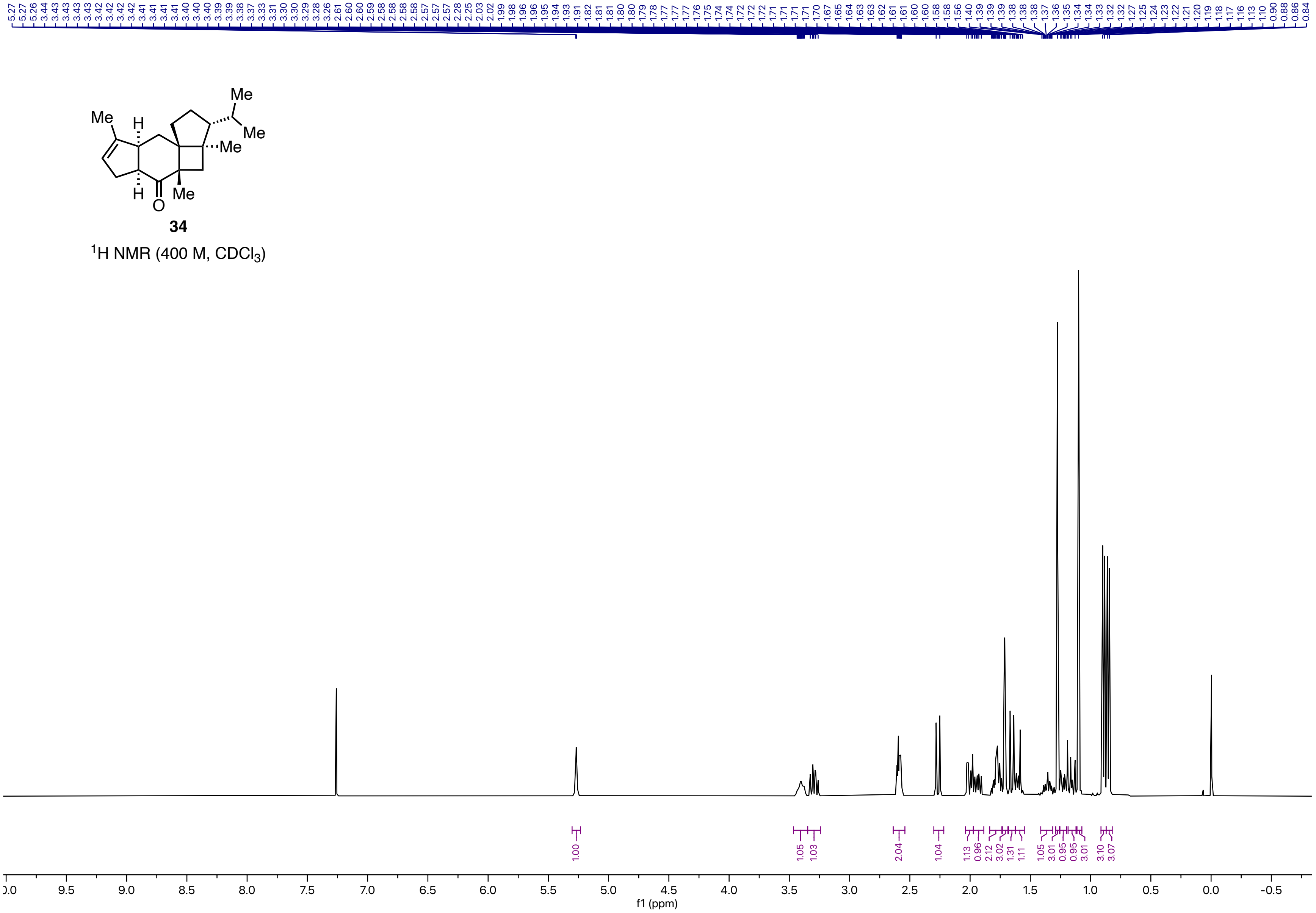

Me
H
Me
Me
Me
H
O
Me
34
^{1}H NMR (400 M, $CDCl_3$)
5.27 5.27 5.26 3.44 3.43 3.43 3.43 3.42 3.42 3.42 3.41 3.41 3.41 3.40 3.40 3.39 3.39 3.38 3.37 3.33 3.31 3.30 3.30 3.29 3.28 3.26 2.61 2.60 2.60 2.59 2.58 2.58 2.58 2.58 2.57 2.57 2.57 2.28 2.25 2.03 2.02 1.99 1.98 1.96 1.96 1.95 1.94 1.93 1.91 1.82 1.81 1.81 1.80 1.79 1.78 1.77 1.77 1.77 1.76 1.75 1.74 1.74 1.72 1.72 1.72 1.71 1.71 1.71 1.70 1.67 1.65 1.64 1.63 1.63 1.62 1.61 1.61 1.60 1.60 1.58 1.58 1.56 1.40 1.39 1.39 1.39 1.38 1.38 1.38 1.37 1.36 1.35 1.34 1.34 1.33 1.32 1.32 1.27 1.25 1.24 1.23 1.22 1.21 1.20 1.19 1.18 1.17 1.16 1.13 1.10 0.90 0.88 0.86 0.84
1.00
1.05
1.03
2.04
1.04
1.13
0.96
2.12
3.02
1.31
1.11
1.05
3.01
0.95
0.95
3.01
3.10
3.07
10.0
9.5
9.0
8.5
8.0
7.5
7.0
6.5
6.0
5.5
5.0
4.5
4.0
3.5
3.0
2.5
2.0
1.5
1.0
0.5
0.0
-0.5
f1 (ppm)

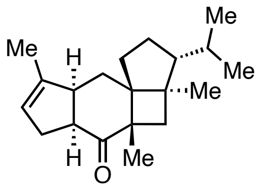

34

^{13}C NMR (100 M, $CDCl_3$)

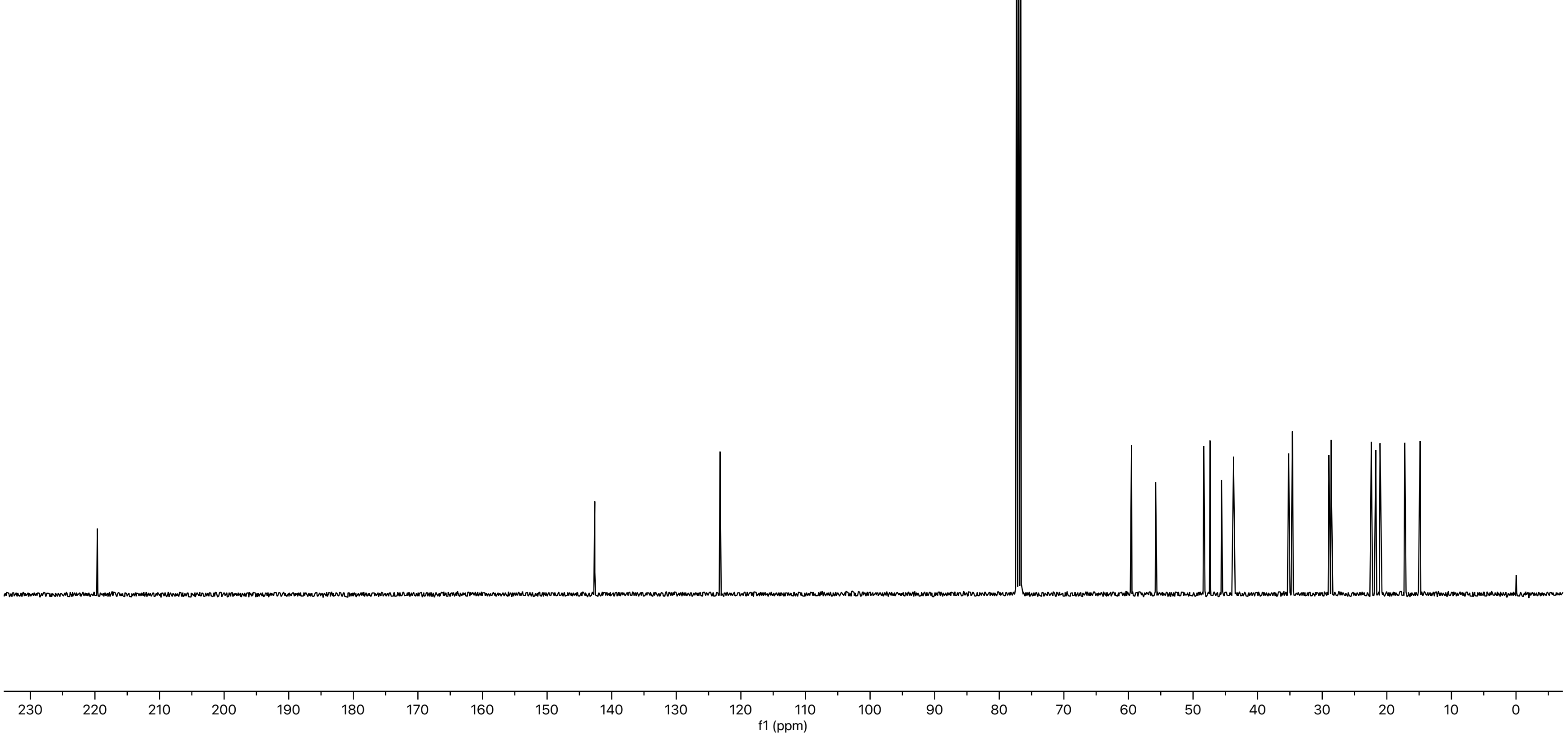

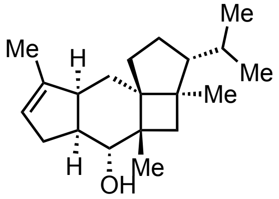

34a

^{1}H NMR (400 M, $CDCl_3$)

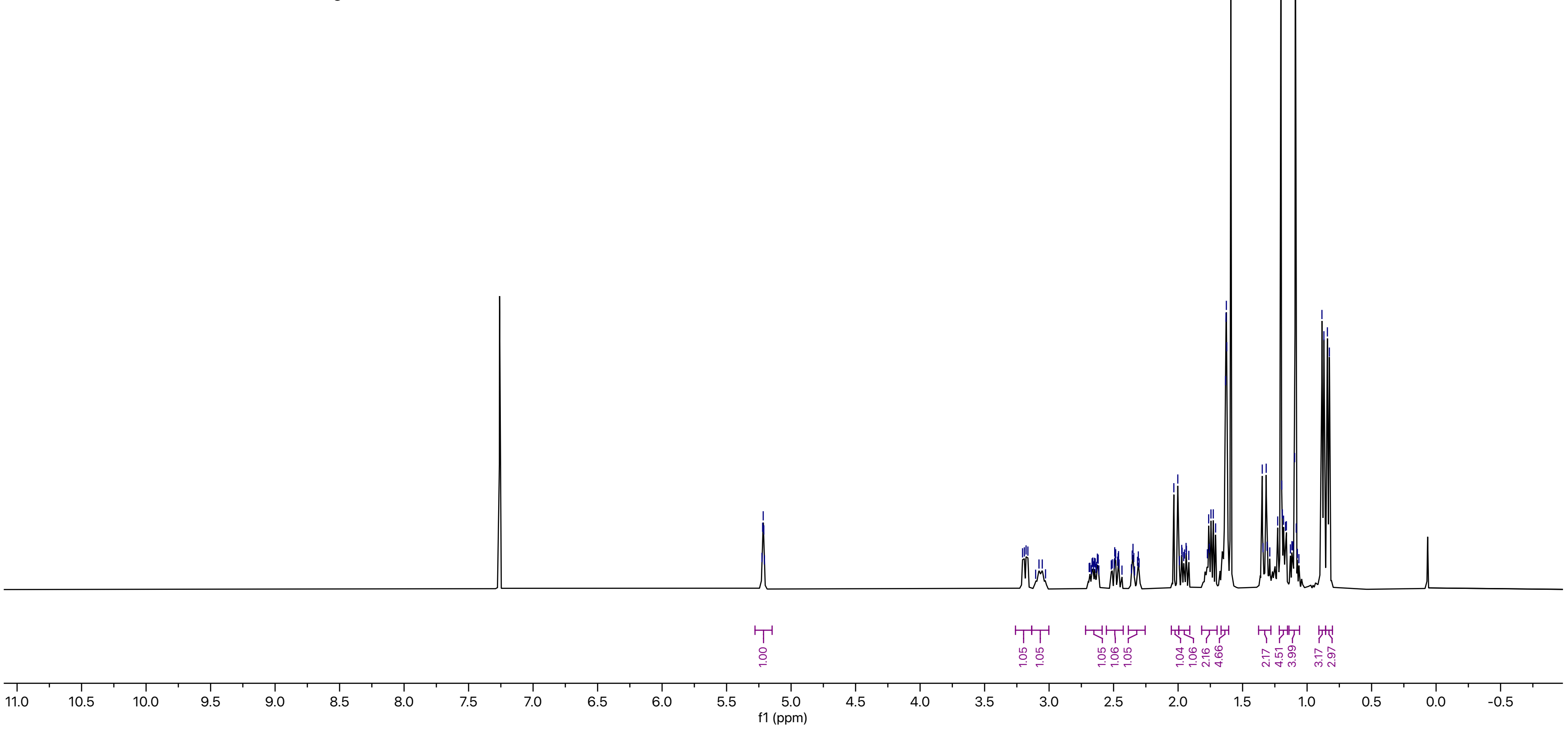

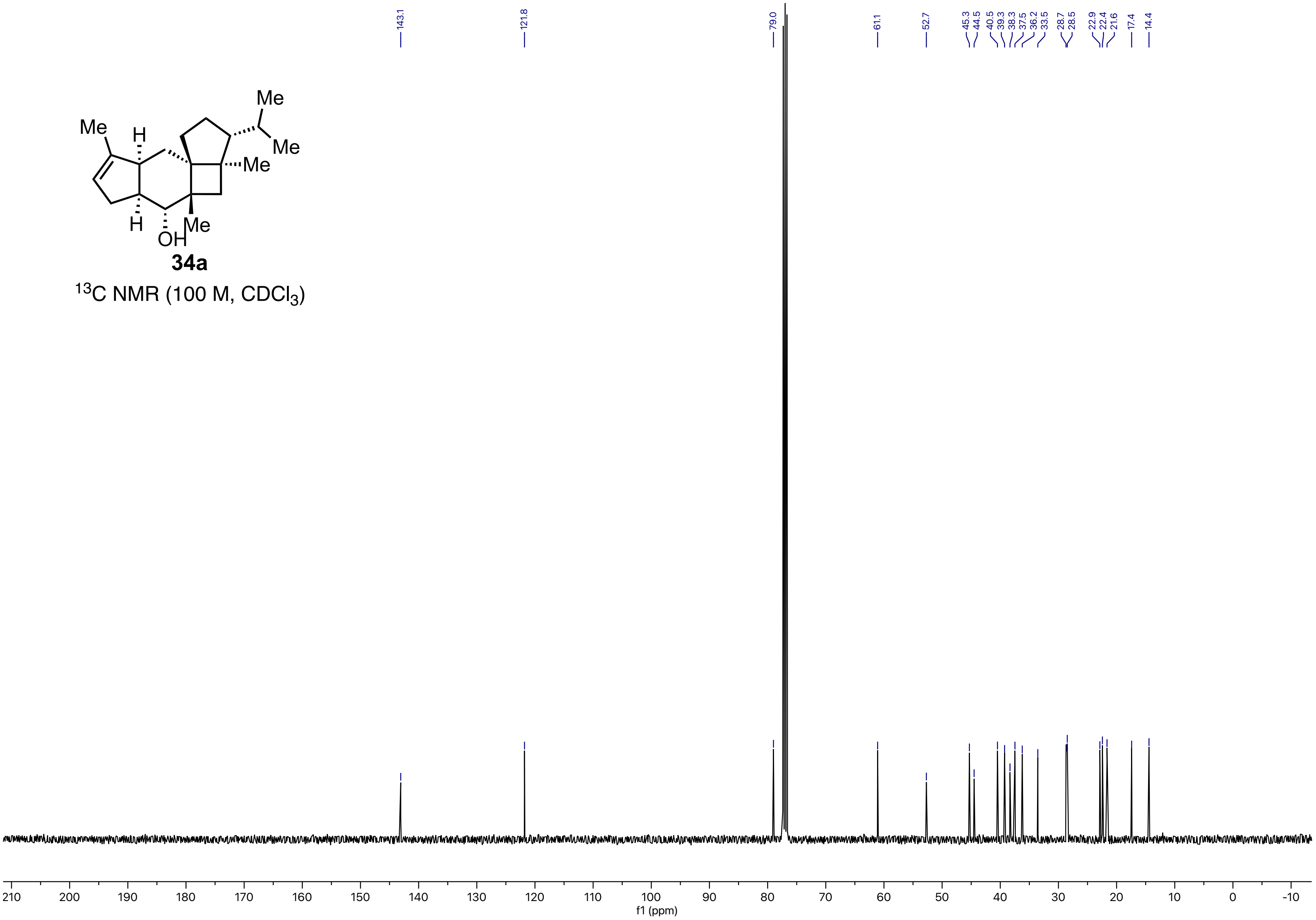

34a

^{13}C NMR (100 M, $CDCl_3$)

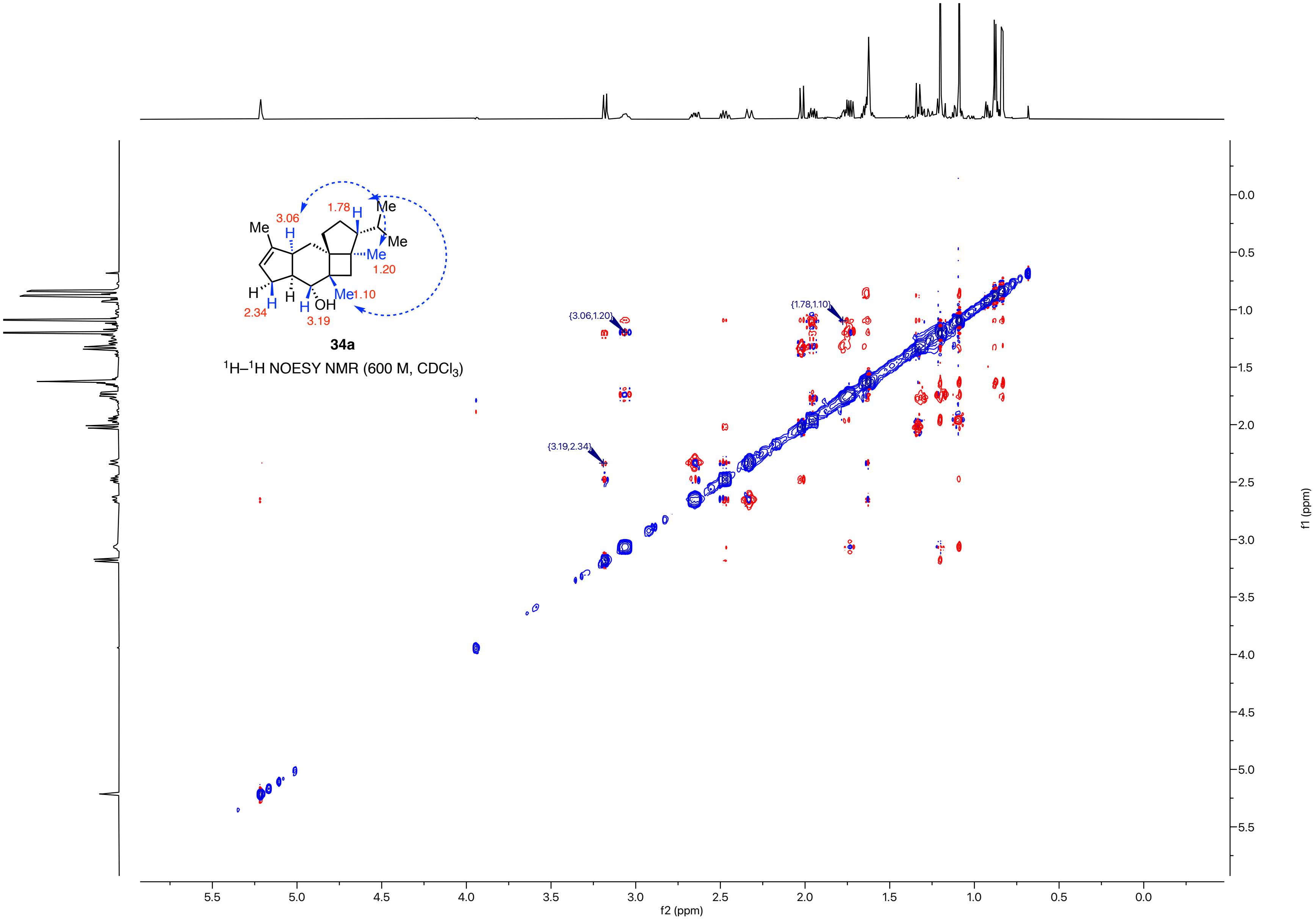
Me
3.06
1.78
H
Me
Me
Me
1.20
H
H
H
H
OH
Me1.10
2.34
3.19
34a
1H–1H NOESY NMR (600 M, CDCl3)
{3.06,1.20}
{1.78,1.10}
{3.19,2.34}
f2 (ppm)
f1 (ppm)

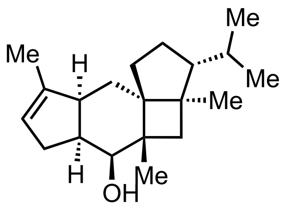

34b

^{1}H NMR (400 M, $CDCl_3$)

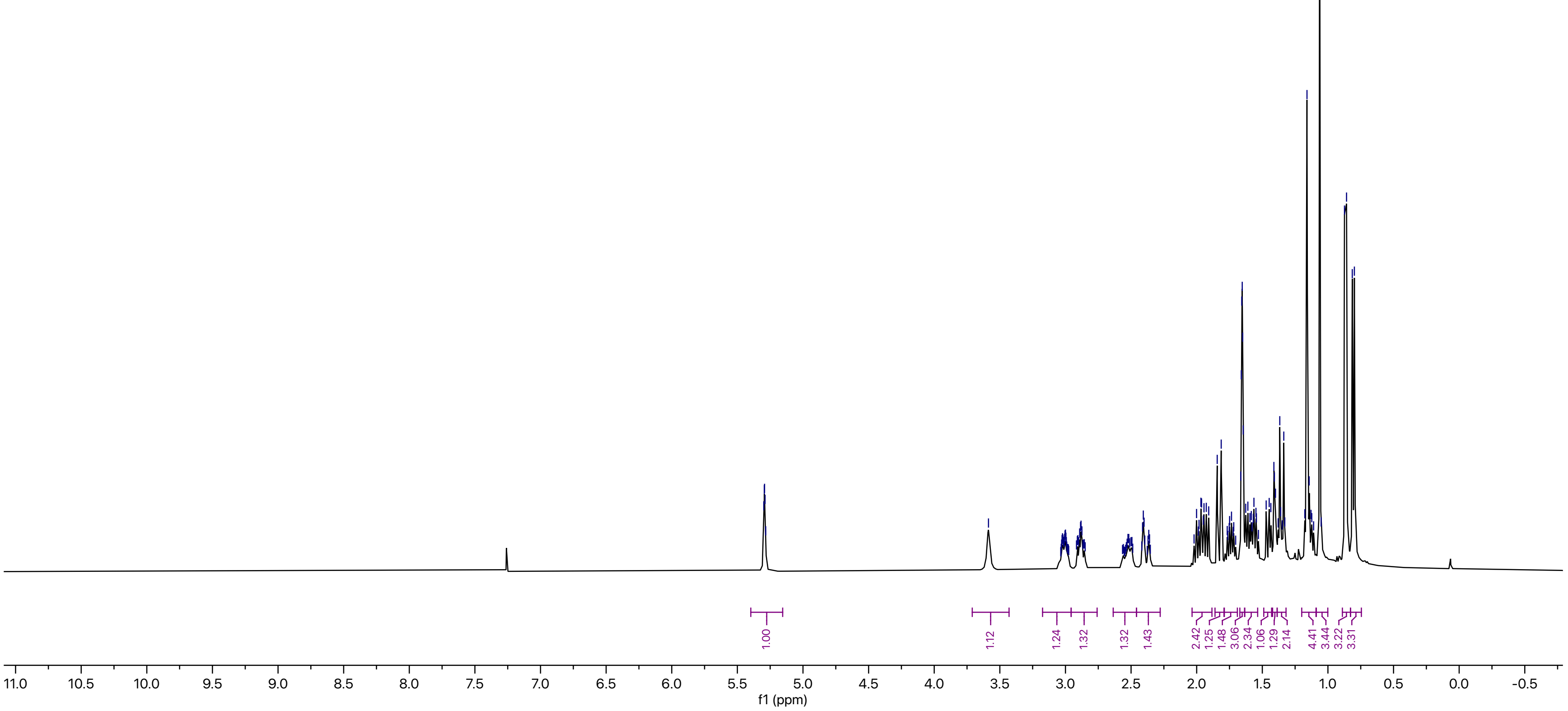

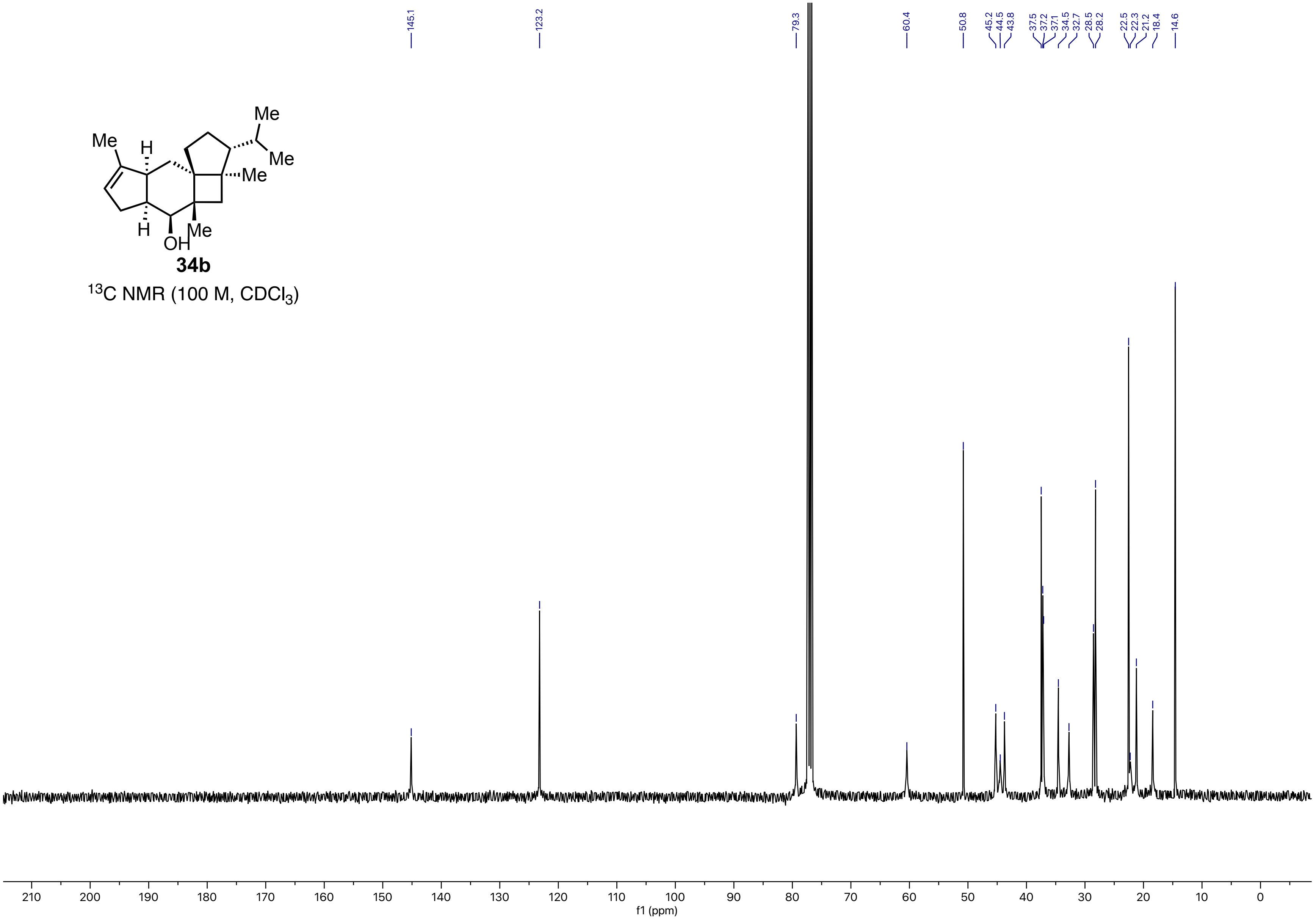
145.1
123.2
79.3
60.4
50.8
45.2
44.5
43.8
37.5
37.2
37.1
34.5
32.7
28.5
28.2
22.5
22.3
21.2
18.4
14.6
Me
Me
H
Me
Me
Me
H
OH
Me
34b
13C NMR (100 M, CDCl3)
210 200 190 180 170 160 150 140 130 120 110 100 90 80 70 60 50 40 30 20 10 0
f1 (ppm)

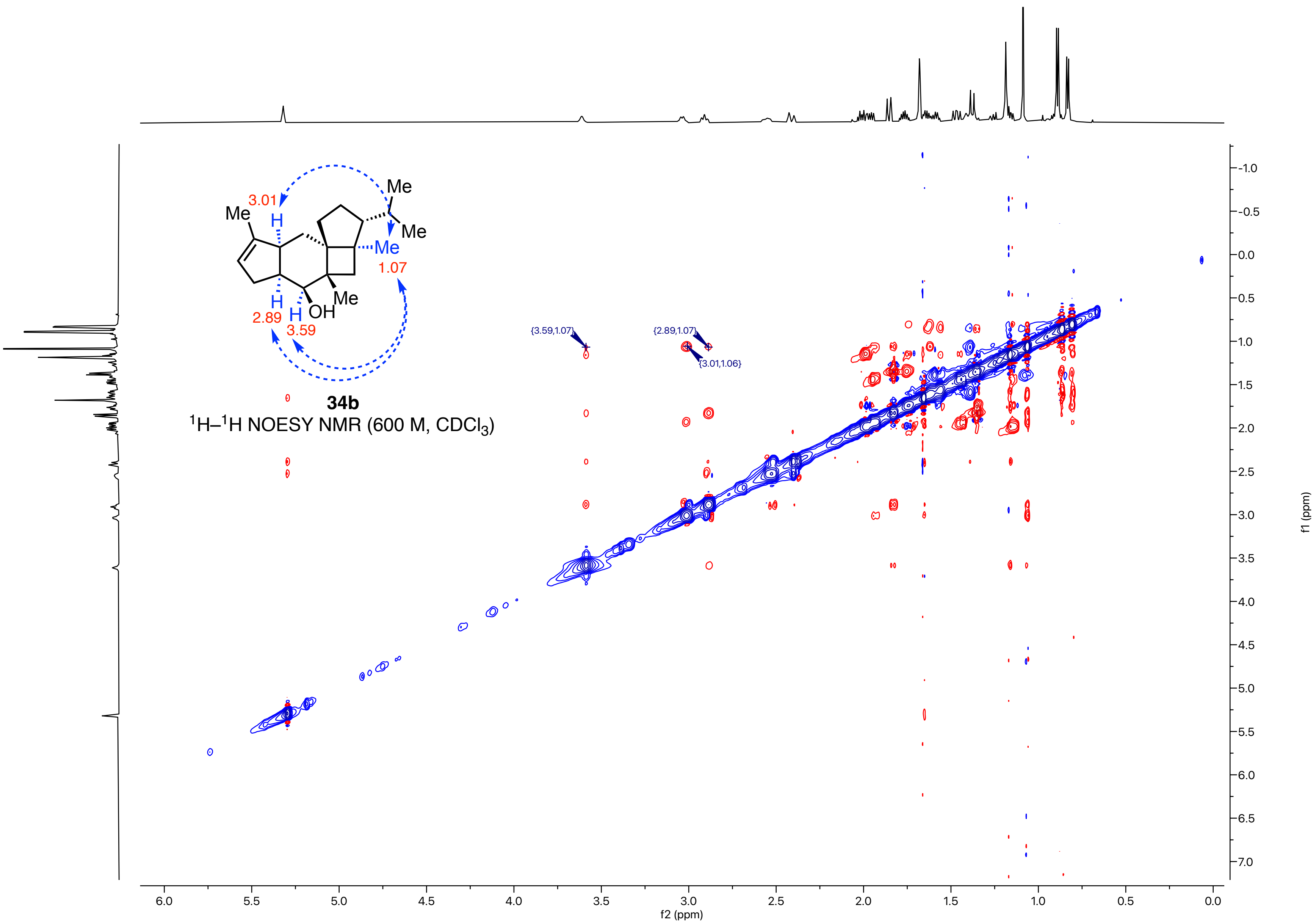
Me
3.01
H
Me
Me
Me
1.07
Me
H
H
OH
2.89
3.59
34b
1H–1H NOESY NMR (600 M, CDCl3)
{3.59,1.07}
{2.89,1.07}
{3.01,1.06}
f2 (ppm)
f1 (ppm)

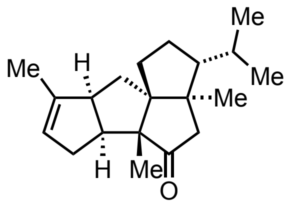

33

^{1}H NMR (400 M, $CDCl_3$)

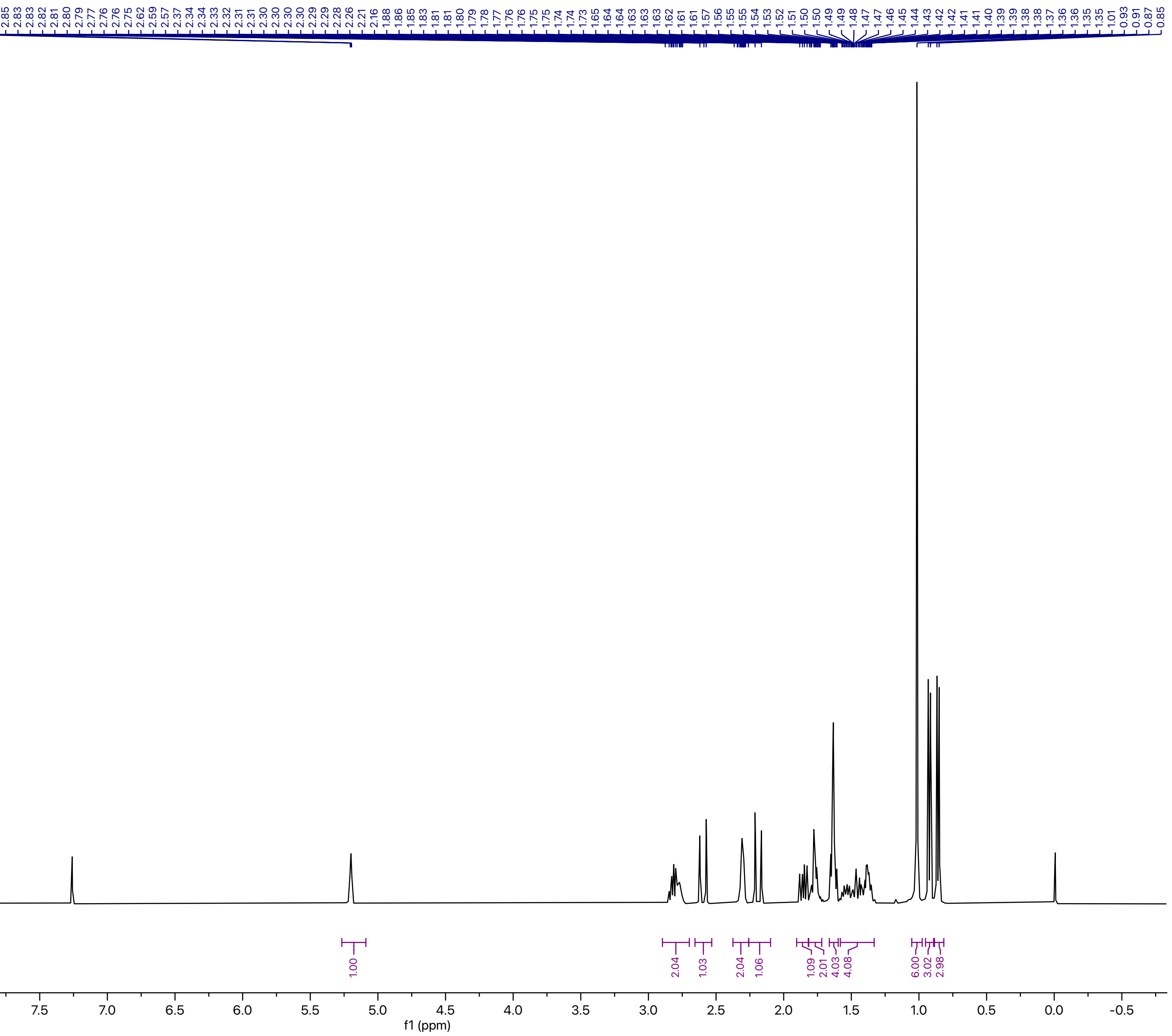

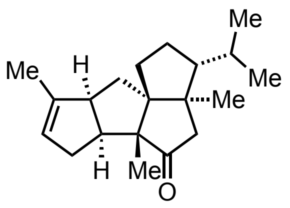

33

^{13}C NMR (100 M, $CDCl_3$)

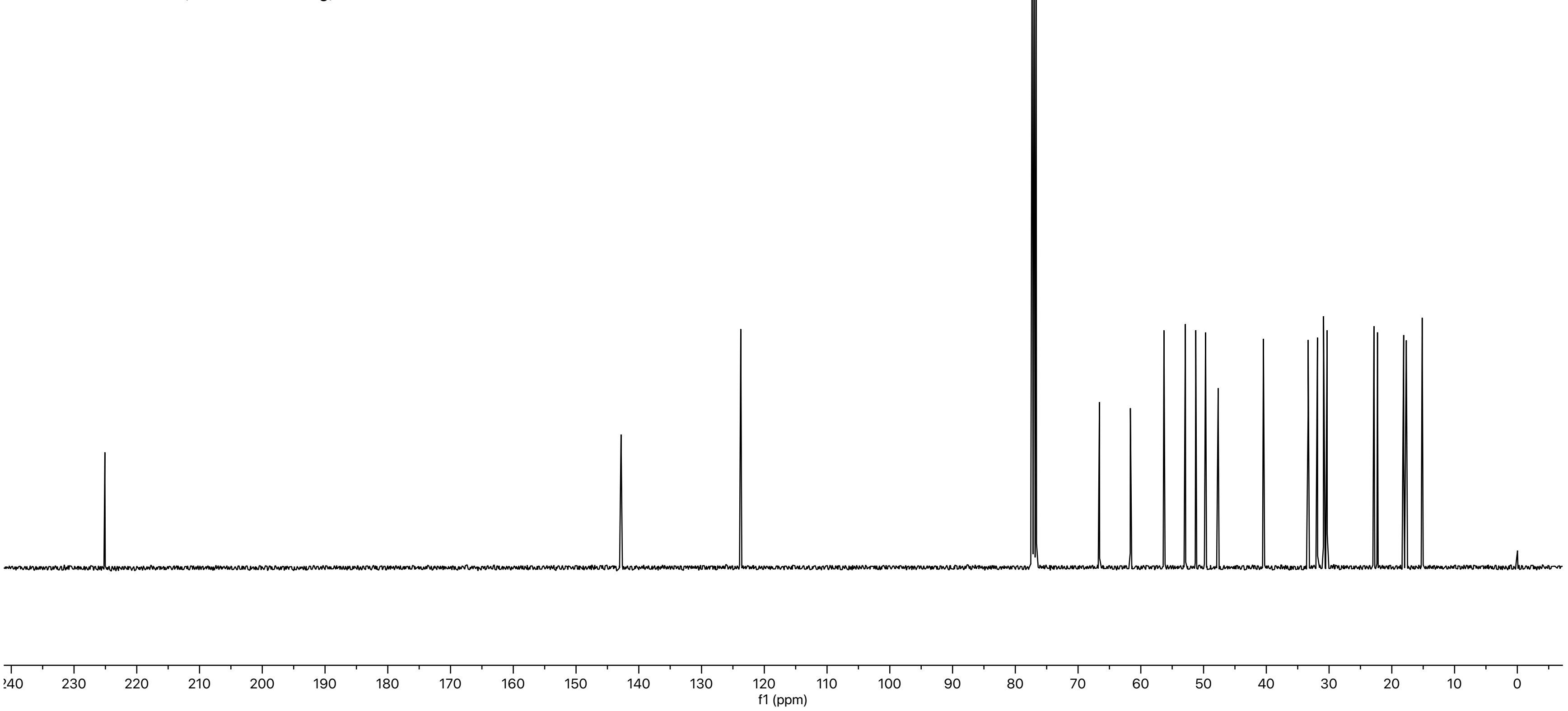

33a

^{1}H NMR (400 M, CDCl$_3$)

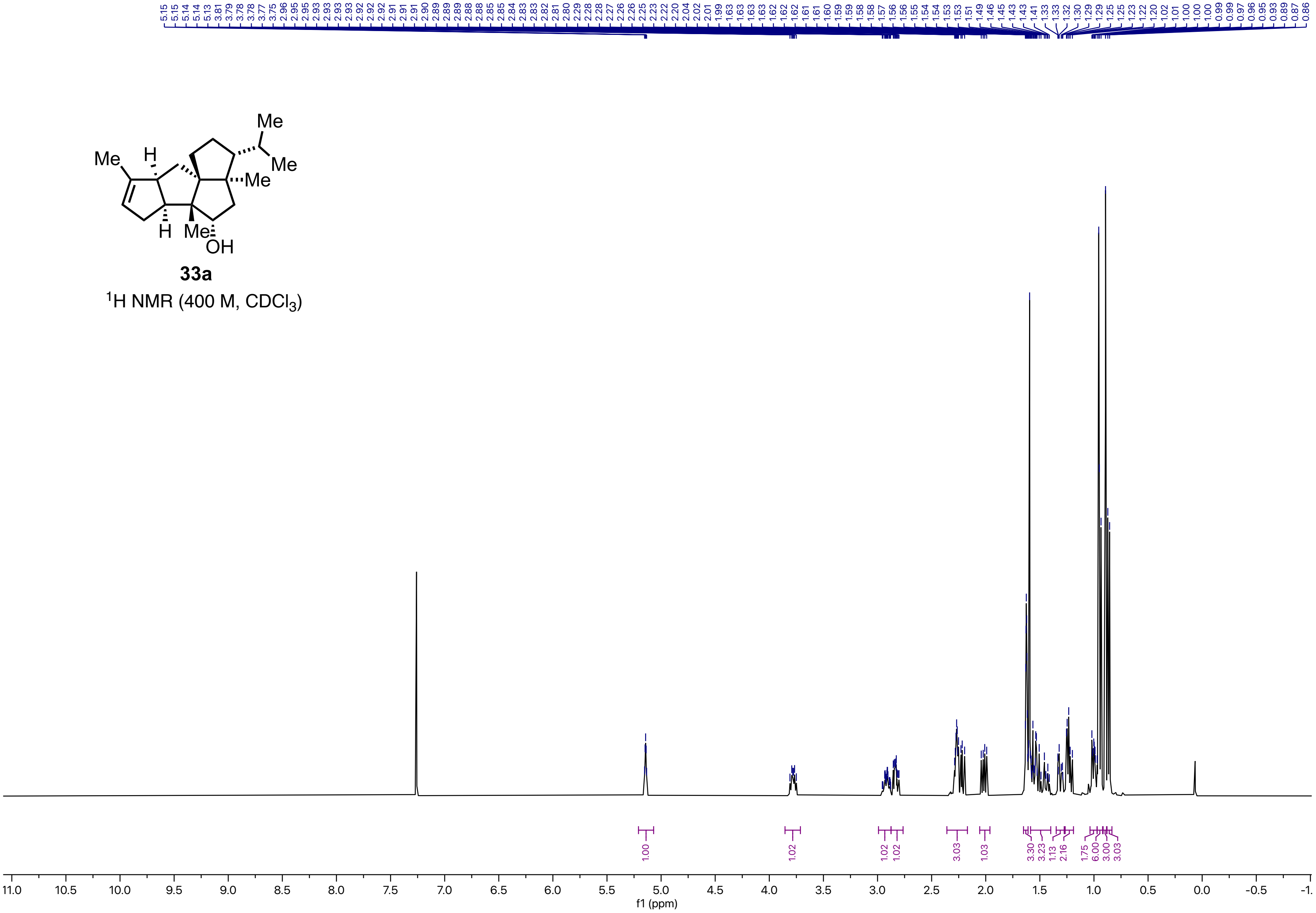

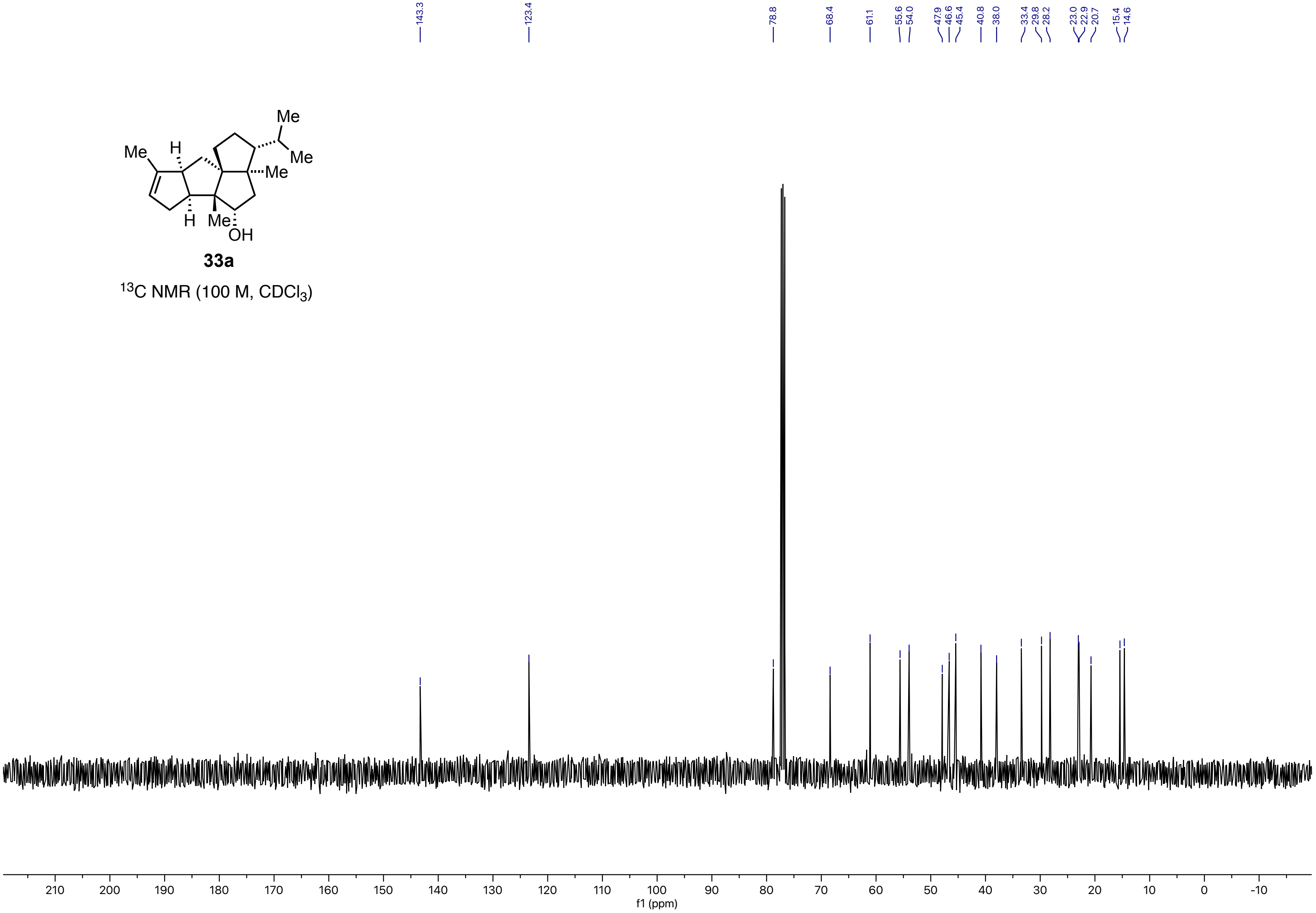

33a

^{13}C NMR (100 M, $CDCl_3$)

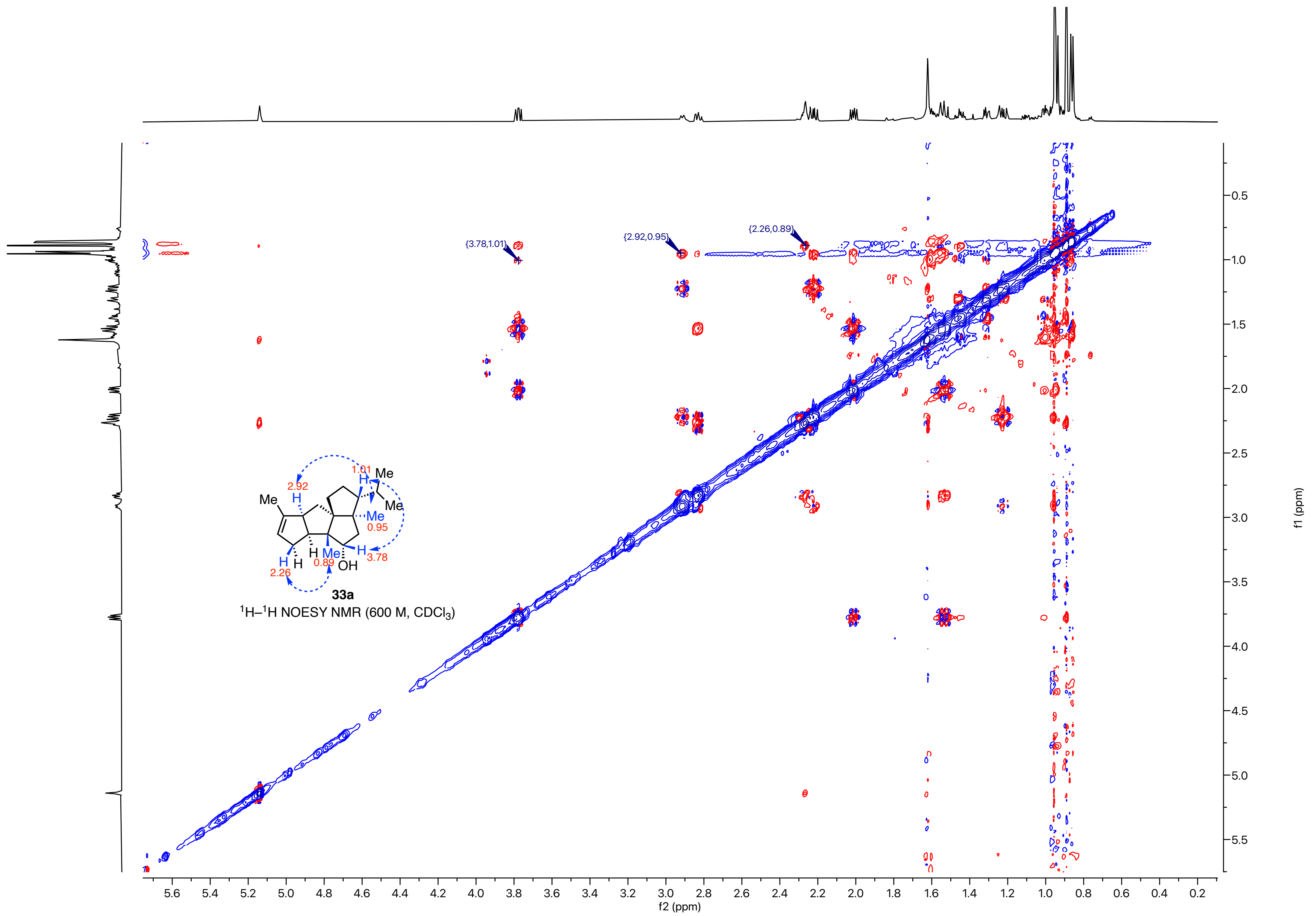

{3.78,1.01}
{2.92,0.95}
{2.26,0.89}
Me
Me
Me
Me
H
H
H
H
H
OH
2.92
1.01
0.95
3.78
0.89
2.26
33a
^{1}H–^{1}H NOESY NMR (600 M, $CDCl_3$)
f2 (ppm)
f1 (ppm)

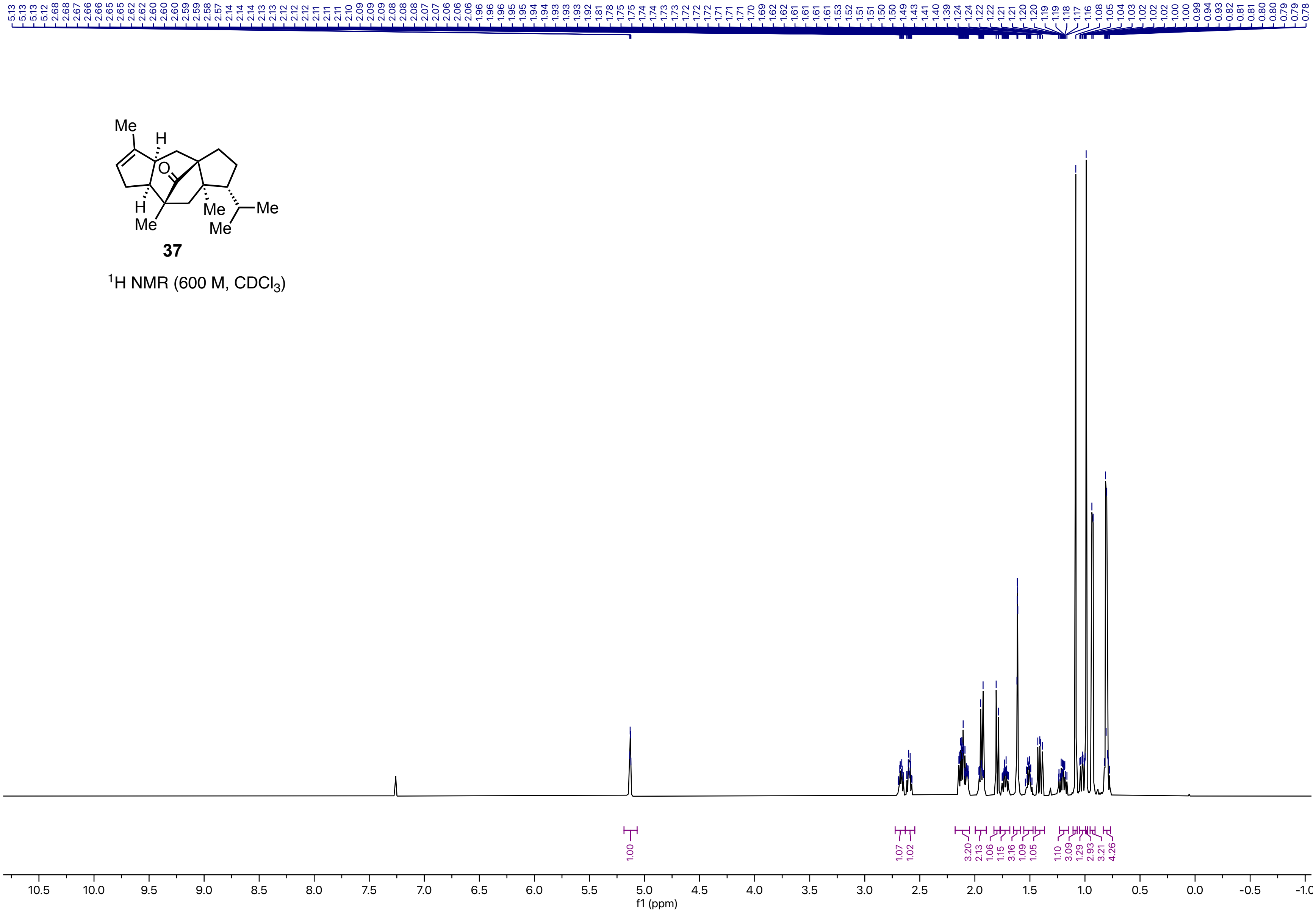
5.13 5.13 5.13 5.12 2.68 2.68 2.67 2.66 2.66 2.65 2.62 2.60 2.60 2.59 2.59 2.58 2.57 2.14 2.14 2.14 2.13 2.13 2.12 2.12 2.11 2.11 2.10 2.09 2.09 2.08 2.08 2.08 2.07 2.06 2.06 2.06 1.96 1.96 1.95 1.94 1.93 1.93 1.92 1.81 1.78 1.75 1.74 1.74 1.73 1.72 1.72 1.72 1.71 1.71 1.71 1.70 1.69 1.62 1.61 1.61 1.61 1.53 1.52 1.51 1.51 1.50 1.49 1.43 1.41 1.40 1.39 1.24 1.24 1.22 1.22 1.21 1.21 1.20 1.20 1.19 1.19 1.18 1.17 1.16 1.08 1.05 1.04 1.03 1.02 1.02 1.00 0.99 0.94 0.93 0.82 0.81 0.81 0.80 0.80 0.79 0.79 0.78
Me
H
O
H
Me
Me
Me
Me
37
1H NMR (600 M, CDCl3)
1.00
1.07 1.02
3.20 2.13 1.06 1.15 3.16 1.09 1.05 1.10 3.09 1.29 2.93 3.21 4.26
10.5 10.0 9.5 9.0 8.5 8.0 7.5 7.0 6.5 6.0 5.5 5.0 4.5 4.0 3.5 3.0 2.5 2.0 1.5 1.0 0.5 0.0 -0.5 -1.0
f1 (ppm)

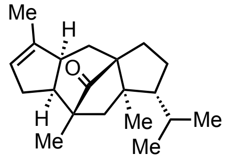

37

^{13}C NMR (150 M, $CDCl_3$)

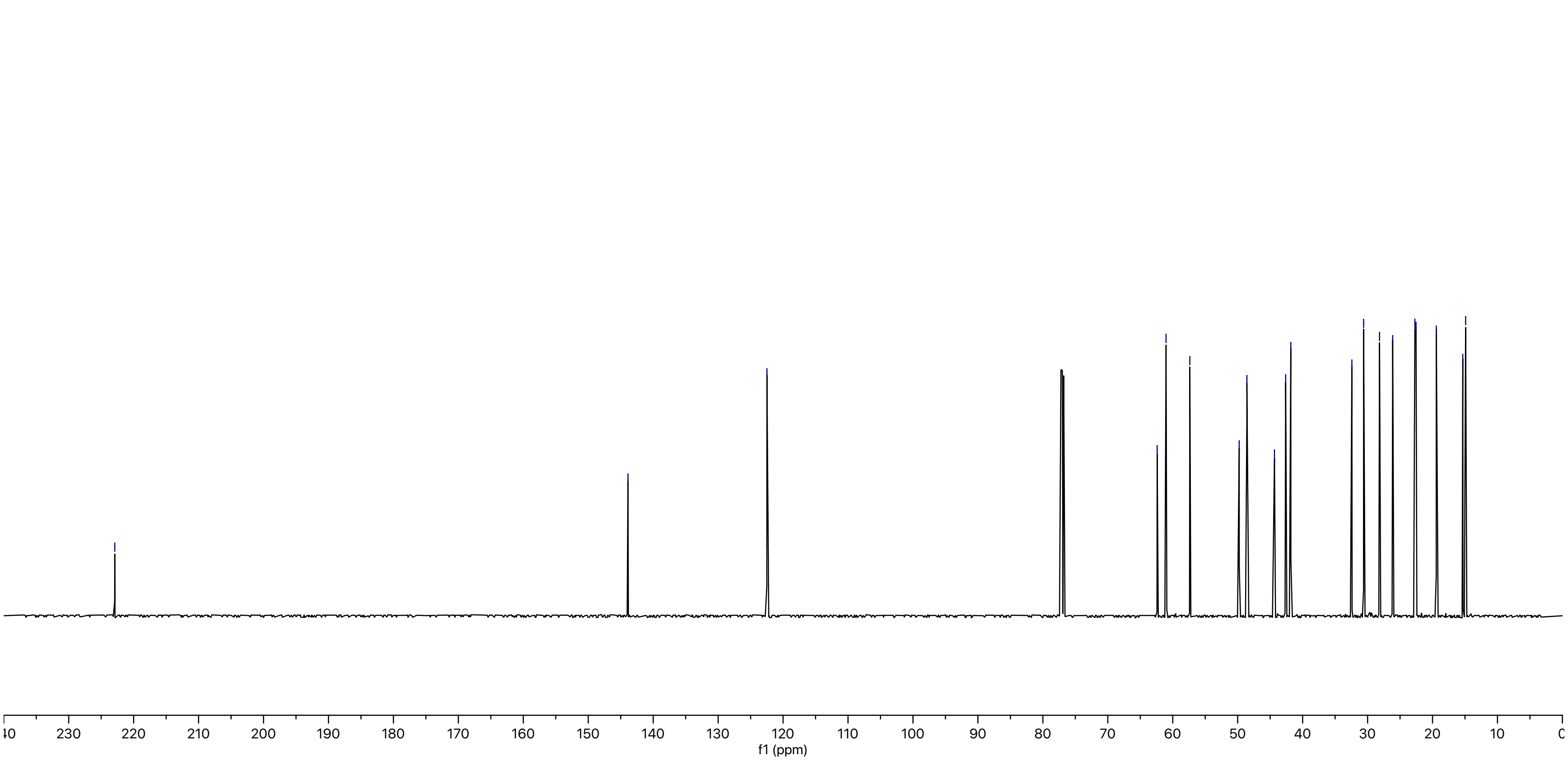

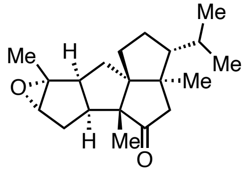

49

^1H NMR (400 M, $CDCl_3$)

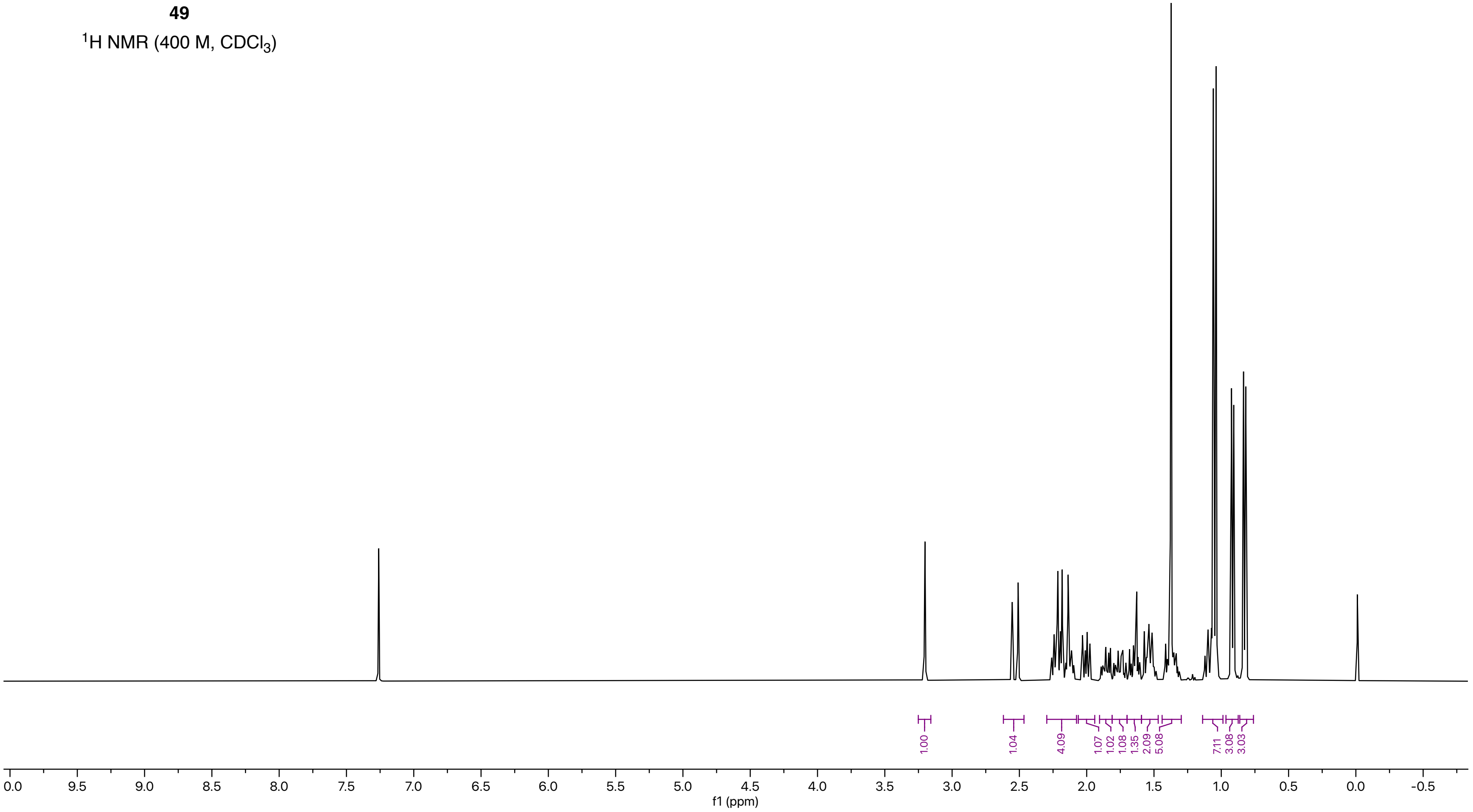

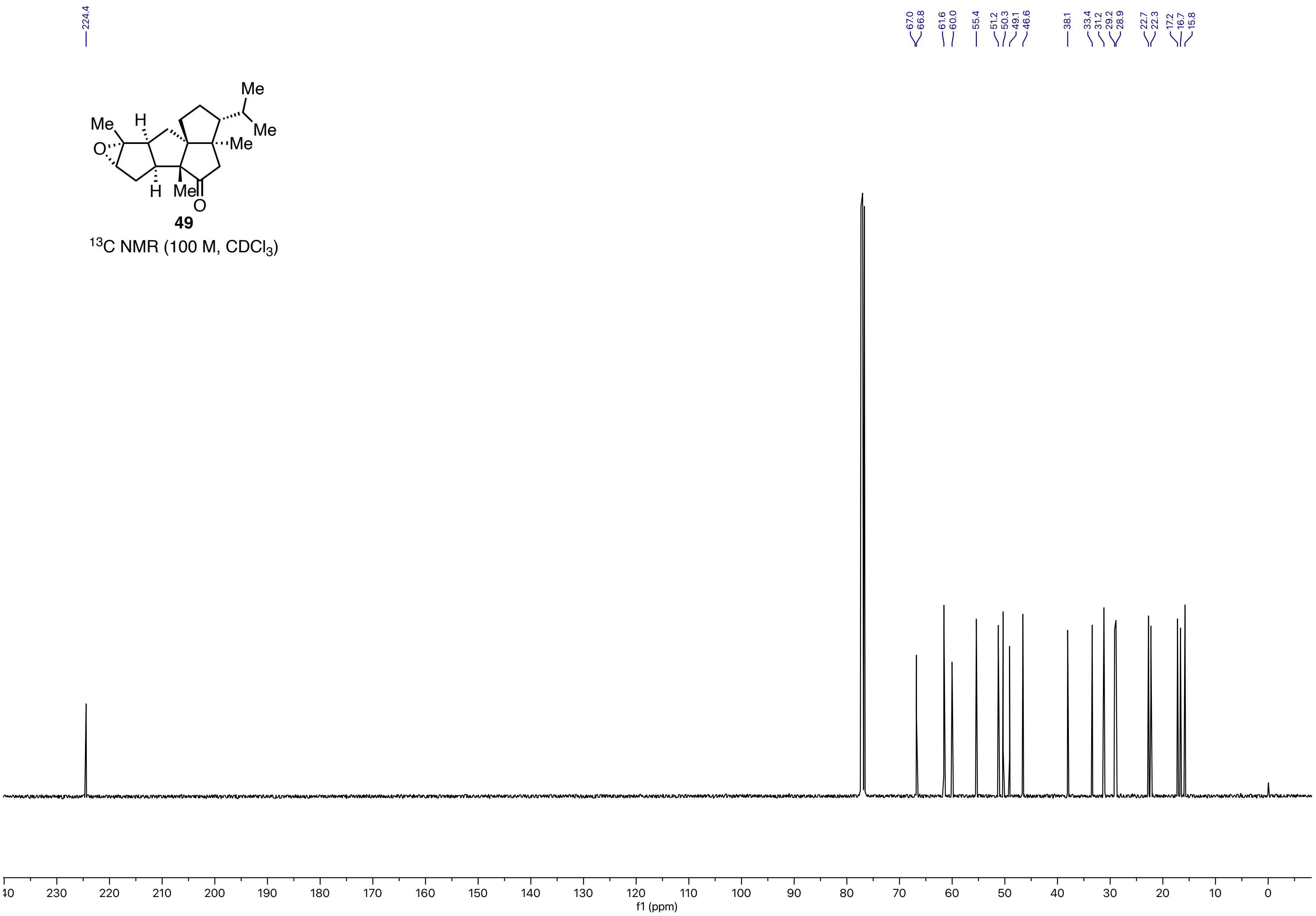

49

^{13}C NMR (100 M, $CDCl_3$)

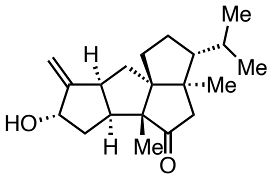

49a

^{1}H NMR (400 M, $CDCl_3$)

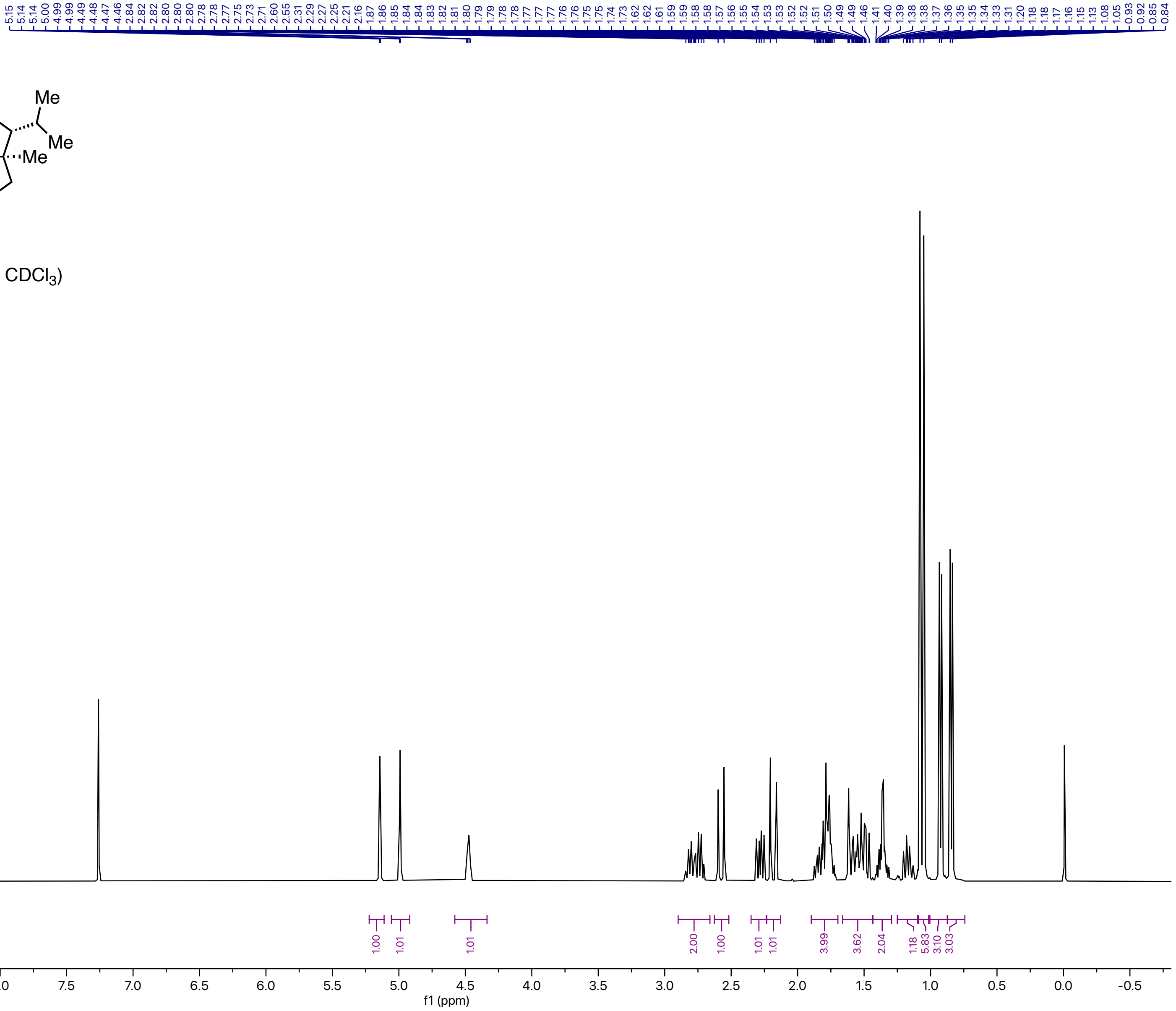

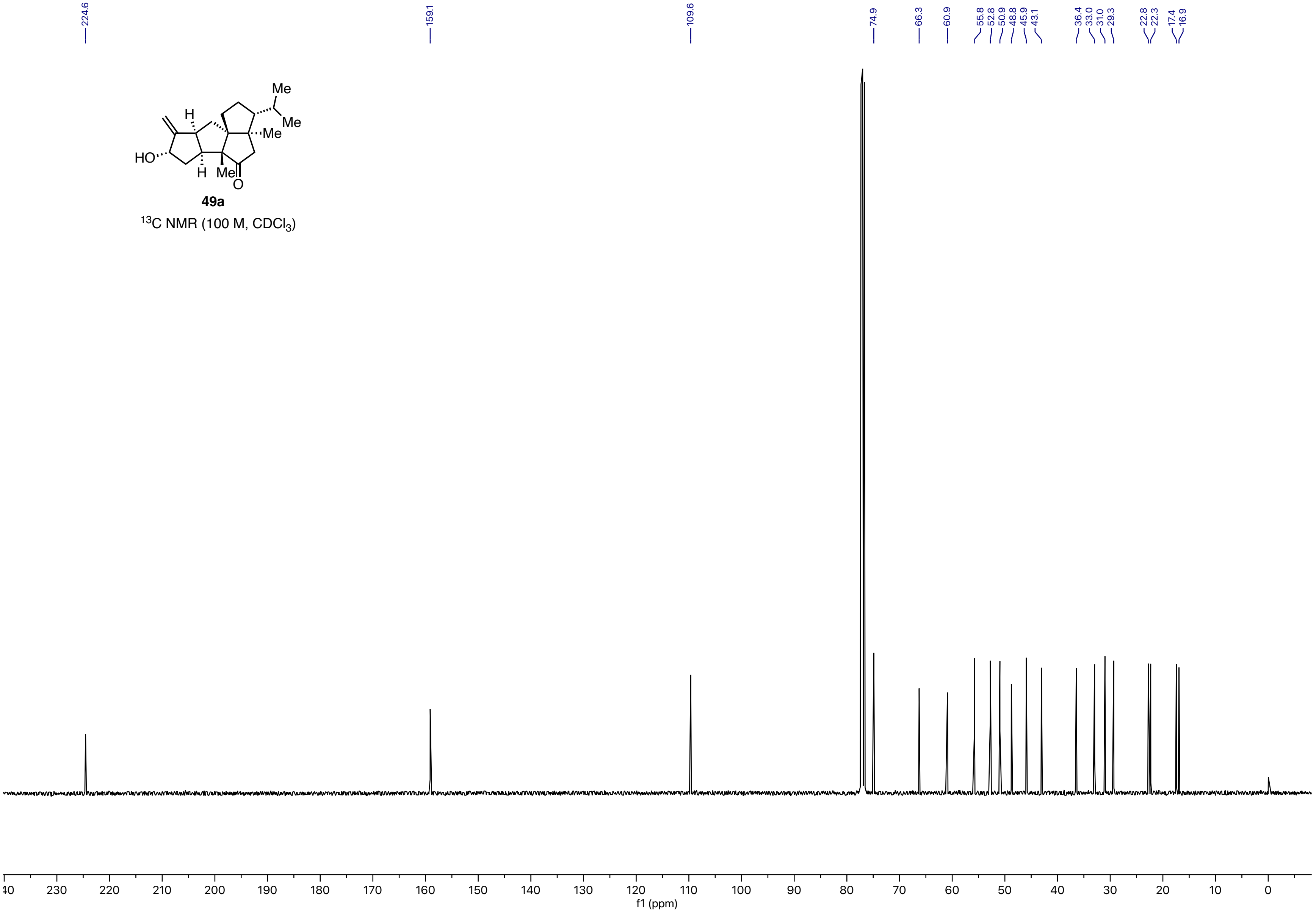
224.6
159.1
109.6
74.9
66.3
60.9
55.8
52.8
50.9
48.8
45.9
43.1
36.4
33.0
31.0
29.3
22.8
22.3
17.4
16.9
Me
Me
Me
H
HO
H
Me
O
49a
^{13}C NMR (100 M, $CDCl_3$)
f1 (ppm)

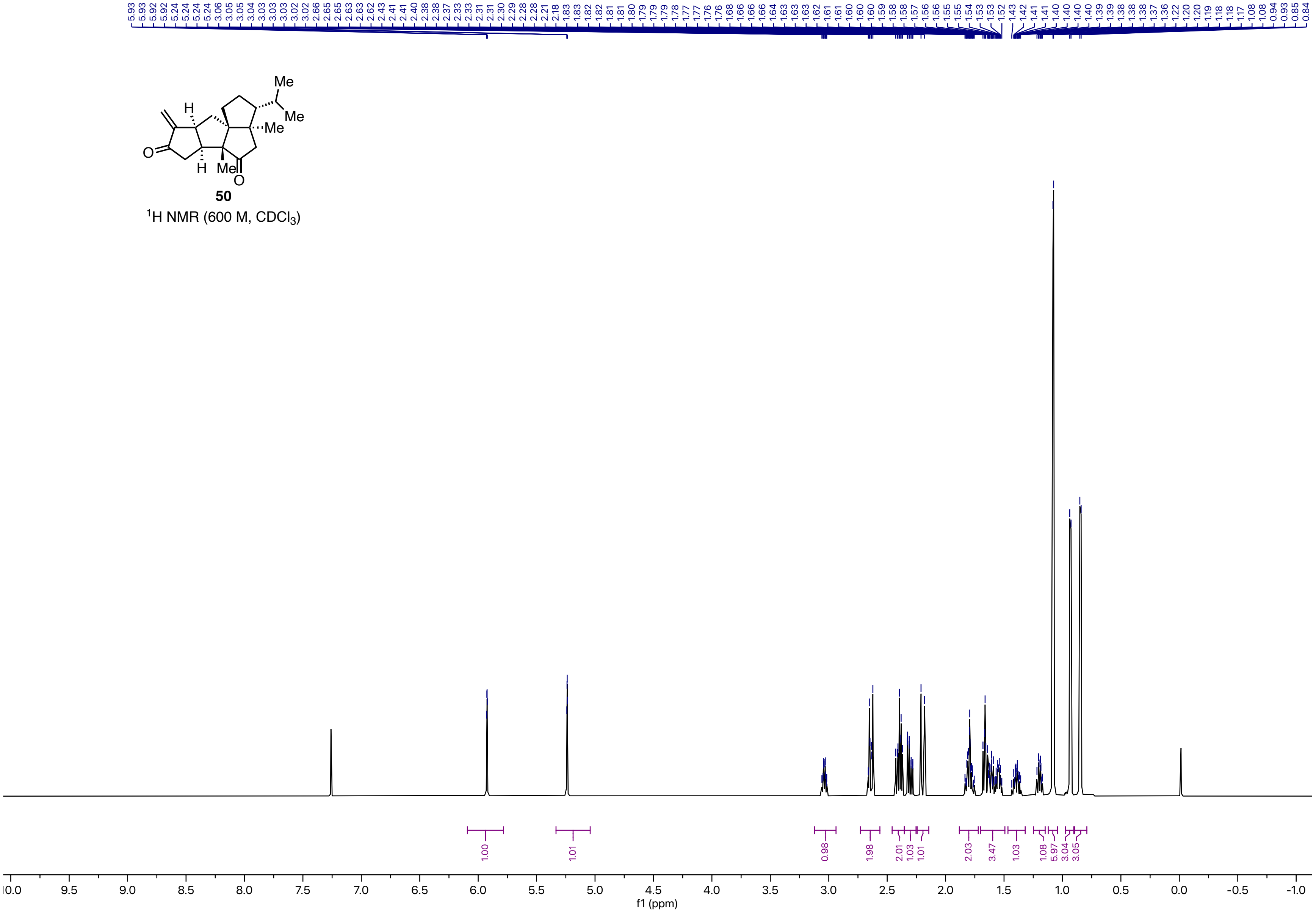
5.93 5.93 5.92 5.24 5.24 5.24 3.06 3.05 3.04 3.03 3.03 3.02 2.66 2.65 2.63 2.63 2.62 2.43 2.41 2.41 2.40 2.38 2.38 2.37 2.33 2.33 2.31 2.30 2.29 2.28 2.21 2.18 1.83 1.83 1.82 1.82 1.81 1.81 1.80 1.79 1.79 1.78 1.77 1.76 1.68 1.66 1.66 1.64 1.63 1.63 1.63 1.62 1.61 1.61 1.60 1.60 1.59 1.58 1.58 1.57 1.56 1.56 1.55 1.55 1.54 1.53 1.53 1.52 1.43 1.42 1.41 1.41 1.40 1.40 1.40 1.40 1.39 1.39 1.38 1.38 1.38 1.37 1.36 1.22 1.20 1.19 1.18 1.18 1.17 1.08 1.08 0.94 0.93 0.85 0.84
Me
H
Me
Me
O
H
Me
O
50
1H NMR (600 M, CDCl3)
1.00
1.01
0.98
1.98
2.01
1.03
1.01
2.03
3.47
1.03
1.08
5.97
3.04
3.05
10.0 9.5 9.0 8.5 8.0 7.5 7.0 6.5 6.0 5.5 5.0 4.5 4.0 3.5 3.0 2.5 2.0 1.5 1.0 0.5 0.0 -0.5 -1.0
f1 (ppm)

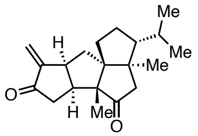

50

^{13}C NMR (150 M, $CDCl_3$)

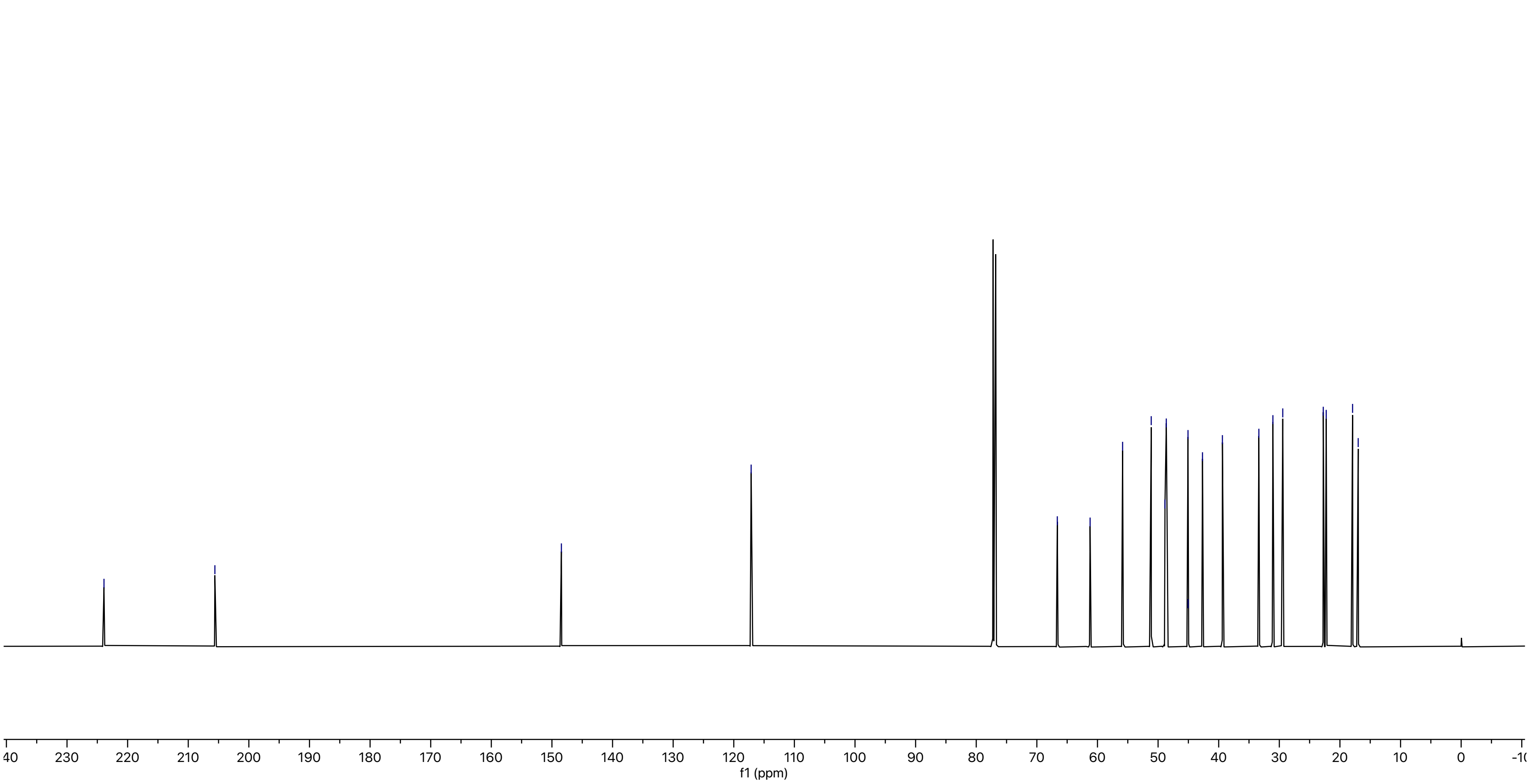

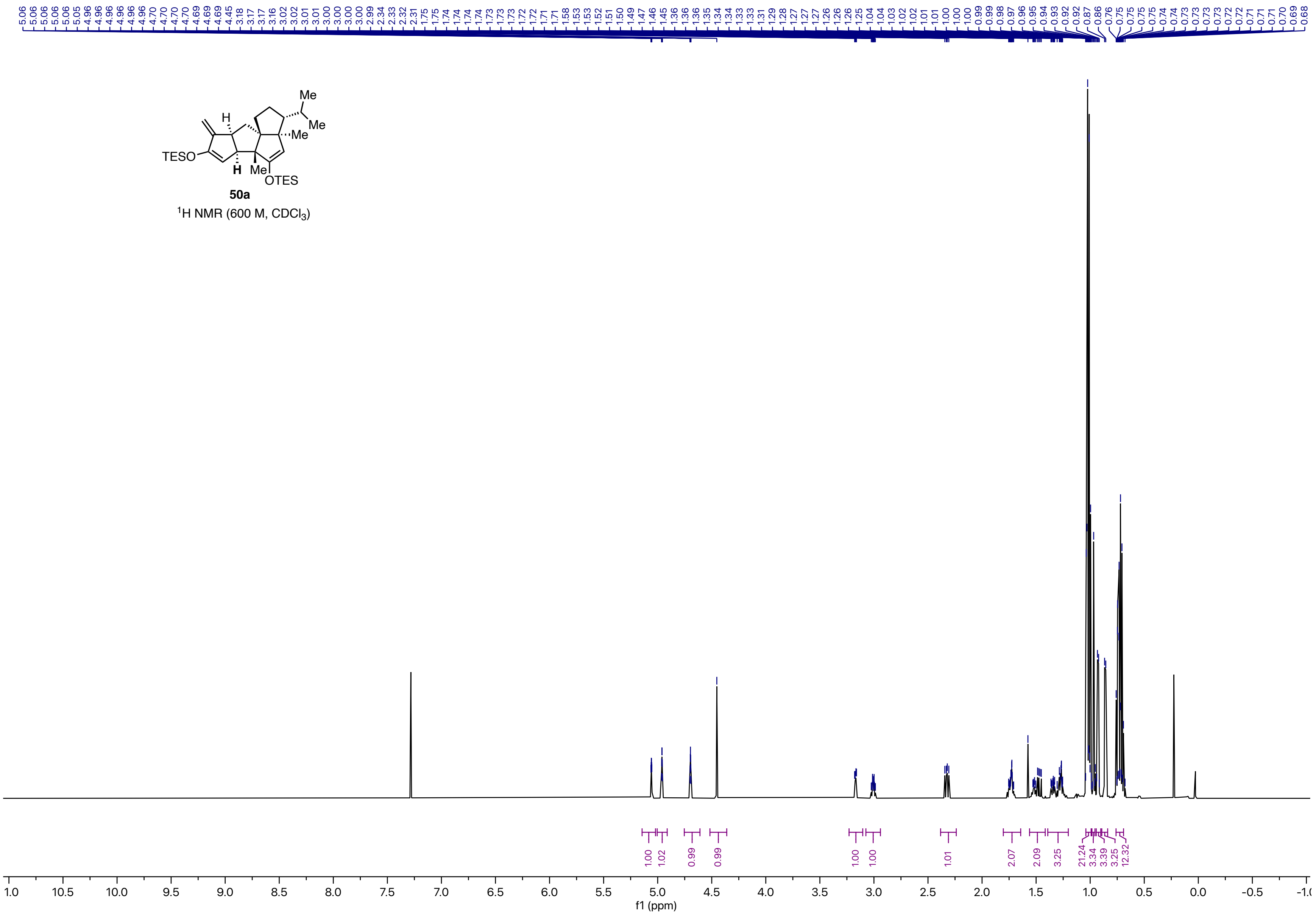
5.06 5.06 5.06 5.06 5.05 4.96 4.96 4.96 4.96 4.70 4.70 4.70 4.69 4.69 4.69 4.45 3.18 3.17 3.17 3.16 3.02 3.01 3.01 3.00 3.00 3.00 2.99 2.34 2.33 2.32 2.31 1.75 1.75 1.74 1.74 1.74 1.73 1.73 1.72 1.71 1.71 1.58 1.53 1.53 1.52 1.51 1.50 1.49 1.47 1.46 1.45 1.36 1.36 1.35 1.34 1.34 1.33 1.33 1.31 1.29 1.28 1.27 1.27 1.26 1.26 1.25 1.04 1.04 1.03 1.02 1.02 1.01 1.01 1.00 1.00 1.00 0.99 0.99 0.98 0.97 0.96 0.95 0.94 0.93 0.92 0.92 0.87 0.86 0.76 0.75 0.75 0.75 0.75 0.74 0.74 0.73 0.73 0.73 0.72 0.72 0.71 0.71 0.70 0.69 0.68
Me
H
Me
Me
TESO
H
Me
OTES
50a
^{1}H NMR (600 M, CDCl$_3$)
1.00 1.02 0.99 0.99 1.00 1.00 1.01 2.07 2.09 3.25 21.24 3.34 3.39 3.25 12.32
1.0 10.5 10.0 9.5 9.0 8.5 8.0 7.5 7.0 6.5 6.0 5.5 5.0 4.5 4.0 3.5 3.0 2.5 2.0 1.5 1.0 0.5 0.0 -0.5 -1.(
f1 (ppm)

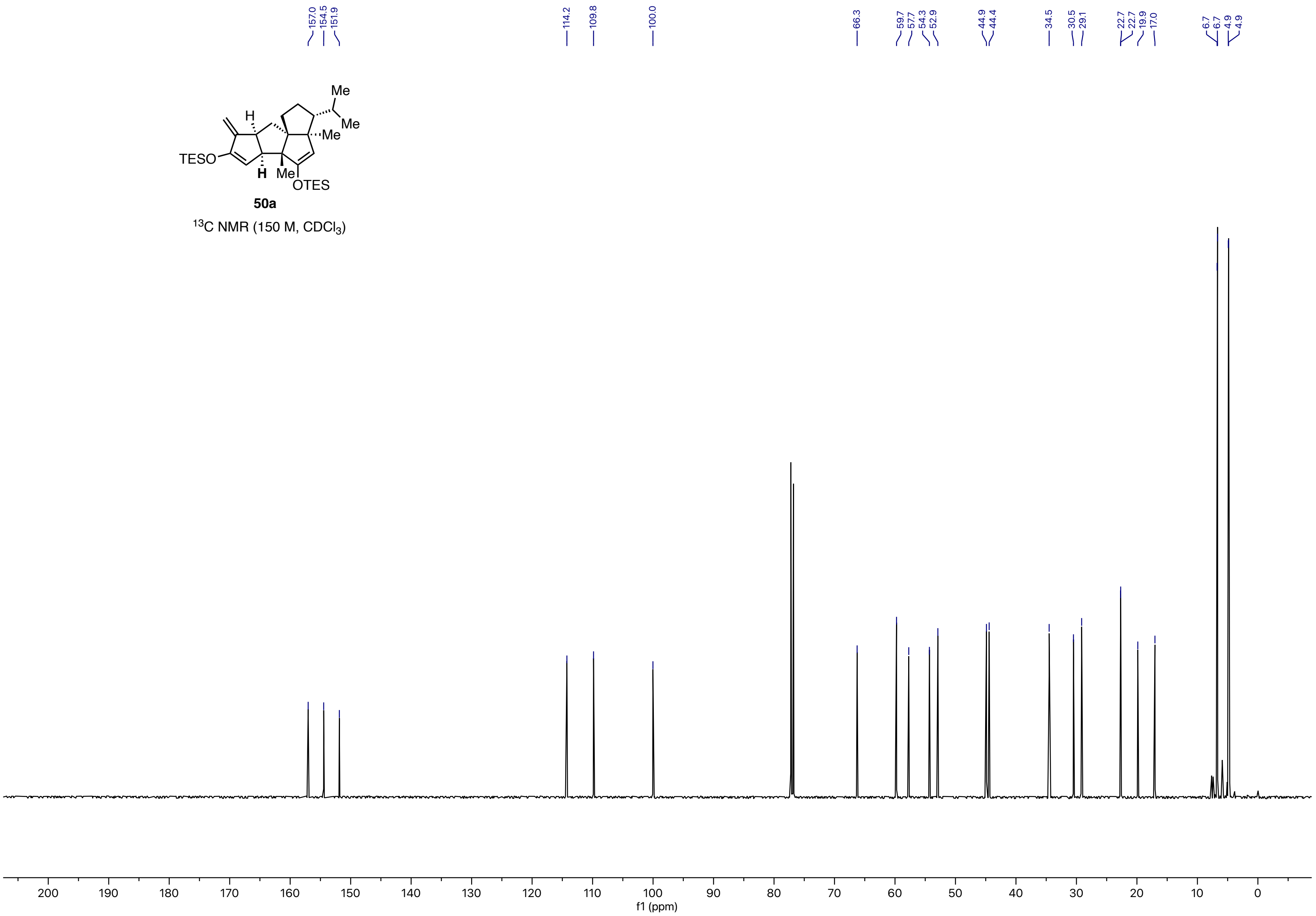

50a

^{13}C NMR (150 M, $CDCl_3$)

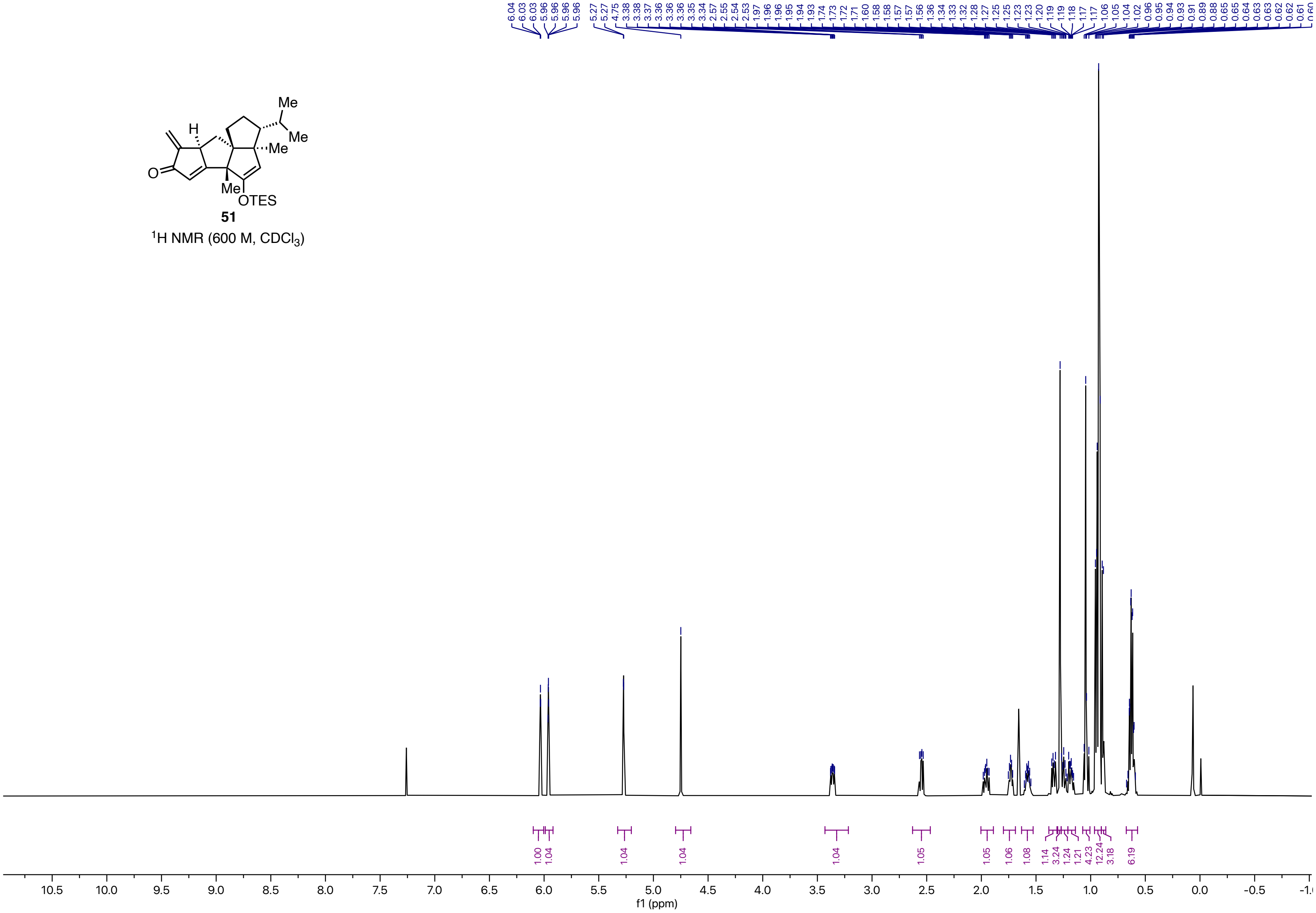

51

^{1}H NMR (600 M, $CDCl_3$)

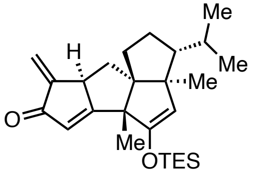

51

^{13}C NMR (150 M, $CDCl_3$)

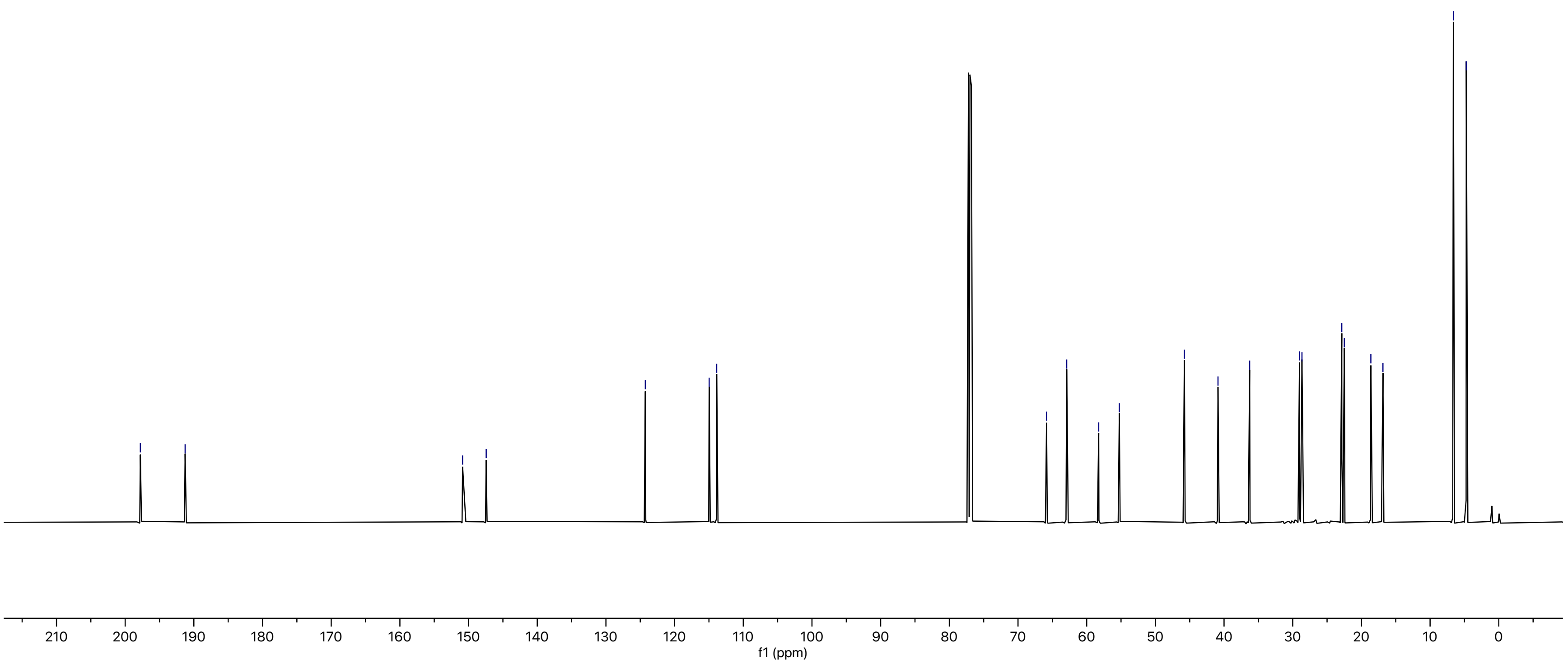

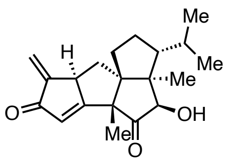

53

^{1}H NMR (600 M, $CDCl_3$)

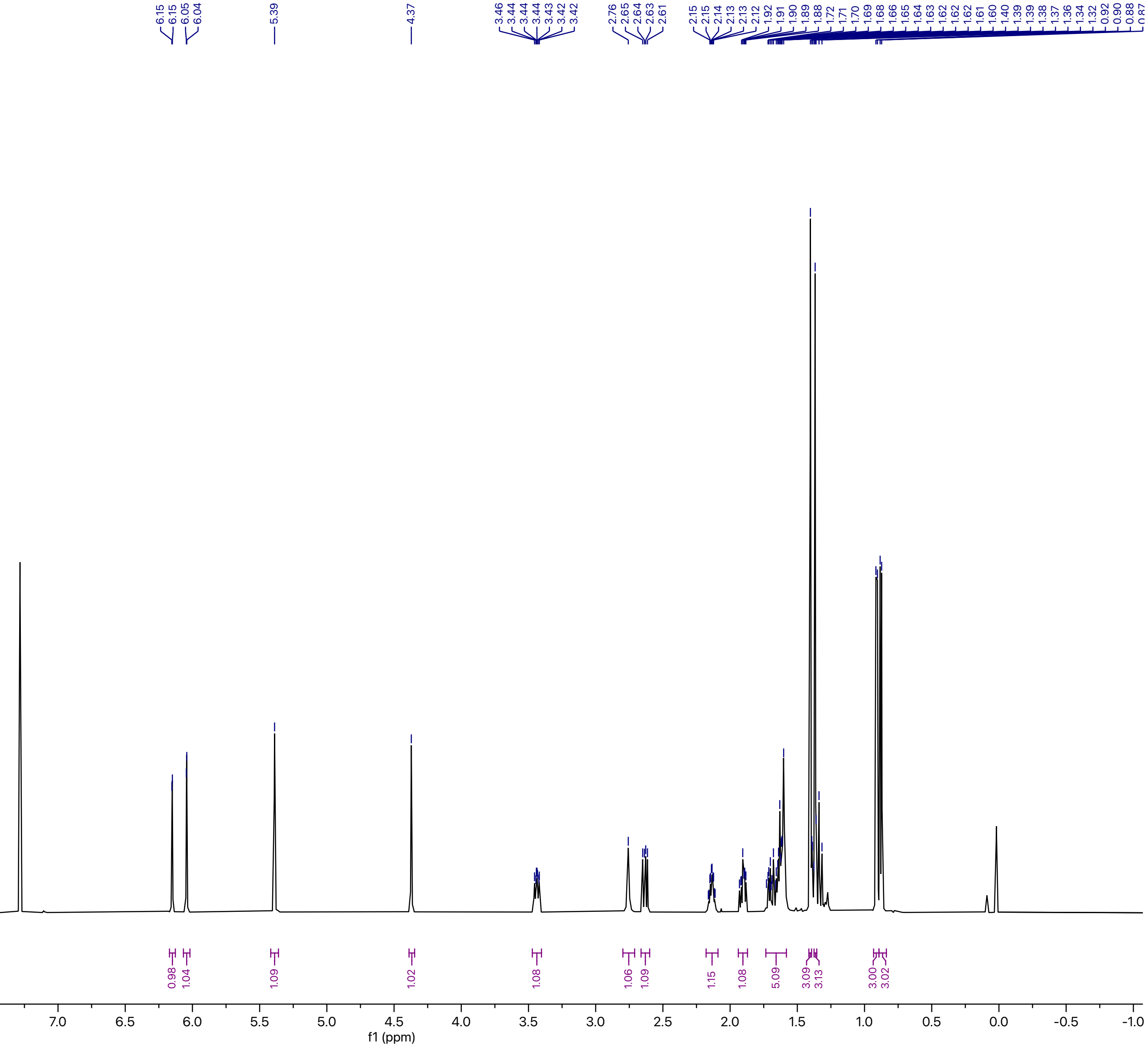

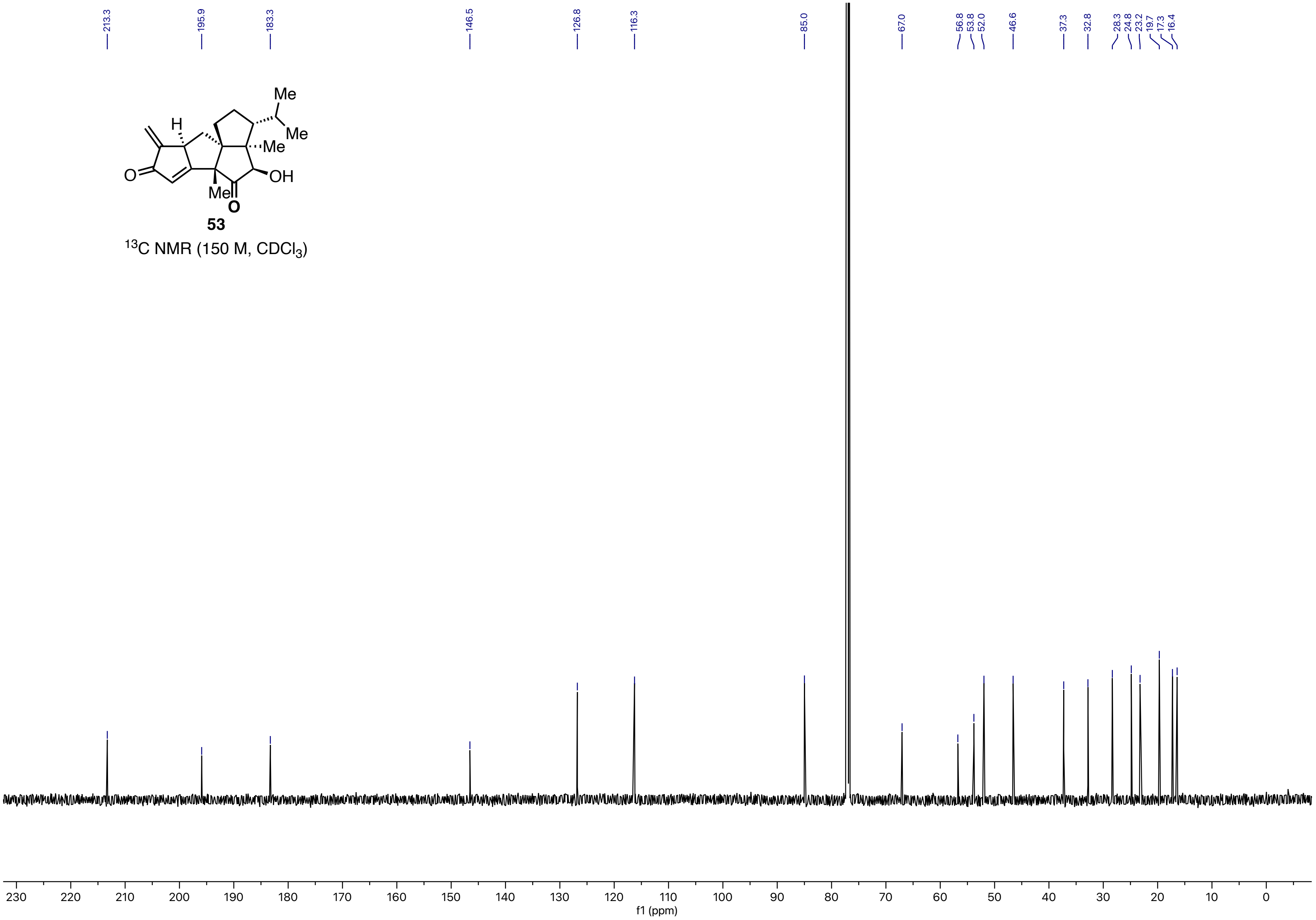

53

^{13}C NMR (150 M, $CDCl_3$)

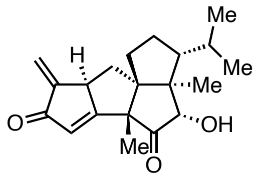

52

^{1}H NMR (400 M, $CDCl_3$)

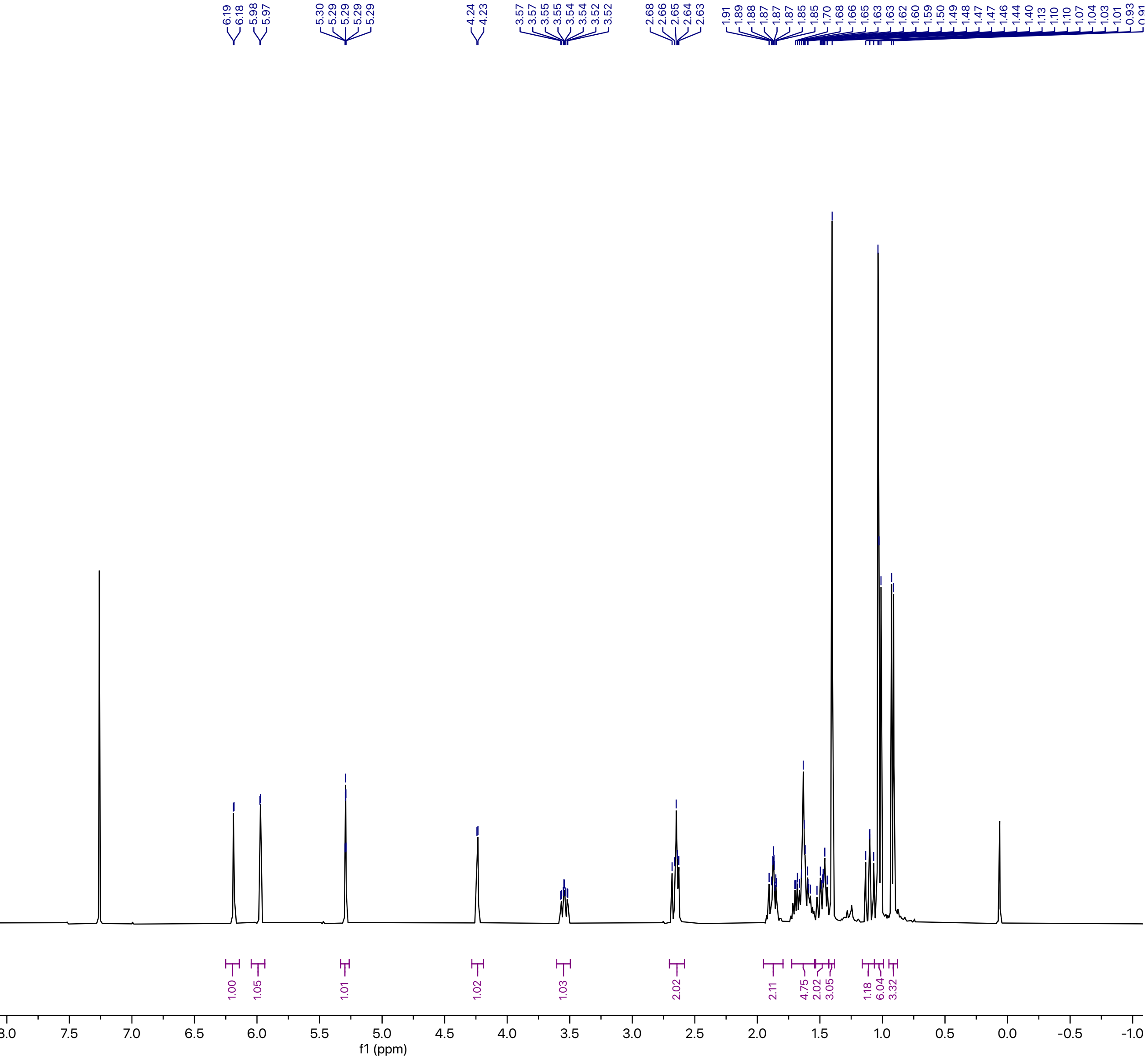

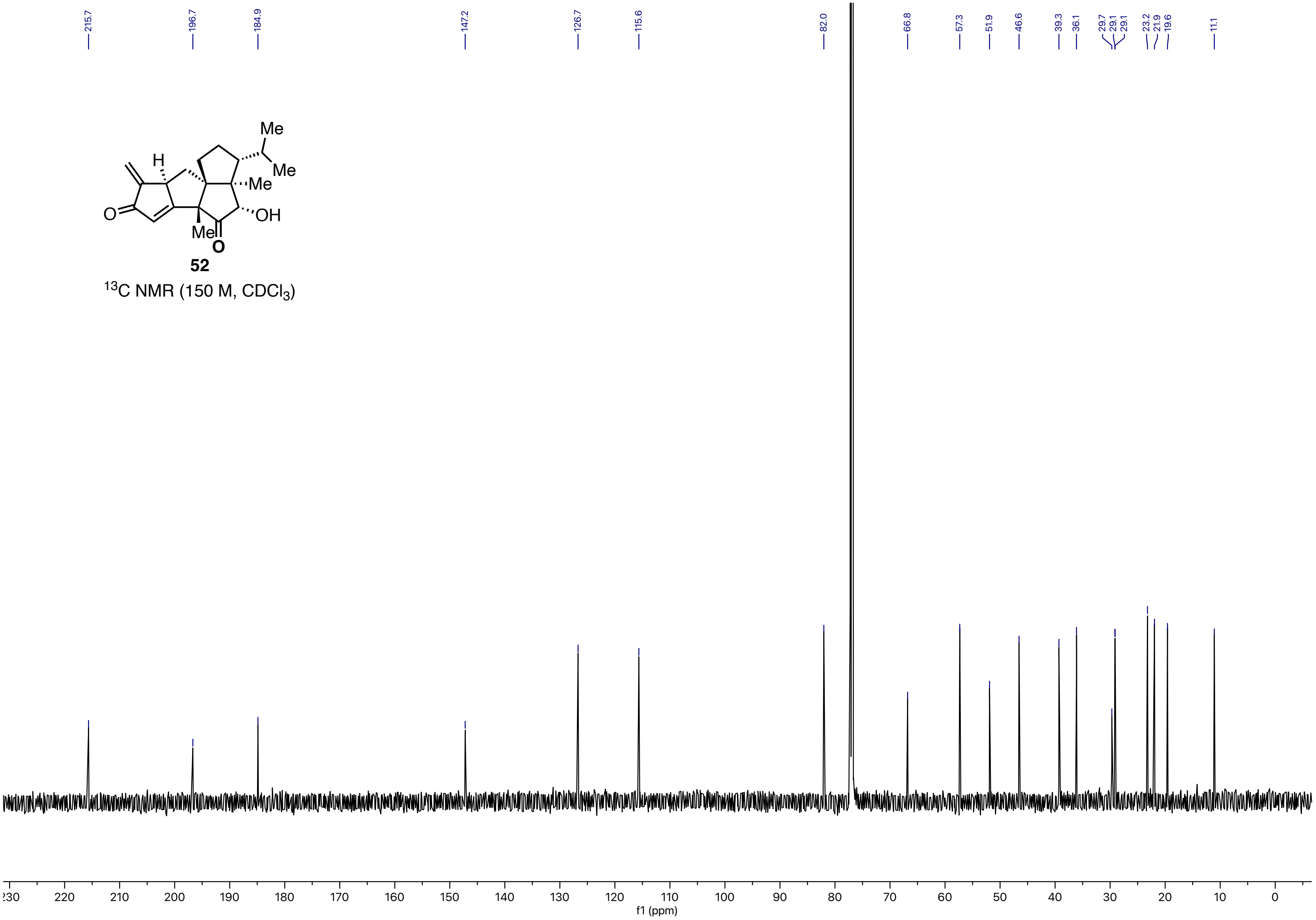
215.7
196.7
184.9
147.2
126.7
115.6
82.0
66.8
57.3
51.9
46.6
39.3
36.1
29.7
29.1
29.1
23.2
21.9
19.6
11.1
Me
H
Me
Me
O
OH
Me
O
52
^{13}C NMR (150 M, $CDCl_3$)
230 220 210 200 190 180 170 160 150 140 130 120 110 100 90 80 70 60 50 40 30 20 10 0
f1 (ppm)

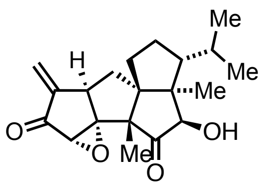

Crinipellin A (**1**)

^{1}H NMR (400 M, $CDCl_3$)

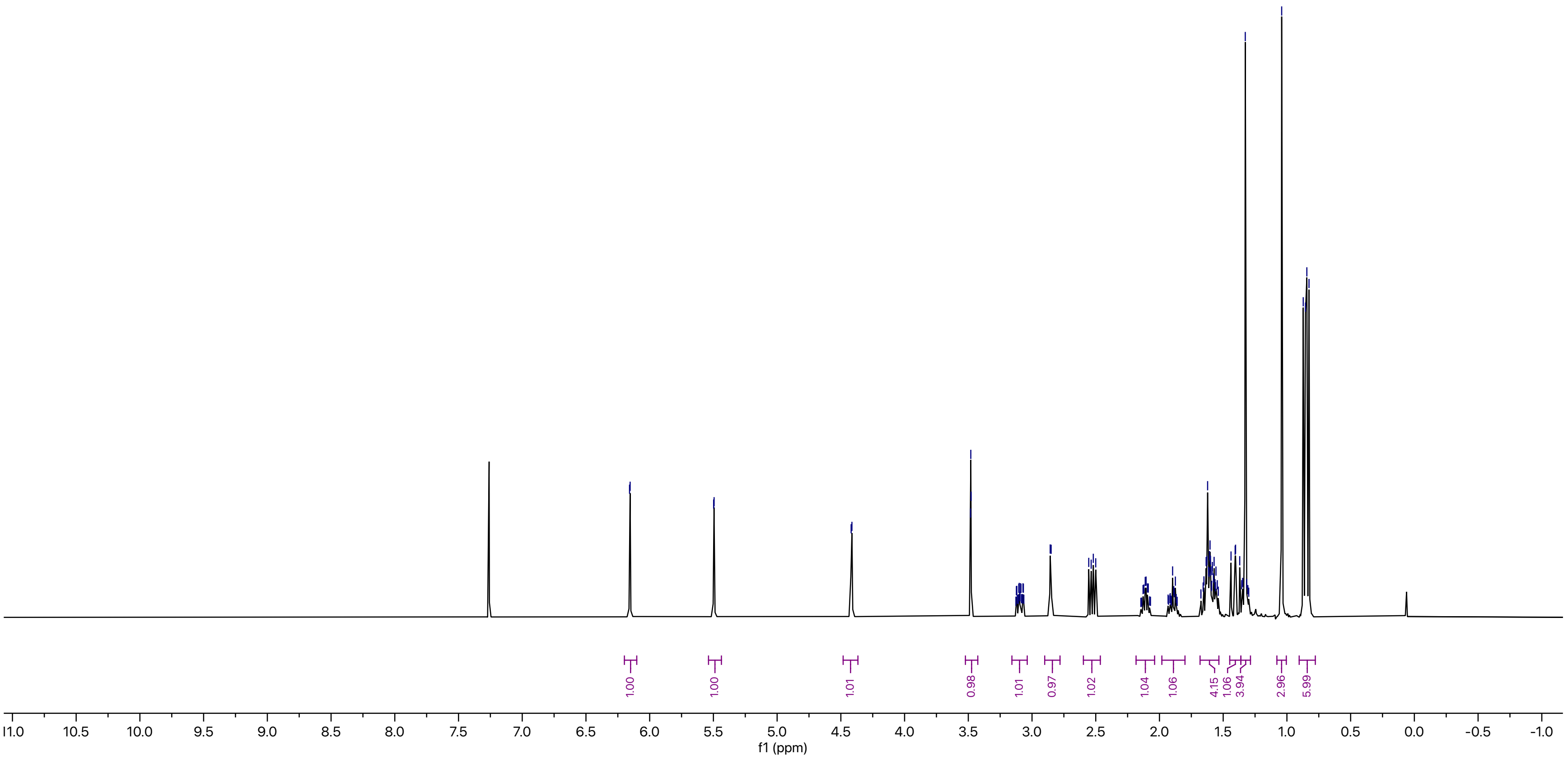

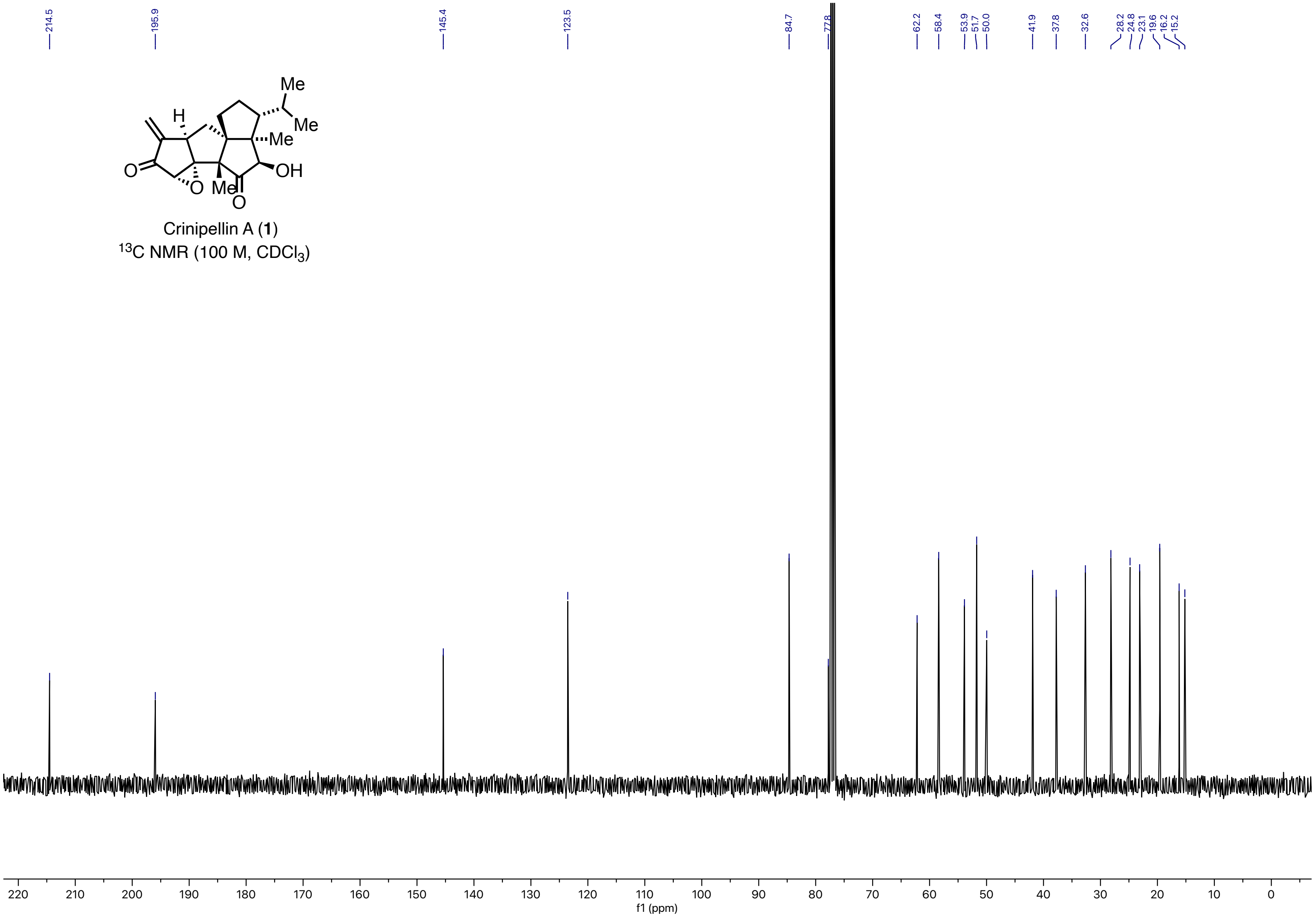
214.5
195.9
145.4
123.5
84.7
77.8
62.2
58.4
53.9
51.7
50.0
41.9
37.8
32.6
28.2
24.8
23.1
19.6
16.2
15.2
Me
H
Me
Me
O
OH
O
Me
O
Crinipellin A (1)
^{13}C NMR (100 M, $CDCl_3$)
220 210 200 190 180 170 160 150 140 130 120 110 100 90 80 70 60 50 40 30 20 10 0
f1 (ppm)

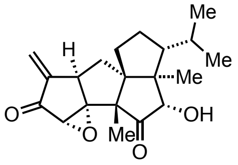

54

^{1}H NMR (400 M, $CDCl_3$)

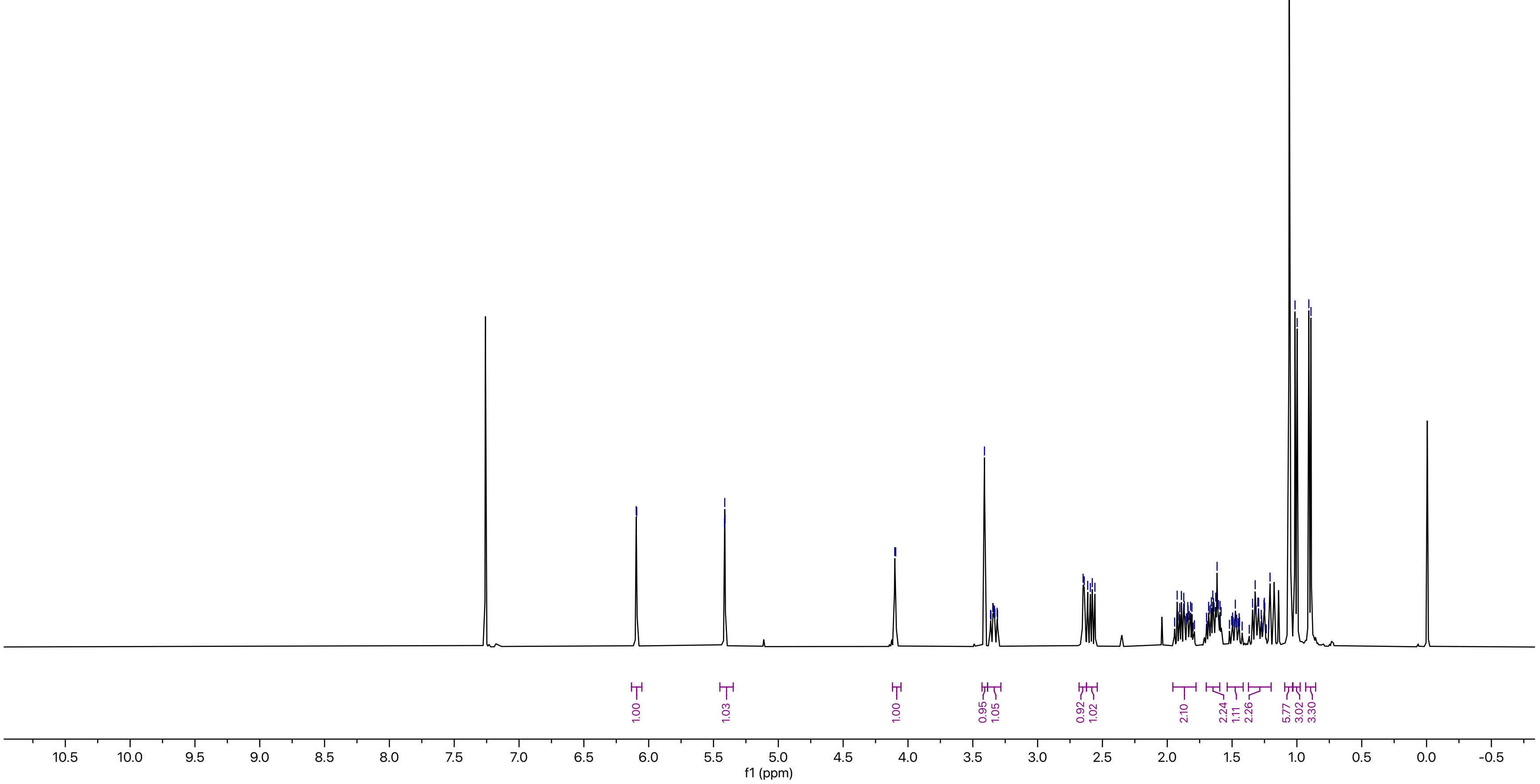

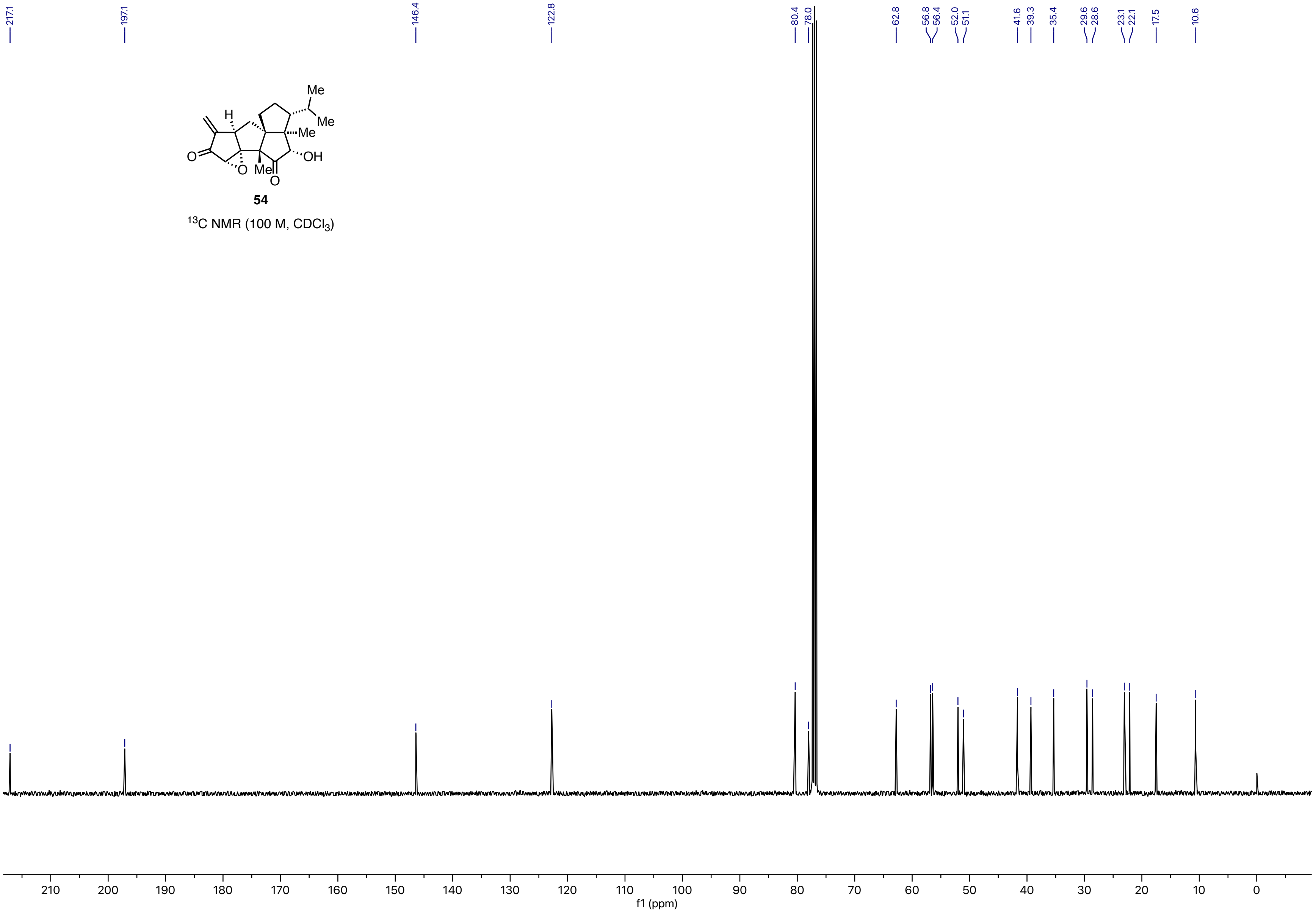
217.1
197.1
146.4
122.8
80.4
78.0
62.8
56.8
56.4
52.0
51.1
41.6
39.3
35.4
29.6
28.6
23.1
22.1
17.5
10.6
Me
H
Me
Me
O
OH
O
Me
O
54
^{13}C NMR (100 M, $CDCl_3$)
210 200 190 180 170 160 150 140 130 120 110 100 90 80 70 60 50 40 30 20 10 0
f1 (ppm)

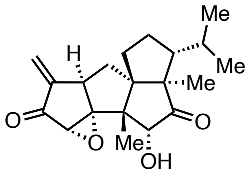

Crinipellin B (**2**)

^{13}C NMR (100 M, $CDCl_3$)

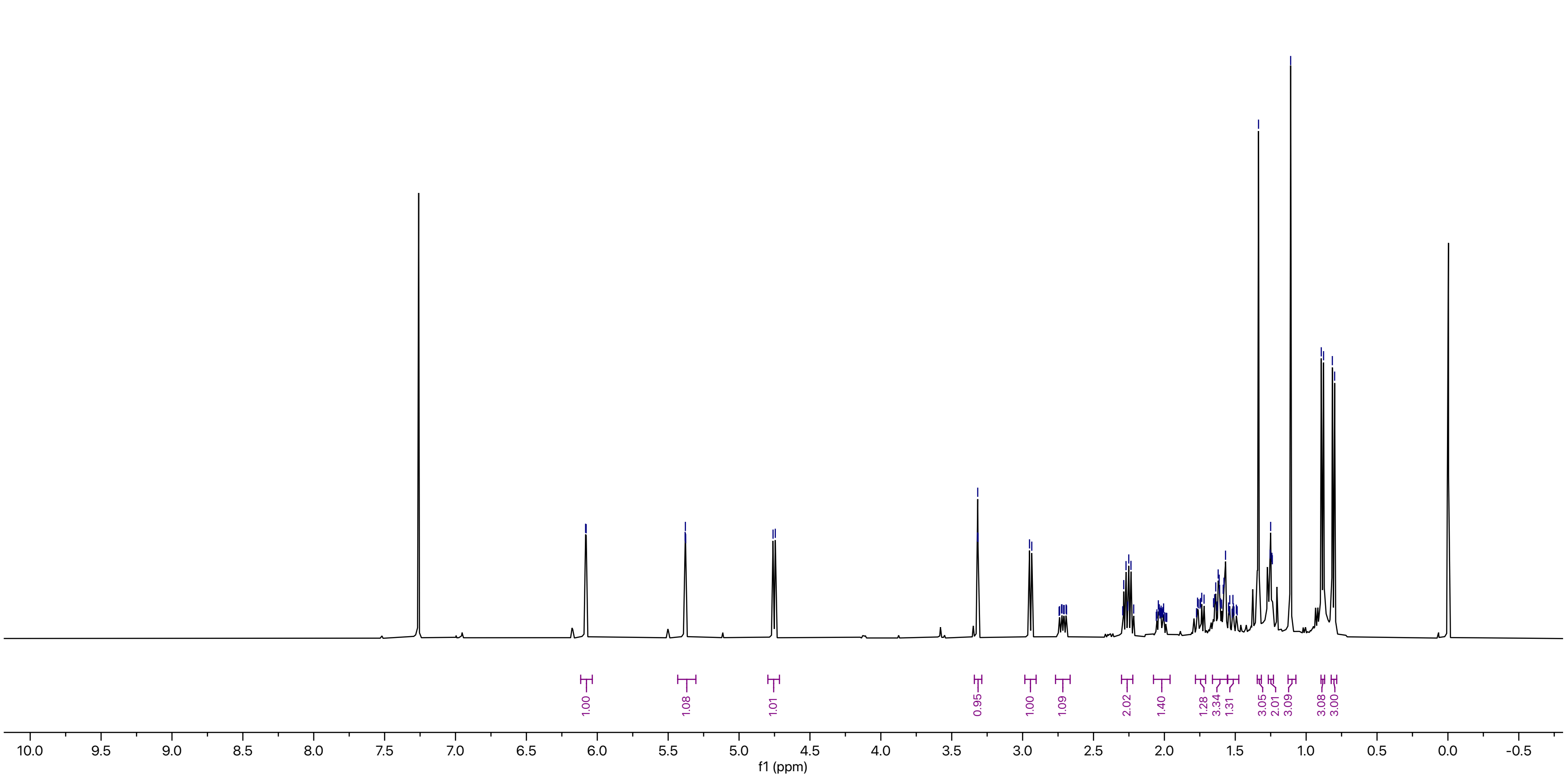

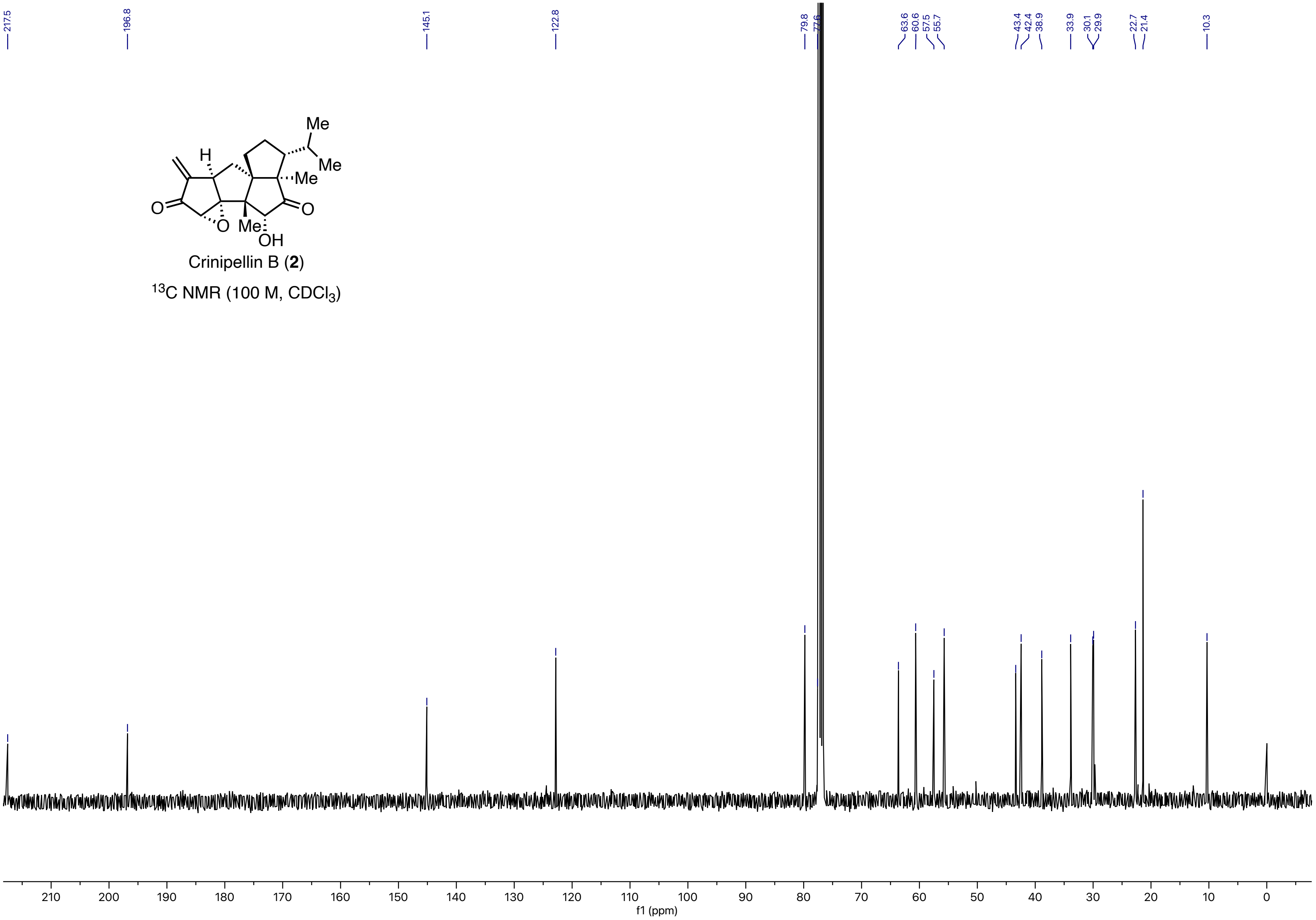

Crinipellin B (**2**)

^{13}C NMR (100 M, $CDCl_3$)
